# Late-Stage Functionalization of Lysine to Organelle-Targeting Fluorescent Probes

**DOI:** 10.1021/jacsau.5c00680

**Published:** 2025-08-16

**Authors:** Patricia Rodriguez, Ankita Misra, Monika Raj

**Affiliations:** Department of Chemistry, 1371 Emory University, Atlanta, Georgia 30322, United States

**Keywords:** late-stage functionalization, peptides, fluorescence, delocalized lipophilic cation, mitochondria-targeting

## Abstract

The late-stage functionalization of peptides presents a promising avenue for expanding their chemical diversity and properties, particularly in the realm of drug discovery. Herein, we present a powerful late-stage functionalization (LSF) strategy for peptides that includes an addition-cyclization-aromatization (ACA) transformation to generate 2,3,4,6-substituted pyridinium, with inherent fluorescence, specifically at lysine residues. This method enables the precise and irreversible labeling of diverse peptide sequences, both in solution and on solid support, with quantum yields ranging from 0.02 to 0.03. Importantly, the 2,3,4,6-substituted pyridinium core represents a delocalized lipophilic cation (DLC), exhibiting strong mitochondrial-targeting properties. This unique platform facilitates real-time imaging and targeted delivery of drugs and peptides to mitochondria without the need for additional tagging, offering significant potential for theranostic applications.

## Graphical abstract

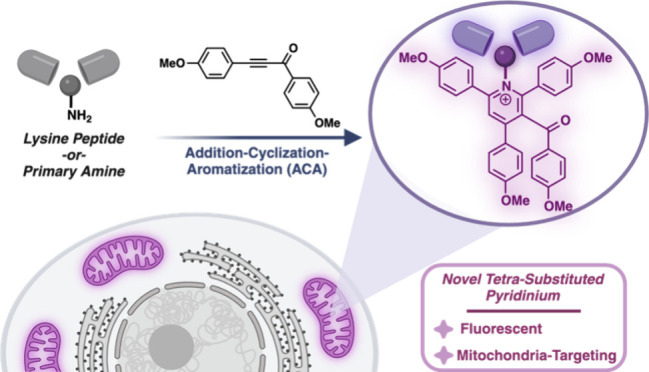

## Introduction

Mitochondria are critical organelles in cellular energy production, generating the ATP necessary for cellular functions and initiating key signaling pathways, including those involved in programmed cell death.^1,2^ Given their central role in metabolism and survival, mitochondrial dysfunction is implicated in a variety of diseases, including cardiovascular disorders, neurodegenerative diseases, and cancer.^3,4^ This has spurred significant interest in targeting mitochondria for therapeutic purposes, particularly in cancer treatment.^5–10^ Over the past decade, a range of mitochondria-targeting agents—such as small-molecule delocalized lipophilic cations (DLCs), transition metal complexes, peptides, and nanoparticles—have been developed for selective delivery to mitochondria, improving therapeutic efficacy and overcoming drug resistance by preventing drug efflux.^11–21^ Among these, fluorescent mitochondria-targeting DLCs have emerged as promising candidates for theranostic applications, offering both therapeutic benefits and real-time imaging capabilities.^22–24^ However, while advances have been made, a major challenge remains in the development of peptide-based therapeutics that can simultaneously deliver both mitochondrial-targeting and fluorescent imaging functionalities.^25^

Late-stage functionalization (LSF) strategies are increasingly recognized as a powerful tool in drug discovery, enabling the introduction of diverse properties at the final stages of synthesis, such as improved cell permeability, stability, and binding affinity.^26,27^ This makes LSF a particularly attractive method for developing mitochondria-targeting peptides with enhanced therapeutic and diagnostic features. However, current techniques for the synthesis of mitochondria-targeting peptides face a limitation with efficient incorporation of both fluorescence and mitochondria-specific targeting moieties in a single, streamlined process.^28–31^ Existing fluorescent mitochondria-targeting peptides—such as the Szeto-Schiller (SS) peptide, L-1P/D-1P, and Mito-FF—often require multiple synthetic steps to separately attach targeting moieties and fluorophores, making the process complex and time-consuming (Figure 1A).^32–36^ Expanding the LSF toolbox to simultaneously incorporate imaging and targeting functionalities promotes the utility of mitochondria-targeting peptides in cancer therapy and other diseases.

**Figure 1:**
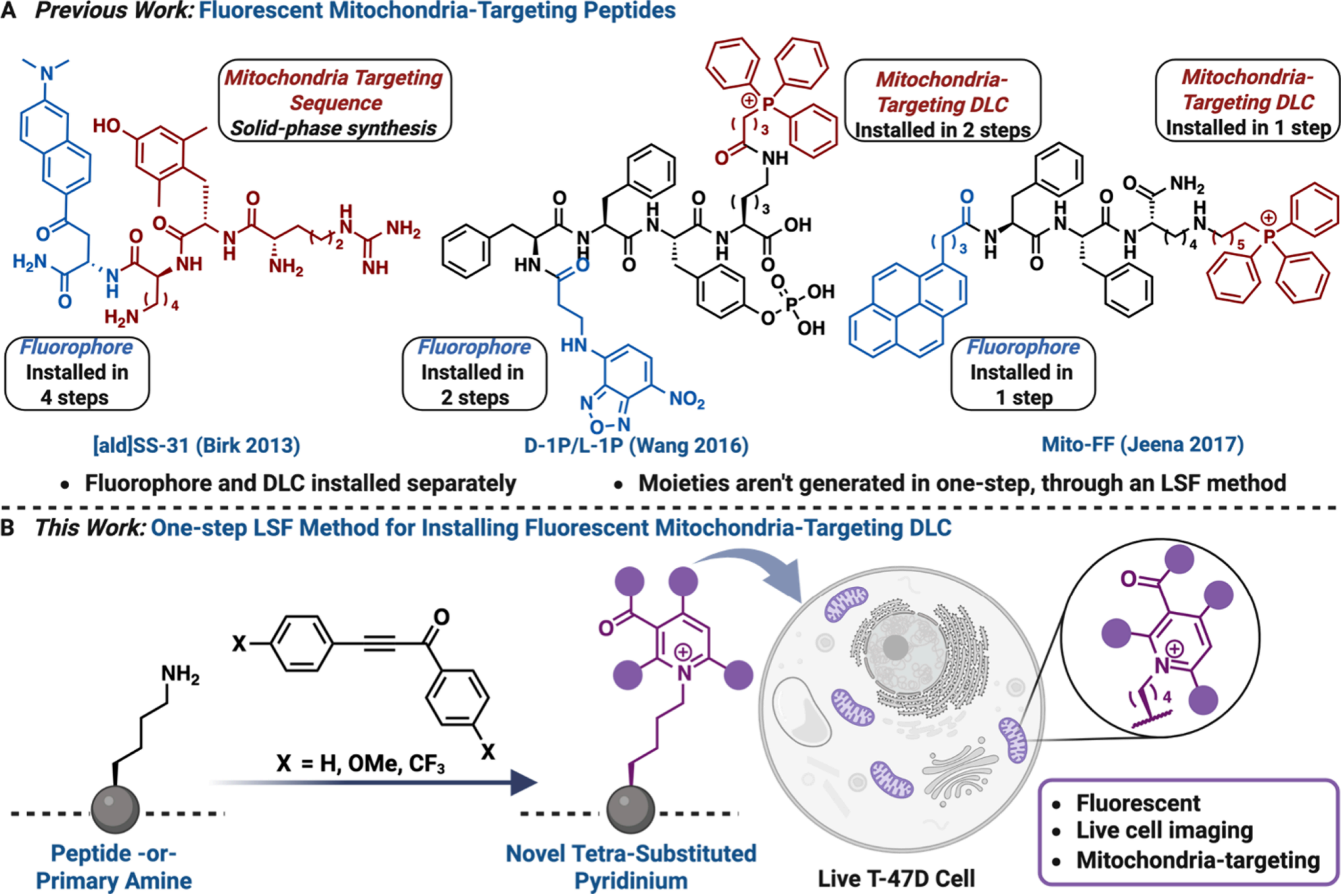
One-step synthesis of fluorescent mitochondria-targeting peptide. (A) Previous methods for adding fluorescent and mitochondria-targeting groups on peptides require multistep synthesis and two different functional moieties. (B) One-step installation of the functional moiety on lysine displaying both fluorescent and mitochondria-targeting properties. Created with permission from BioRender.com.

Herein, we introduce an LSF strategy for lysine (Lys) residues that installs a fluorescent mitochondria-targeting DLC on unprotected complex peptides via a nonfluorescent ynone (Figure 1B). This strategy unfolds through a unique three-step process: first, the **addition** of lysine to the ynone, followed by **cyclization** of the intermediate, and finally **aromatization** to form diverse 2,3,4,6-substituted pyridinium derivatives exclusively at lysine residues, termed the addition-cyclization-aromatization (ACA) strategy. Leveraging the intrinsic fluorescent properties of these 2,3,4,6-substituted pyridinium cores, we explored the impact of varying electronic structures of substituents and achieved quantum yields ranging from 0.02 to 0.03. We applied the ACA method for the LSF of peptides with varying sequences in both solution and on solid support. Importantly, we demonstrated the application of ACA-modified peptides for imaging and selective delivery to mitochondria in live cells while retaining their original function. Additionally, we showed the compatibility of ACA with small-molecule anticancer drugs such as doxorubicin by successful delivery to mitochondria.

In summary, the ACA-LSF platform provides a new powerful tool for peptide diversification, offering exclusive selectivity for lysine in generating a novel fluorescent mitochondria-targeting scaffold with significant potential for advanced therapeutic monitoring and selective drug delivery.

## Results and Discussion

### Development of the Addition-Cyclization-Aromatization (ACA) Method

Inspired by work published on Katritzky salts, specifically their interesting fluorescent properties and delocalized lipophilic cationic nature, we hoped to develop a method that could successfully install phenyl-substituted pyridiniums on complex, unprotected peptides. Previous methods for synthesizing Katritzky salt peptides include the reaction of 2,4,6-triphenylpyrylium salts with protected amino acids or simple protected peptides, limiting the substrate scope and applications.^37^ In our pursuit to develop a method for the selective installation of phenyl-substituted pyridiniums on complex, unprotected peptides, we utilized an electron-deficient ynone due to its ability to produce reactive alpha-beta unsaturated ketones upon a double Michael addition reaction with lysine. This subsequently allows for intramolecular cyclization of the alpha-beta unsaturated ketones, followed by aromatization to produce a stable pyridinium core (Figures 2A and S1). This property facilitates the formation of irreversible pyridinium complexes with lysine. Initially, we optimized the ACA reaction using a lysine-containing model peptide, AcLILKPF **2a**. The peptide was subjected to 6 and 9 equiv of ynone 1a under different reaction conditions (solvent, temperature, and time) (Figures 2B and S2). The optimal conditions to obtain the desired lysine pyridinium **3a** were 6 equiv of the ynone, with a solvent system of 1:1 MeCN/NaP and NaHCO_3_ buffer (pH = 8), at 80 °C for 15 h (entry 5, Figure 2B). Reaction conditions shown in entries 6–9 led to slightly improved or similar conversion for pyridinium **3a**, but more byproducts and degradants were observed during reaction analysis. To determine the importance of base and a protic solvent for the ACA reaction, it was attempted in MeCN/water (1:1) and the presence of a moderately strong inorganic base (entry 9, Figure 2B), and in an aprotic solvent and the presence of an organic amine base (entry 10, Figure 2B). No lysine pyridinium **3a** was observed for entry 10, which indicates that a protic solvent is necessary for cyclization.

**Figure 2:**
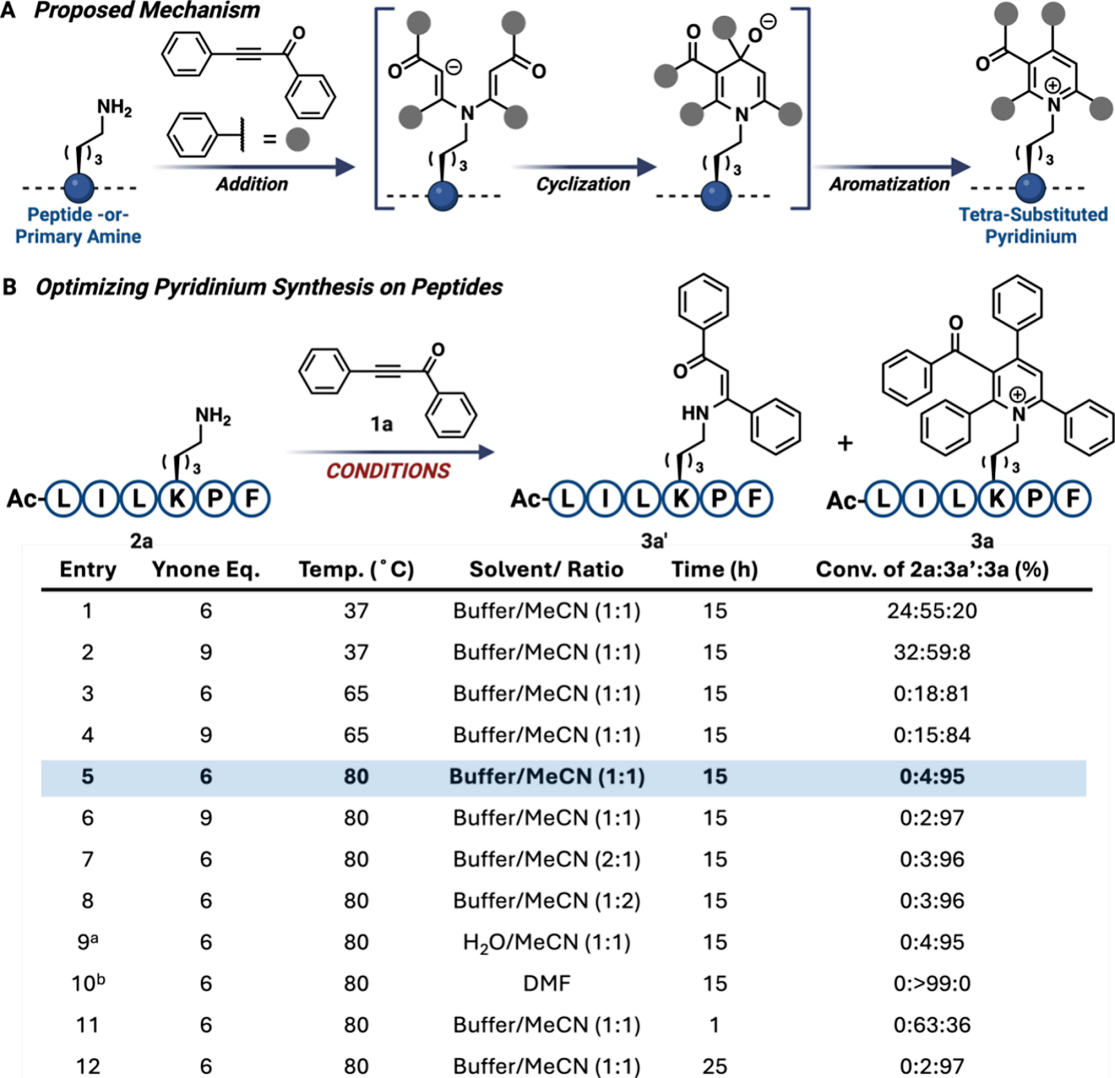
One-step conversion of lysine to tetrasubstituted pyridinium. (A) Unique three-step process involving the addition of lysine to the ynone **1a**, followed by cyclization of the intermediate, and aromatization to form diverse 2,3,4,6-substituted pyridinium derivatives exclusively at lysine residues, termed addition-cyclization-aromatization (ACA) strategy. (B) Optimization of the reaction conditions for the synthesis of 2,3,4,6-substituted pyridinium on peptides. ^a^K_2_CO_3_ (1 equiv) was added. ^b^DIPEA (1 equiv) was added. Percent conversion was determined by HPLC and LC-MS. Created with BioRender.com.

Given the multistep nature of this reaction, including two Michael addition reactions on lysine, followed by intramolecular cyclization on alpha-beta unsaturated ketones, dehydration, and aromatization to generate pyridinium **3a**, we aimed to capture intermediates by analyzing the reaction at regular intervals. Interestingly, we did not observe the formation of the double Michael addition product, primarily due to its instability associated with steric hindrance. Instead, after 1 h, we detected significant amounts of the mono Michael addition product **3a′** (63%) alongside pyridinium product **3a** (36%), which eventually converted to more than 90% pyridinium product in 15 h (Figure 2B, entry 5 and 11–12, Figure S2). These experiments suggested that once double Michael adducts form, they spontaneously convert to stable pyridinium product **3a**. To further investigate and enhance our proposed mechanism, we synthesized the monoalkylated intermediate using a small-molecule model, butylamine (Figure S3). The monoalkylated intermediate **4a**′**** was subjected to ACA, and conversion to pyridinium **4a** was analyzed at several time intervals over 24 h (Figure S4). After 24 h, most of the monoalkylated intermediate **4a**′**** converted to pyridinium **4a** (90%), confirming that the monoalkylated amine is a true intermediate of ACA. Additionally, we synthesized the pyridinium product **4a** on a larger scale and characterized it using NMR (Figures 3A, S5, and S6).

**Figure 3:**
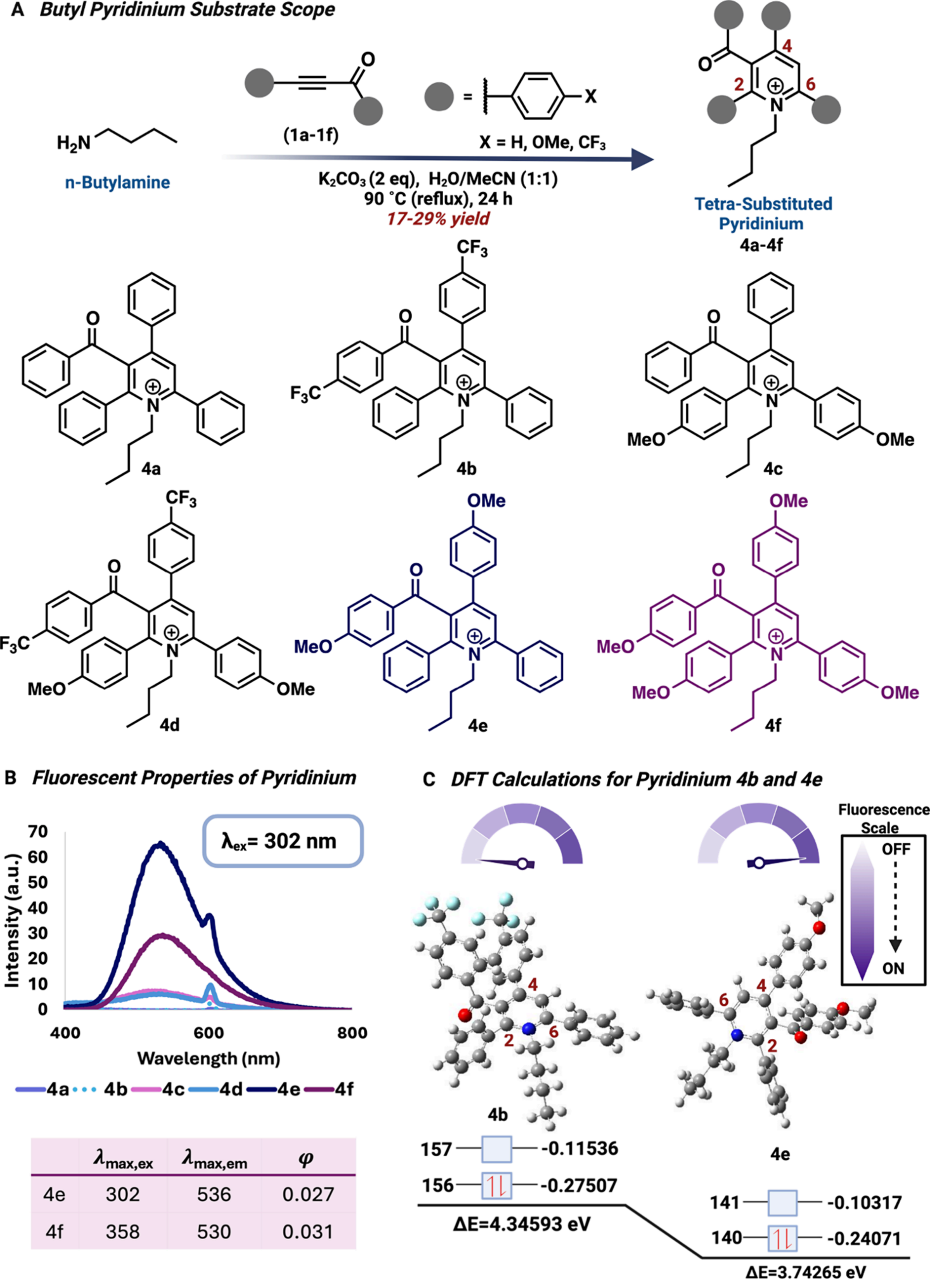
Fluorescent properties of diverse substituted tetrasubstituted pyridinium. (A) Diverse tetrasubstituted pyridinium with varying EWG and EDG groups. (B) Fluorescent properties of diverse tetrasubstituted pyridinium with compounds **4e** and **4f** exhibiting quantum yields of 0.027 and 0.031. (C) DFT calculations explaining the correlation between fluorescence and Δ*E* of the lower energy conformers of **4e** and **4b**. Created with BioRender.com.

### Exploring Fluorescent Properties and Small-Molecule Substrate Scope

Due to the extensive conjugation present in the resulting 2,3,4,6-substituted pyridinium and their structural resemblance to Kartitzky salts, we hypothesized that this molecule might exhibit fluorescence activity. To test this hypothesis, we recorded the fluorescence of small-molecule-derived pyridinium core **4a**, obtained from the reaction between butylamine and ynone **1a.**

However, we observed negligible fluorescent properties. On a molecular level, fluorescence is heavily influenced by electronic transitions and subsequent relaxation processes.^38^ We speculated that the inherent electronic structure of the parent compound might not support efficient photon absorption and emission, leading to weak fluorescence. This could potentially be attributed to the absence of extensive conjugation or the lack of electron-donating (EDG) or electron-withdrawing (EWG) groups, which could optimize the molecule for better absorbance and emission characteristics. Building on this insight, we postulated that adding electron-withdrawing (EWG) or electron-donating (EDG) groups on ynone **1a** could modify the electronic environment of the pyridinium core, potentially enhancing its fluorescence. Specifically, adding different combinations of EDG and EWG groups can narrow the energy gap between the highest occupied molecular orbital (HOMO) and the lowest unoccupied molecular orbital (LUMO), promoting fluorescence and inducing a red-shift in excitation wavelength. To test this theory, we synthesized five ynone derivatives (**1b**–**1f)** containing EWG and EDG groups using the Sonogashira cross-coupling reaction (Figure S5).^39^ We then treated these derivatives (**1b**–**1f**) with butylamine under ACA reaction conditions to yield the corresponding pyridinium products (**4b**–**4f**), with yields ranging from 17 to 29% (Figure 3A). The formation of all pyridinium products (**4b**–**4f**) was confirmed by ^1^H and ^13^C NMR spectroscopy (Figure S6).

Next, we evaluated the fluorescence properties of all the modified structures (**4b**–**4f**). Based on the UV–vis absorbance, we observed red shifts in the excitation wavelength (λ_max,ex_) of pyridiniums **4c**, **4d**, and **4f** (Figure S7). For pyridinium **4c** (λ_max,ex_ = ∼340 nm), a 40 nm red-shift was observed, while pyridiniums **4d** and **4f** displayed dual excitation wavelengths (λ_ex_ = ∼330 and ∼360 nm). This dual excitation wavelength is better visualized in ethanol and is characteristic of molecules that exist in multiple conformations or protonation states.^40^ The literature published on Katritzky salts, demonstrates that a single crystal of a sterically hindered 2,4,6-subsutituted pyridinium can exist as two conformations with twisted structures containing large torsion angles.^41^ Based on this report, we predict that our 2,3,4,6-substituted pyridiniums can exist as spatial isomers with highly twisted substituents, explaining their dual excitation as well as their NMR and HPLC peak splitting. In addition to inducing a red-shift in excitation wavelength, the addition of anisole groups to the pyridinium core promoted turn-on fluorescence for analogs **4c**–**4f** (Figure 3B). In contrast, the addition of trifluoromethylbenzene (**4b** and **4d**) did not improve the optical properties of the pyridiniums compared to their counterparts (**4a** and **4c**). We also observed significant Stokes shifts for **4d**, **4e**, and **4f** (λ_358_(**4f**) = 530 nm; Stokes shift ≈ 170 nm), which are crucial for detection sensitivity in fluorescence and cellular imaging applications.^42^ Overall, pyridiniums **4e** and **4f** displayed enhanced optical properties and were subjected to quantum yield measurements, revealing quantum yields of 0.027 and 0.031, respectively.

To understand the effect of EDG and EWG on the fluorescence of the pyridinium core, we performed DFT calculations to determine the HOMO–LUMO energy gaps of **4b** and **4e**. Pyridinium **4e** showed a significantly smaller HOMO–LUMO gap than **4b,** supporting the observed fluorescence (Figure 3C).

For cellular imaging studies, we screened varying excitation wavelengths (λ_ex_) and observed that **4f** showed higher fluorescence than pyridinium **4e** at ∼380 nm (Figure S8). Based on our comprehensive screening, including evaluation of excitation wavelength (λ_ex_) and quantum yield (φ), probe **4f** emerged as a promising candidate for further studies involving the LSF of peptides in cellular imaging applications.

### Chemoselectivity of ACA Reaction

Given the pronounced electrophilicity of ynone, we hypothesized its potential reactivity with other nucleophilic amino acids. To explore the specificity of lysine in generating the pyridinium product, we subjected peptides containing varying reactive amino acids, such as AcWAF, AcSMARW, AcDANRW and GARW, and AcXARW (X = Tyr, His, and Cys), to ynone **1f** under optimized reaction conditions (1:1 Buffer (pH 8)/MeCN, 80 °C, 15 h). As anticipated, the reactions yielded monoalkylated products with the N-terminus (90%), Tyr (52%), and His (51%). Monoalkylated Cys proceeded through elimination to yield dehydroalanine (Dha, > 99%) (Figures 4A,B and S9). However, the formation of the pyridinium product was unique to lysine, highlighting its specificity. This property enables further exploration of conditions capable of reversing monoalkylation of other amino acids in the presence of the pyridinium product on lysine. To reverse monoalkylated Tyr and His, we explored a nucleophilic substitution reaction with butylamine and subjected modified peptides, AcH­(Alk)­ARW, AcY­(Alk)­ARW, and AcLILK­(Py)­PF, to varying equivalents of the amine. We successfully reversed monoalkylated Tyr, Y­(Alk), and His, H­(Alk), with 30 equiv of butylamine. The lysine pyridinium, K­(Py), proved to be stable in these reversing conditions, yielding 93% conversion. (Figures 4C and S10).

**Figure 4:**
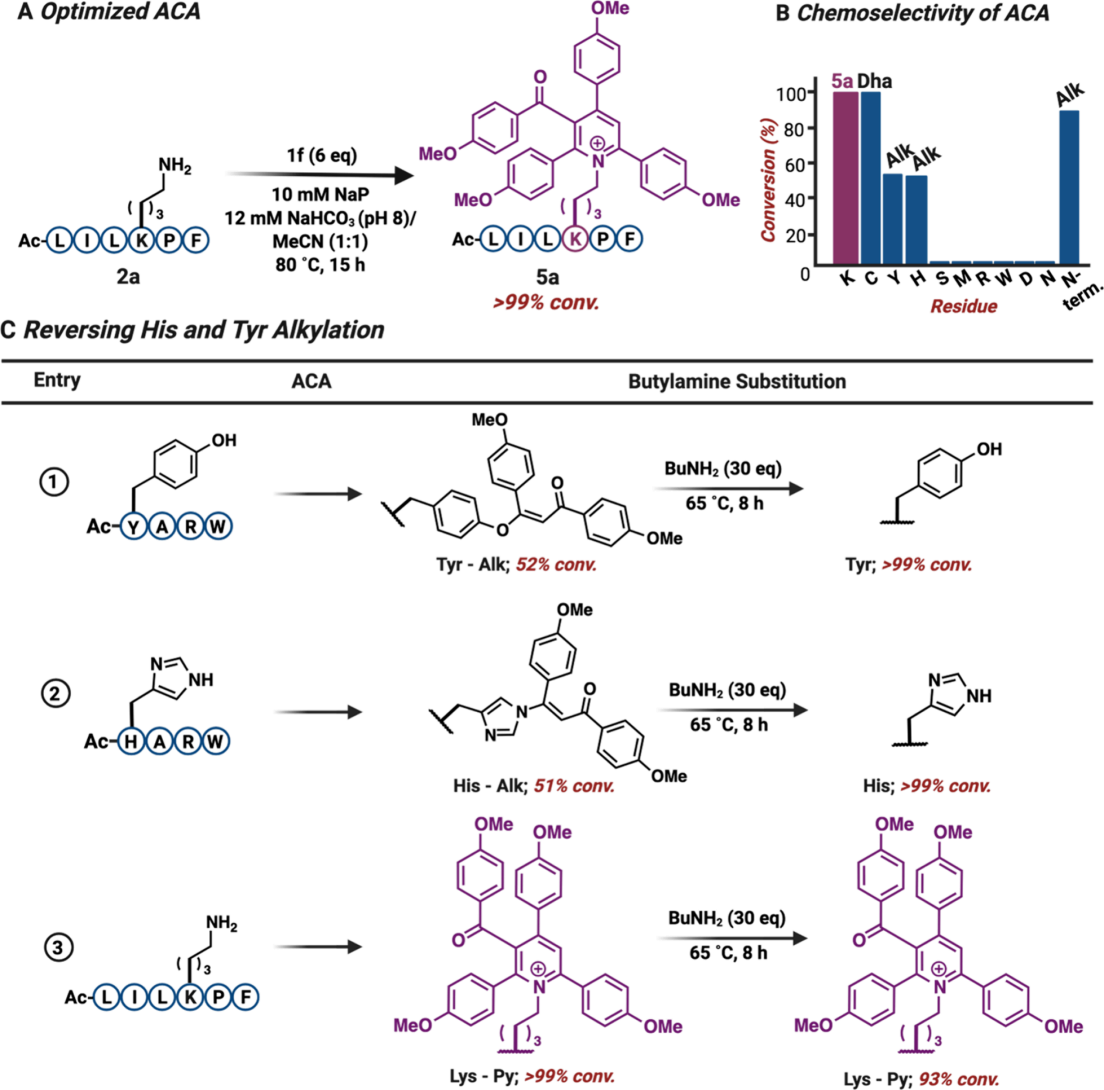
Exploration of ACA chemistry for late-stage functionalization of peptides. (A) Optimized ACA reaction conditions for efficient conversion of lysine to tetrasubstituted pyridiniums. (B) Chemoselectivity of ACA reaction showcasing the formation of stable pyridinium adduct with lysine and monoalkylation product with Tyr, His, and Cys. (C) His and Tyr monoalkylation was successfully reversed in the presence of butylamine (30 equiv), at 65 °C. Nucleophilic substitution reaction was performed in one pot, following ACA. Lysine pyridinium proved to be stable under these reversing conditions. Percent conversion was determined by HPLC and LC-MS. Created with BioRender.com.

### Scope of ACA for LSF of Bioactive Peptides

To evaluate the peptide scope of the ACA method, we installed the pyridinium DLC on various unprotected linear bioactive peptides of diverse sizes and amino acid compositions. Utilizing Fmoc-solid-phase peptide synthesis, we generated several bioactive linear peptides, including AcLILKPF **2a**, AcLDKVNR **2b**, AcVFKRN **2c**, AcPVNFKFLSH **2d**, and AcYLDKVLTQ **2e**, some exhibiting anti-inflammatory, hemopressin, and antihypertensive properties. These peptides were subjected to the optimized conditions with ynone **1f**. For peptides containing less reactive residues **2a**–**2c**, lysine was successfully converted to pyridinium **5a**–**5c** with high conversions ranging from 80 to 99%, and without generating any byproducts involving other reactive amino acids, including Arg, Asp, and Asn (Figures 5A and S11). For complex, longer peptides **2d**–**2e**, lower conversions were achieved (**5d**–**5e**, 44 and 71%), but no His- or Tyr-alkylated side-products were observed after one-pot butylamine addition, reversing the His and Tyr monoalkylated product (Figures 5A and S12). Notably, ACA-pyridinium peptide **5e** was synthesized and purified to give a 47% isolated yield (Figure S13). These examples highlight the exceptional precision of our approach in modifying lysine to the pyridinium core, rendering it an attractive approach for installing a fluorescent DLC on peptides.

**Figure 5:**
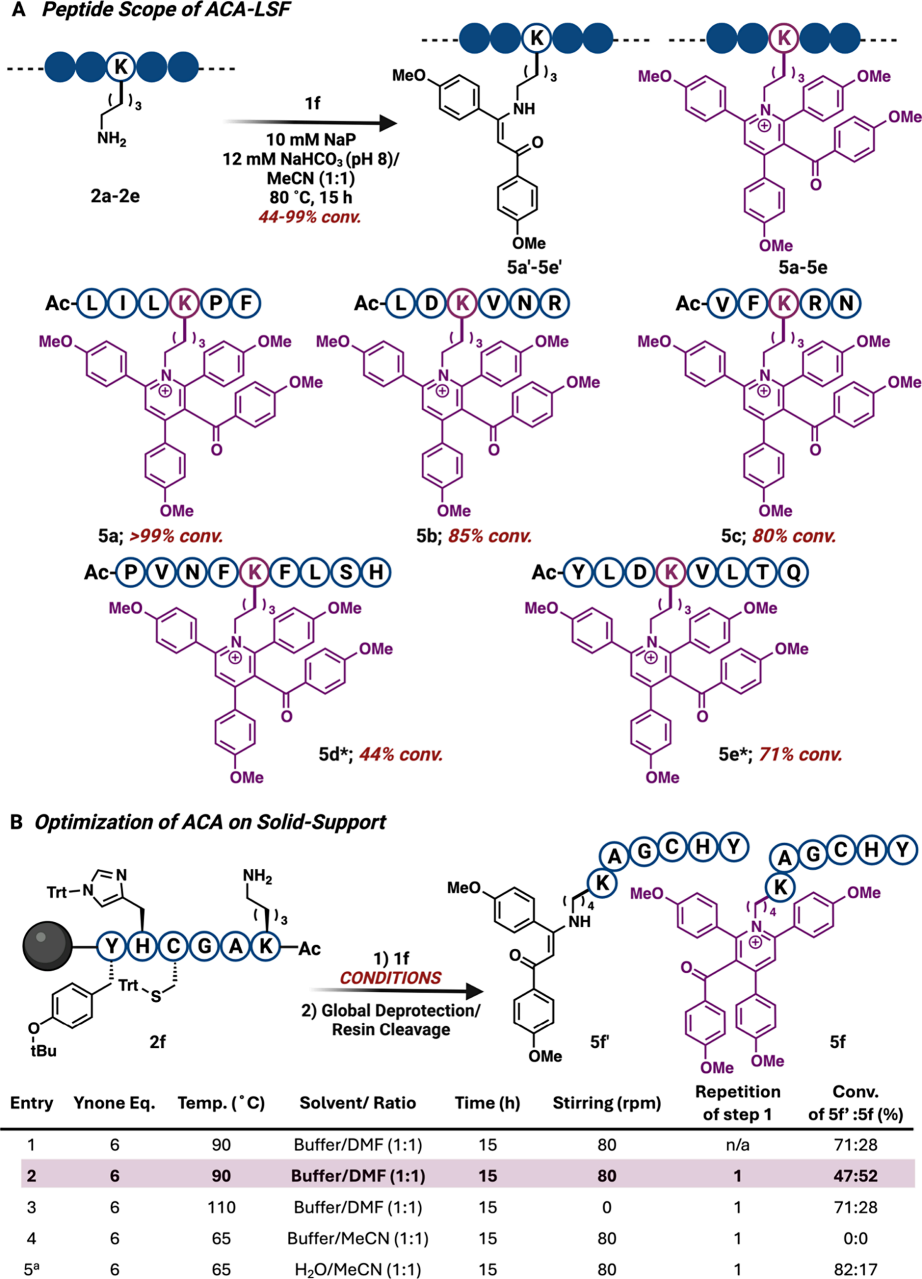
Substrate scope of ACA reaction for peptide modification both in solution and on solid support. (A) Substrate scope of ACA reaction for selective modification of lysine to pyridinium in varying peptides of different lengths and amino acid compositions. *Butylamine (30 equiv) was used in one pot to reverse alkylation of His and Tyr. (B) Late-stage functionalization of lysine peptides on solid support with other reactive amino acids. Percent conversion was determined by HPLC and LC-MS. Created with BioRender.com.

### Late-Stage Functionalization of Peptides on Solid Support

Since we observed irreversible modification of Cys residues to Dha, we proceeded to install the ACA-DLC on a solid support by selectively deprotecting Lys­(Mtt). We optimized the reaction on Fmoc–PAL-PEG resin using peptide **2f** KAGCHY, containing protected reactive amino acids, by subjecting it to different reaction conditions (solvent, temperature, and stirring speed) (Figures 5B and S14). The maximum yield for the desired pyridinium **5f** was obtained by using 6 equiv of ynone **1f** at 90 °C, over 15 h. Notably, we did not observe any modification of Cys or any other reactive residue under the reaction conditions.

### Application of ACA in Developing Mitochondria-Targeting Peptides for Live Cell Imaging

Given that this transformation generates a delocalized lipophilic pyridinium from lysine, we anticipated that these molecules might localize in mitochondria due to the negative membrane potential, which could enhance their ability to image and deliver peptides to mitochondria. To evaluate the potential of butylpyridinium **4f** as a mitochondria-targeting DLC in live cells, we coincubated **4f** (100 nM) and MitoTracker Red (100 nM) with breast cancer epithelial cell lines (T-47D) for 1 h, and we compared the fluorescence images taken at the compounds’ optimal excitation wavelengths.

Flow cytometry analysis revealed no increase in cell death compared with the naive control at the tested concentration (Figure S15). ACA-DLC **4f** was imaged in the Furan and DAPI channels (excitation at 380 and 405 nm), while MitoTracker Red was imaged in the near-IR TRITC channel (excitation at 566 nm). The merged images, taken across all three channels, clearly showed that both molecules were localized to the mitochondria at a concentration of 100 nM (Figure S16). The fluorescence intensity of ACA-DLC **4f** and MitoTracker Red in the mitochondria was quantified using pixel intensity analysis, indicating that **4f** exhibited fluorescence comparable to that of MitoTracker Red (Figure S17).

Encouraged by these results, we proceeded to assess the mitochondria-targeting ability of ACA-DLC-peptide **5b**. A bioactive peptide **2b**, which lacks the pyridinium core, was used as a negative control. We incubated ACA-DLC-peptide **5b** (100 nM) with T-47D cells for 45 min, along with MitoTracker Red, and assessed cellular localization through fluorescence imaging. After being washed, the cells were imaged using TIRF microscopy (Figure 6A). The merged images clearly showed the localization of ACA-DLC-peptide **5b** in the mitochondria (Figure 6B). No fluorescence was observed with control peptide **2b**. The fluorescence intensity of ACA-DLC-peptide **5b** and MitoTracker Red in the mitochondria was quantified using pixel intensity analysis (Figure 6C). Notably, the fluorescence intensity of ACA-DLC-peptide **5b** in the mitochondria was comparable to that of MitoTracker Red, demonstrating the ability of the pyridinium moiety to fully shuttle the peptide into the mitochondria.

**Figure 6:**
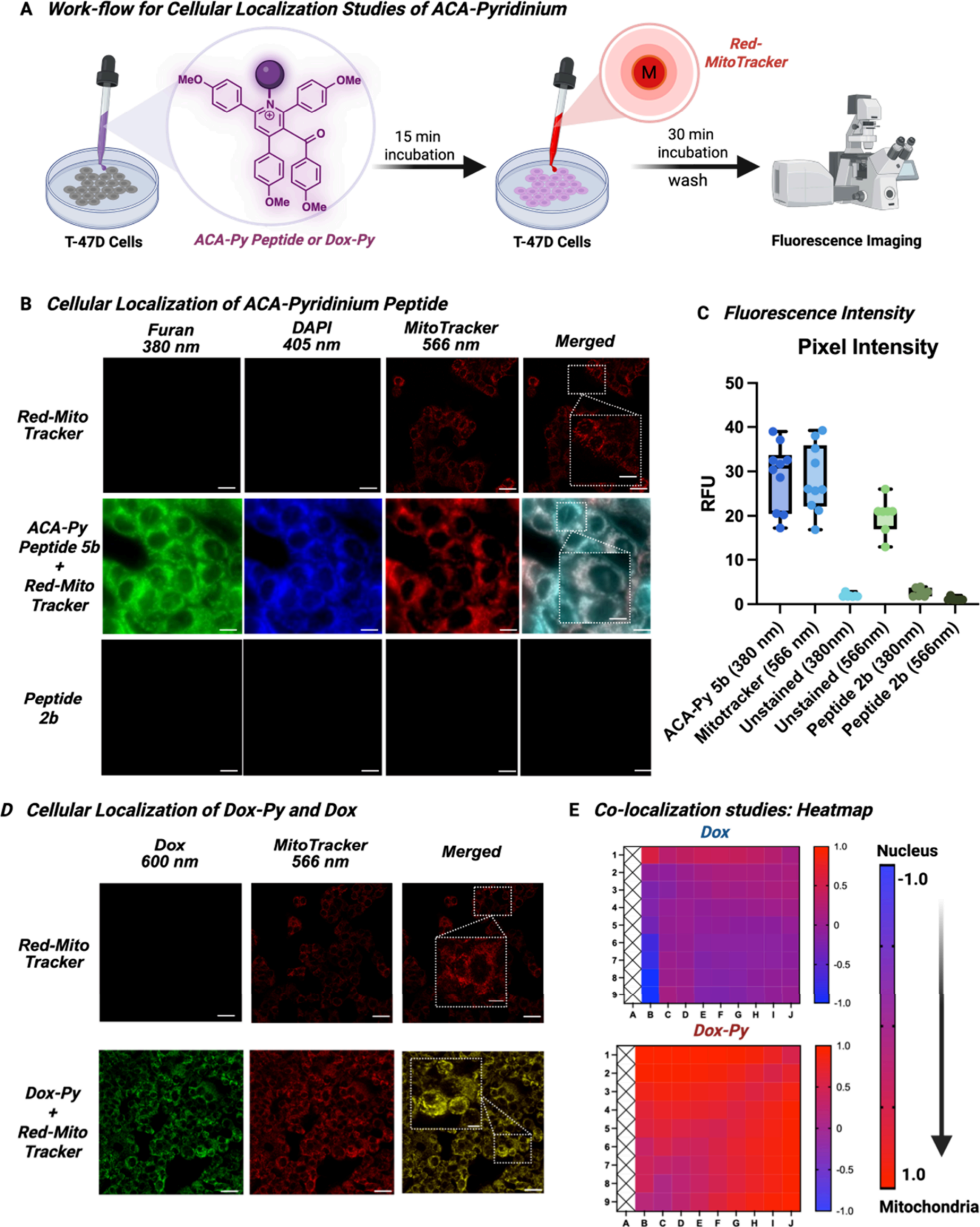
Exploration of ACA-pyridinium-modified peptide as potential fluorescent mitochondria-targeting probes. (A) Cellular imaging workflow of ACA-Pyridiniums. (B) Lane 1 (top) - shows the T-47D cells treated with MitoTracker Red. Lane 2 (middle) shows T-47D cells treated with both MitoTracker Red and ACA-Py peptide **5b**. Lane 3 (bottom) shows T-47D cells treated with native peptide **2b**. (C) Fluorescence intensity calculations show that ACA-Py peptide **5b** (100 nM) exhibits similar fluorescence intensity as MitoTracker Red (100 nM) in mitochondria, as calculated by pixel intensity analysis. (D) Dox-Py (100 nM) were incubated with T-47D cells, alongside MitoTracker (100 nM). Lane 1 (top) shows the T-47D cells treated with MitoTracker. Lane 2 (bottom) shows T-47D cells treated with both MitoTracker Red and Dox-Py. E) Addition of pyridinium DLC to Dox fully shuttles it from the nucleus to mitochondria, as indicated by the heat map analysis of Dox and Dox-Py-treated T-47D cells. All experiments were done in triplicates. The average cell is 34 μm, as measured by ImageJ software. Created with BioRender.com.

Additionally, we evaluated the effect of peptide sequence, length, and charge on the mitochondrial localization of ACA-modified peptides. Cellular localization studies of ACA-DLC-pentapeptide **5c**, with an overall + ve charge, and ACA-DLC-octapeptide **5e**, with an overall -ve charge, were performed by coincubation of each peptide and MitoTracker Red with T-47D cells (Figure S18). As observed for ACA-DLC-peptide **5b**, fluorescence imaging analysis of ACA-DLC-peptides **5c** and **5e** displayed remarkable localization in the mitochondria, with Pearson’s coefficients of 0.849 (**5b**), 0.899 (**5c**), and 0.917 (**5e**). These results underscore the sensitivity of our ACA-LSF method in converting lysine into mitochondria-targeting fluorescent probes, not limited by peptide charge, sequence, and length. To test whether ACA functionalization affects bioactivity, we compared native peptides with the ACA-modified analogues in two orthogonal assays (Figures S19 and S20). First, a broth microdilution against *E. coli* showed identical discrete MIC values (0.125 mM) and virtually superimposable extrapolated MICs for the antibacterial parent peptide **2g** and ACA-modified peptide **5g** (0.069 ± 0.06 vs 0.072 ± 0.01 mM), indicating unchanged antibacterial potency (Figure S21). Second, Annexin V/propidiumiodide flow cytometry of T-47D cells treated with 10 μM of peptide, for 2 h, revealed statistically indistinguishable cell death rates (78 ± 3% for parent peptide **2a** vs 82 ± 5% for ACA-modified peptide **5a**, *P* > 0.05) (Figure S22). Both experiments suggest that peptides functionalized by the ACA retain their original functions. This LSF platform enables the direct analysis of functionalized peptides, providing valuable insights into cell permeability, cellular stability, delivery, and subcellular localization.

### Live Cell Compatibility and Imaging of Mitochondria-Targeting ACA-Doxorubicin

We explored whether our pyridinium compounds could facilitate the relocation of a molecule prone to localization in organelles other than the mitochondria. Doxorubicin (Dox) was selected due to its nuclear localization and intrinsic fluorescence, which facilitates cellular imaging studies.^43^ Moreover, this enables direct comparison of the colocalization of the parent drug with that of the ACA-modified pyridinium drug (Figures S23 and S24). As a control, we used unmodified Dox, which is known to localize in the nucleus of susceptible cell lines.^44^ We conducted cell death experiments (Figure S25) using our T-47D cell line and observed a 40% survival rate, confirming their susceptibility to DOX. This result further supports the colocalization of DOX in the nucleus, consistent with previous reports in the literature. At a concentration of 100 nM, ACA-modified Dox-Py exhibited a maximum colocalization with mitochondria, supporting its role as a mitochondrial shuttle (Figure 6D). The significant overlap of signals between MitoTracker Red and Dox-Py further validated our hypothesis. In contrast, unmodified Dox was primarily colocalized with the nucleus. Pearson’s coefficients confirmed these observations, with Dox-Py showing a value of 0.898, and Dox showing a value of 0.397 (where 1 indicates perfect colocalization and −1 indicates none).^45^ A heatmap, representing the colocalization of Dox and Dox-Py with MitoTracker Red, was generated from the brightest 10% of pixels (intended to reduce false positives which might result from background signal). The heatmap distinctly shows Dox-Py colocalizing with the mitochondria (red), while unmodified Dox colocalized with the nucleus (blue), further corroborating our findings (Figure 6E).^46^

## Conclusions

In conclusion, we have pioneered a late-stage functionalization (LSF) platform based on an addition-cyclization-aromatization reaction (ACA), which exclusively generates novel 2,3,4,6-substituted pyridinium cores with lysine on reaction with ynones. Remarkably, these pyridinium cores exhibit high fluorescence and mitochondria-targeting properties, marking a transformative breakthrough in the field of peptide late-stage functionalization for bioimaging. By meticulously adjusting the electronic properties of substituents on ynones, we accomplish a striking fluorescence enhancement of the pyridinium core. The uniqueness of our ACA approach is evident, as it is the first lysine LSF method that installs a fluorescent mitochondria-targeting DLC. The synthesized organelle-targeting fluorescent peptide displayed significant intracellular fluorescence and colocalization in mitochondria under live cell imaging conditions. The fluorescence patterns observed within mitochondria underscore the potential of our ACA-LSF method for imaging peptide drugs. This work not only highlights the versatility of ACA-LSF in constructing complex peptide structures but also underscores the practical utility of these fluorescent organelle-targeting peptides. The mitochondria-targeting ability of the ACA-DLC was further proven by showing full mitochondrial localization of pyridinium-conjugated doxorubicin (Dox-Py). The low cytotoxicity observed at the concentrations used for live cell imaging and the retention of biological viability further emphasize the potential translational impact of our strategy. Our research opens new frontiers in the field of peptide late-stage functionalization for organelle targeting and bioimaging, offering a promising avenue for the development of advanced tools with applications ranging from subcellular targeting to drug delivery and therapeutic monitoring.

## Methods

### Synthesis

#### Procedure for Synthesis of Ynone Library

To an oven-dried round-bottom flask (100 mL), equipped with a stir bar, CuI (0.08 mmol) and PdCl_2_(PPh_3_)_2_ (0.04 mmol) were added. The flask was then purged with nitrogen, and anhydrous THF (16 mL) was injected into the flask. Benzoyl chloride (9 mmol) and phenyl acetylene (10.8 mmol) substrates were then added to the reaction flask and allowed to stir for 30 min. Et_3_N (1.3 mL, 9 mmol) was added, and the reaction was left stirring overnight at room temperature.^3^ The reaction was analyzed by TLC. The solvent was removed, and the reaction mixture was dissolved in DCM. The crude reaction mixture was then washed with saturated ammonium chloride solution (×3) and brine (×3). The organic layer was dried over sodium sulfate, and the crude product was purified by silica gel column chromatography using ethyl acetate/hexane as eluent. The product eluted at 5% ethyl acetate/hexane (*R*_f_ = 0.5).

#### Procedure for Synthesis of Pyridiniums

To a 25 mL round-bottom flask (equipped with a stir bar), ynone derivative (0.58 mmol, 3 equiv), dissolved in 5 mL of MeCN, and *n*-butylamine (0.19 mmol, 1 equiv) were added. Potassium carbonate (0.38 mmol, 2 equiv), dissolved in 5 mL of deionized water, was then added. The reaction was run in an oil bath under reflux and heated to 90 °C. The reaction was left stirring for 24 h at 1590 rpm and analyzed by TLC. After 24 h, MeCN was evaporated, and DCM was added to the reaction mixture. The water layer was extracted with DCM (×3). The organic layer was dried over sodium sulfate, and the crude product was purified by neutral aluminum oxide column chromatography using ethyl acetate/methanol as eluent. The product eluted at 90% ethyl acetate/methanol (*R*_f_ = 0.2).

### Cell Culture

T-47D cells were cultured in RPMI supplemented with 10% (V/V) fetal bovine serum (FBS) and 1% (V/V) penicillin/streptomycin (100 μg/mL) and maintained in an incubator at 37 °C with a 5% CO_2_/air environment.

### Cellular Studies

#### Flow Cytometry

Cells were grown in 60 mm × 15 mm Nunclon dishes. Stock solutions of compounds were prepared in DMSO before being diluted to the final desired concentration in 4 mL of culture media. Cells were placed in the incubator for treatment for 2 h. Cells were then detached with trypsin and stained using Annexin V/PI, following the manufacturer’s protocol (BioLegend cat: 640928). Briefly, the cells were detached with trypsin, washed twice with PBS, and resuspended in 100 μL of cold AV binding buffer. Then, cells were stained with 10 μL of Pacific Blue Annexin V for 10 min, followed by the addition of 10 μL of propidium iodide solution for 10 min. After 400 μL of Annexin V binding buffer was added to each tube, cells were analyzed via flow cytometry within 1 h to quantify cell death utilizing a BD FACSymphony A3 Cell Analyzer. FlowJo software was used to analyze the cytometry data.

#### Colocalization Studies

Cells were plated in an IBIDI 8-well glass bottom chamber at a density of 25,000 cells per well in media and allowed to adhere overnight at 37 °C, 5% CO_2_. Fresh 10 mM stock solutions of probes were prepared in DMSO on the day of experimentation. Working solutions of all compounds in media were prepared on the day of experimentation. Culture media was removed from the wells, and 200 mL of 100 nM probe was added to the desired wells and placed in an incubator for 30 min. Media was removed from the wells, and 200 mL of 100 nM MitroTracker-Red FM (ThermoFisher, M22425) was added to the desired wells and placed in an incubator for 15 min. Staining media was removed, and cells were washed with 200 μL of PBS for 5 min (repeated 3 times). PBS was removed and replaced with 200 mL of fresh medium, followed by immediate imaging. Five images were captured for each well. Colocalization analysis for Pearson’s R (R) and Mander’s Colocalization Coefficient (MCC) was conducted using the EzColocalization Plugin for ImageJ.

#### Minimum Inhibitory Concentration (MIC)

The minimum inhibitory concentration (MIC) assay was performed by using the broth microdilution method. A single bacterial colony was transferred from a prepared Mueller-Hinton Agar (MHA) plate into 3 mL of Mueller-Hinton Broth (MHB) and incubated at 37 °C for approximately 18 h, and the optical density at 600 nm (OD600) was checked. If OD600 exceeded 0.1, the culture was diluted with fresh MHB to reach 0.1; if it was below 0.09, incubation continued for an additional 15–30 min. The test compound was dissolved in MHB at twice the maximum test concentration. A 96-well microplate was prepared by dispensing 50 μL of MHB into wells of columns 1–10, leaving column 11 empty, and adding 100 μL of MHB to column 12 as a sterility control. Subsequently, 100 μL of the compound solution was added to column 11, followed by two-fold serial dilutions across to column 2, discarding 50 μL from the last well. A 50 μL aliquot of the standardized bacterial suspension (adjusted to ∼5 × 10^5^ CFU/mL) was added to each well in columns 1–11. The plate was sealed, labeled, and incubated overnight at 37 °C. After incubation, the plate was cooled to room temperature for 15 min, and OD600 values were measured using the Cytation 5 plate reader. The MIC was determined either visually, as the lowest concentration showing no turbidity, or quantitatively using a modified Gompertz model fit to the OD data.

## Supplementary Material



## Notes

Detailed synthetic procedures, methods, chemical characterization data, synthesis and characterization, mechanistic studies, ACA reaction, UV–vis spectra, fluorescence and quantum yield calculations, flow cytometry and imaging analysis, fluorescent properties, and ^1^H, ^19^F, and ^13^C-NMR spectra (PDF)

## Abbreviations

LSF: late-stage functionalization
ACA: addition-cyclization-aromatization
DLC: delocalized lipophilic cation
EWG: electron-withdrawing group
EDG: electron-donating group
HOMO: highest occupied molecular orbital
LUMO: lowest unoccupied molecular orbital
Ac: acetylated
Dha: dehydroalanine
Dox: doxorubicin
