# Supplementary material for: Late-Stage Functionalization of Lysine to Organelle-Targeting Fluorescent Probes
Source: JACS Au. 2025 Aug 16;5(9):4309–20. doi: 10.1021/jacsau.5c00680 (PMC12483151; doi:10.1021/jacsau.5c00680)
Supplement: Supplementary file 1 [file au5c00680_si_002.pdf]

Supporting Information

Late-Stage Functionalization of Lysine to Organelle-Targeting Fluorescent Probes

Patricia Rodriguez,[‡1] Ankita Misra,[‡1] and Monika Raj[*1]

[1]Department of Chemistry, Emory University, Atlanta, GA, United States, 30322

[‡]These authors contributed equally. *Corresponding author.

Table of Contents **Pages**

**I.**	General	2
**II.**	Materials	2
**III.**	Purification	2
**IV.**	Analytical Methods	2-3
**V.**	Fmoc Solid-Phase Peptide Synthesis	3
**VI.**	Experimental Section	3
**VII.**	Cell Culture Technique	3
**VIII.**	Computational Analysis Details	3-4
**IX.**	Colocalization studies – EzColocalization Details	4
**X.**	Figure 1: Detailed mechanism of Addition-Cyclization-Aromatization (ACA) reaction	4
**XI.**	Figure 2: Optimization of Addition-Cyclization-Aromatization (ACA) reaction on lysine	5-11
**XII.**	Figure 3: Synthesis and characterization of mono-alkylated butylamine	11
**XIII.**	Figure 4: Mechanistic studies – converting mono-alkylated intermediate to pyridinium	11-14
**XIV.**	Figure 5: Synthesis of ynone library	14-16
**XV.**	Figure 6: ACA reaction for synthesis of butyl pyridiniums	16-19
**XVI.**	Figure 7: UV-Vis spectra of butyl pyridiniums 4a-4f	19-20
**XVII.**	Figure 8: Fluorescence and quantum yield calculations of butyl pyridiniums	20-21
**XVIII.**	Figure 9: Chemoselectivity of ACA reaction	21-29
**XIX.**	Figure 10: Reversing Tyr and His alkylation	29-33
**XX.**	Figure 11: Synthesis of ACA-pyridinium peptides	33-36
**XXI.**	Figure 12: Synthesis of ACA-pyridinium peptides containing Tyr and His	36-40
**XXII.**	Figure 13: Synthesis, purification, and isolated yield of ACA-pyridinium peptide 5e	40
**XXIII.**	Figure 14: Solid-support synthesis of pyridinium peptides by ACA reaction	40-43
**XXIV.**	Figure 15. Flow cytometry analysis of butyl pyridinium 4f and peptide pyridinium 5b	43-44
**XXV.**	Figure 16: Imaging analysis for mitochondrial co-localization of butyl pyridinium 4f	44-45
**XXVI.**	Figure 17: Pixel Intensity of butyl pyridinium 4f	45
**XXVII.**	Figure 18: Imaging analysis for mitochondrial co-localization of peptide pyridinium 5b, 5c, and 5e	45-47
**XXVIII.**	Figure 19: Synthesis and purification of antibacterial ACA-Py peptide 5g	47-48
**XXIX.**	Figure 20: Synthesis and purification of ACA-Py peptide 5a	48-49
**XXX.**	Figure 21. Antibacterial potency of ACA-Py peptide 5g (MIC assay, *E. coli*)	49-50
**XXXI.**	Figure 22. Mammalian cytotoxicity of ACA-Py peptide 5a (Annexin V / PI flow cytometry)	50-51
**XXXII.**	Figure 23: Synthesis of Doxorubicin pyridinium (DOX-Py)	51-52
**XXXIII.**	Figure 24: Fluorescent properties of DOX-Py	52
**XXXIV.**	Figure 25: Flow cytometry analysis of Doxorubicin and DOX-Py	53
**XXXV.**	Figure 26: ^{1}H, ^{19}F and ^{13}C-NMR spectra of synthesized compounds	54-69
**XXXVI.**	References	69

I. General. All commercial materials (Sigma-Aldrich, Alfa Aeser, Ambeed, etc.) were used without further purification. All solvents were reagent or HPLC (Fisher) grade. All reactions for substituted and unsubstituted ynones were performed using anhydrous stabilized tetrahydrofuran (THF) and under N_2 atmosphere. The butyl pyridinium synthesis was performed under reflux (90-100 ˚C). Yields refer to chromatographically pure compounds. Percent conversions were obtained by comparing HPLC peak areas of products, intermediates, and starting materials. TLC (Silica gel 60 F_{254}), HPLC and MS were used to monitor reaction progress, and product elucidation was done using MS and NMR.

II. Materials. Fmoc-amino acids, Rink amide resin, Hydroxybenzotriazole (HOBt) and N,N′-diisopropylcarbodiimide (DIC) were obtained from CreoSalus (Louisville, Kentucky). Piperidine and trifluoroacetic acid (TFA) were obtained from Alfa Aesar (Ward Hill, Massachusetts). *N,N*-dimethylformamide (DMF), dichloromethane (DCM), methanol (MeOH), acetonitrile (MeCN), Triisopropylsilan (TIPS) and potassium carbonate (K_2CO_3) were obtained from VWR (Radnor, Pennsylvania). N,N-Diisopropylethyamine (DiPEA) was obtained from Beantown Chemical (Hudson, New Hampshire). Bis(triphenylphosphine)palladium(II) dichloride ($PdCl_2(PPh_3)_2$) was obtained from AmBeed (Arlington Heights, Illinois). Anhydrous tetrahydrofuran (THF), copper(I) iodide (CuI), triethylamine (Et_3N), 1,3-dimethoxybenzene (1,3-DMB), sodium bicarbonate ($NaHCO_3$), beta-alanine and bovine serum albumin (BSA) were obtained from Sigma-Aldrich (St. Louis, Missouri). Doxorubicin hydrochloride was obtained from Combi-Blocks (San Diego, California). Red-MitoTracker™ and Fmoc-PAL-PEG-PS resin were obtained from Thermo Fisher Scientific (Waltham, Massachusetts). AV/PI stains (FITC and PacificBlue) were purchased from Biolegend (San Diego, California). Cell lines were obtained from the Spangle Lab at Winship Cancer Institute of Emory University School of Medicine.

III. Purification.

PREP-HPLC: Purification of peptide starting materials, peptide pyridiniums, beta-alanine pyridinium, and doxorubicin pyridinium was performed using an ACCQPrep HP150, preparative high performance liquid chromatography (PREP-HPLC), equipped with a Teledyne RediSep® C18 reverse phase column with a particle size of 5 μm. All separations involved a mobile phase of 0.1% formic acid in water (solvent A) and 0.1% formic acid in acetonitrile (solvent B). The PREP-HPLC method used a linear gradient of 5-60% B over 30 min, and an isocratic gradient of 98% B for 10 min, at RT with a flow rate of 1 mL min^{-1}. The eluent was monitored by absorbance at 220 nm.

Column Chromatography: All synthesized ynones were purified using silica gel, SiliaFlash® GE60 (Silicycle, 70-230 mesh) column chromatography. The butyl pyridiniums were purified using activated neutral aluminum oxide (Brockmann I, 58 Å pore size).

IV. Analytical Methods.

NMR: ^{1}H and ^{13}C spectra were acquired at 25 ˚C in $CDCl_3$ using a Bruker 400 or 800 MHz spectrometer. All ^{1}H NMR chemical shifts (δ) were referenced relative to the residual $CDCl_3$ peak at 7.28 ppm. ^{13}C NMR chemical shifts were referenced to $CDCl_3$ at 77.2 ppm. ^{13}C NMR spectra were acquired under proton decoupled. Spectra were processed using MestReNova ver. 12.0.4 software. NMR spectral data are reported as chemical shift (multiplicity, coupling constants (J), integration). Multiplicity is reported as follows: singlet (s), doublet (d), doublet of doublets (dd), doublet of doublet of doublets (ddd), doublet of triplets (td), triplet (t) and multiplet (m). Coupling constants (J) are reported in Hertz (Hz).

Analytical HPLC: Analytical HPLC chromatography (HPLC) was performed on an Agilent 1200 series HPLC equipped with a Phenomenex C18 reversed-phase column, with a 5 μm pore size. All separations involved mobile phase of 0.1% formic acid in water (solvent A) and 0.1% formic acid in acetonitrile (solvent B) run in linear gradients with a constant flow rate of 1 mL min^{-1}. The eluent was monitored with a detection wavelength of 220 nm. **HPLC METHOD:** Gradient: 2-80% B over 30 min, and 98% B for 5 min.

HRMS: High resolution MS data were acquired on Thermo Exactive Plus using a heated positive-ion electrospray (+ESI) source. The solution was infused at a rate of 10-25 μL $min\text{-}^{1}$ electrospray using 3.3 kV. The typical settings were Capillary temp 320 ˚C. S-lens RF level was between 30-80 with an AGC setting of 1 E^6. The maximum injection time was set to 50 ms. Spectra were taken at 140,000 resolutions at m/z 200 using Tune software and analyzed with Thermo's Freestyle software.

V. Fmoc Solid-Phase Peptide Synthesis (Fmoc-SPPS).[1] Peptides were synthesized using standard protocols. Peptides were synthesized manually on a 0.25 mmol scale using Rink amide or Fmoc-PAL-PEG-PS resin. Resin was swollen with DCM/DMF (4:1) for 1 h at RT. Fmoc was deprotected using 20% piperidine–DMF for 30 min, at RT, to obtain a deprotected resin. First of the sequenced Fmoc protected amino acid (1.25 mmol, 5 equiv.) was coupled using HOBt (1.25 mmol, 5 equiv.) and DIC (1.25 mmol, 5 equiv.) in DMF for 1 h at RT. Fmoc-protected amino acids (0.75 mmol, 3 equiv.) were sequentially coupled on the resin using HOBt (1.25 mmol, 5 equiv.) and DIC (1.25 mmol, 5 equiv.) in DMF for 1 h at RT. After every deprotection and coupling step, the resin was washed with DCM, DMF and MeOH (x4). Prior to cleaving from resin, the N-terminus of peptides was acetylated using 20% acetic anhydride–DMF for 1 h at RT. Peptides were cleaved from the resin using 6 mL of a cocktail consisting of 90:2.5:2.5:2.5:2.5 trifluoroacetic acid : water : triisopropylsilane (TIPS) : 1,3-dimethoxybenzene (1,3-DMB) : ethanedithiol (EDT) for 1.5 h. The resin was removed by filtration and the resulting solution was concentrated. Peptides were precipitated and centrifugated with cold diethyl ether (3 x 10 mL) to obtain the crude product. Crude peptides were dissolved in $MeCN:H_2O$ and purified by PREP-HPLC, using method reported purification section.

VI. Experimental Section. The buffer used for the synthesis of pyridinium on peptides is a 10 mM NaP/ 12 mM $NaHCO_3$ (pH 8). This buffer was made by first preparing 20 mL of 10 mM NaP buffer (188 μL of NaH_2PO_4 and 11.2 μL of Na_2HPO_4, pH 5.8), and adding 20.2 mg (0.24 mmol) of $NaHCO_3$.

VII. Cell Culture Technique. Cells were maintained at 37 ˚C and 5% CO_2. T-47D cells were cultured in RPMI supplemented with 10% (V/V) fetal bovine serum (FBS) and 1% (V/V) penicillin/streptomycin (100 μg/mL).

VIII. Computational Analysis Details. The calculations in this study utilized the Gaussian-16 software package. The geometry of all structures was optimized using the B3LYP-D3(BJ)/[6-31G(d,p)] level of theory, which combines the B3LYP density functional with Grimme's empirical dispersion-correction (D3) and Becke-Johnson (BJ) damping-correction. The split-valence 6-31G(d,p) basis sets were employed for all atoms. Frequency analyses were conducted at the same level as the geometry optimization to characterize the minimum structures and to include enthalpy and entropy corrections. The effects of the solvent were considered by incorporating bulk solvent effects using the SMD model, with water chosen as the solvent. The reported thermodynamic data were calculated at a temperature of 298.15 K and a pressure of 1 atm.

To comprehend the underlying mechanisms of fluorescence in 4e we investigated and compared the lowest energy conformations of 4b and 4e. Literature suggests that adding different combinations of EDG and EWG groups on a molecule, can reduce the energy gap between the highest occupied molecular orbital (HOMO) and the lowest unoccupied molecular orbital (LUMO), promoting fluorescence and inducing a red-shift in the excitation wavelength.[2] Experimental data showed that 4b did not display fluorescence, however, 4e did. To understand these result, we conducted Density Functional Theory (DFT) calculations. The lower-energy conformers of 4e and 4b were found to have ΔE values of 3.74265 eV and 4.34593 eV, respectively. The larger HOMO–LUMO gap of 4b reduces the probability of transitions, which accounts for its OFF-fluorescence compared to 4e.

IX. Colocalization studies – EzColocalization Details. Co-localization studies were done using *EzColocalization*. *EzColocalization* is an ImageJ Application Program Interface (APIs)-an open-source plugin, where individual cells can be highlighted and colocalization studies can be run across three channels. The metric matrix for TOS (linear scaling) in the heatmap quantifies colocalization. FT represents the top percentage of pixels by intensity within a channel (e.g. FT = 80% includes the brightest 80% of pixels).

X. Figure 1: Detailed mechanism of Addition-Cyclization-Aromatization (ACA) reaction.

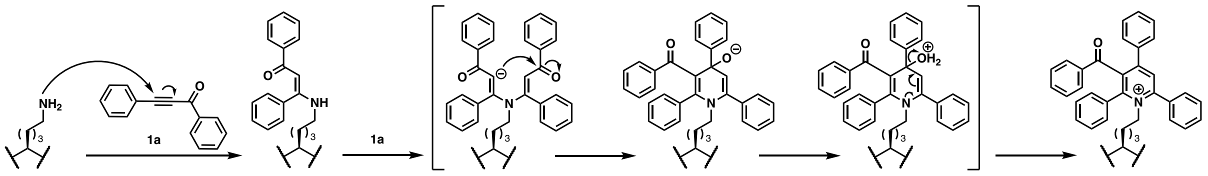

XI. Figure 2: Optimization of Addition-Cyclization-Aromatization (ACA) reaction on lysine.

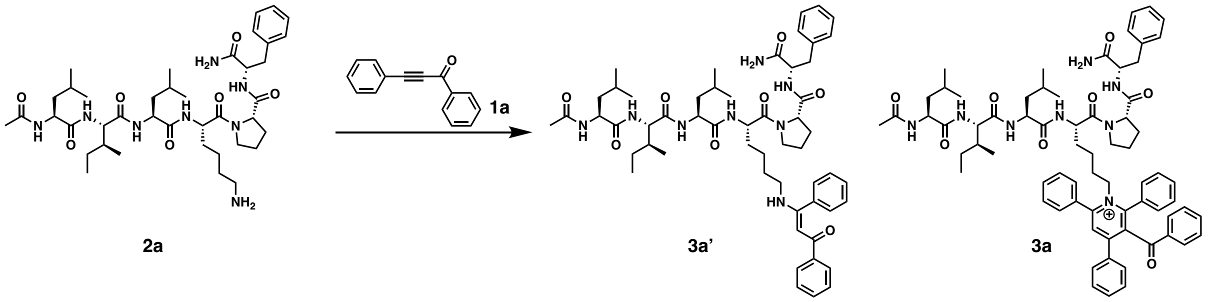

Entry	Ynone Eq.	Temp. (˚C)	Solvent/ Ratio	Time (h)	Conversion of 2a:3a':3a (%)
1	6	37	Buffer/MeCN (1:1)	15	24:55:20
2	9	37	Buffer/MeCN (1:1)	15	32:59:8
3	6	65	Buffer/MeCN (1:1)	15	0:18:81
4	9	65	Buffer/MeCN (1:1)	15	0:15:84
5	6	80	Buffer/MeCN (1:1)	15	0:4:95
6	9	80	Buffer/MeCN (1:1)	15	0:2:97
7	6	80	Buffer/MeCN (2:1)	15	0:3:96
8	6	80	Buffer/MeCN (1:2)	15	0:3:96
9[a]	6	80	H_2O/MeCN (1:1)	15	0:4:95
10[b]	6	80	DMF	15	0:>99:0
11	6	80	Buffer/MeCN (1:1)	1	0:63:36
12	6	80	Buffer/MeCN (1:1)	25	0:2:97
13	6	80	Buffer/MeCN (1:1)	5	0:27:72
14	6	80	Buffer/MeCN (1:1)	10	0:14:85
15	6	80	Buffer/MeCN (1:1)	15	0:4:95
16	6	80	Buffer/MeCN (1:1)	20	0:3:96

Reactions were run with K_2CO_3 (1 eq)[a] and DiPEA (1 eq)[b].

To 1 mg (1.3 µmol) of lysine peptide 2a, dissolved in 217 µL of solvent (6 mM), the appropriate amount of ynone was added. All reactions were run in a 2 mL glass vial, equipped with two stir bars. All of the reactions were run in an oil bath equipped with a thermometer and were left stirring, at 1600 rpm, for the appropriate time. For the reactions run in Buffer/MeCN, a pre-made 10 mM NaP/12 mM $NaHCO_3$ (pH 8) buffer was used. Samples were taken from the reaction mixture and injected into the HPLC and MS to monitor the reaction. The reaction mixture was analyzed by the HPLC method reported in the analytical method. Percent conversion was determined by calculating the area under the HPLC peaks of reaction mixture.

Lysine Peptide 2a. HRMS (+ESI): m/z 771.5128 (calcd $[M+H]^+$ = 771.5127), m/z 793.4938 (calcd $[M+Na]^+$ = 793.4952). (HPLC analysis at 220 nm). Retention time on HPLC: 11.816.

HPLC of Lysine Peptide 2a

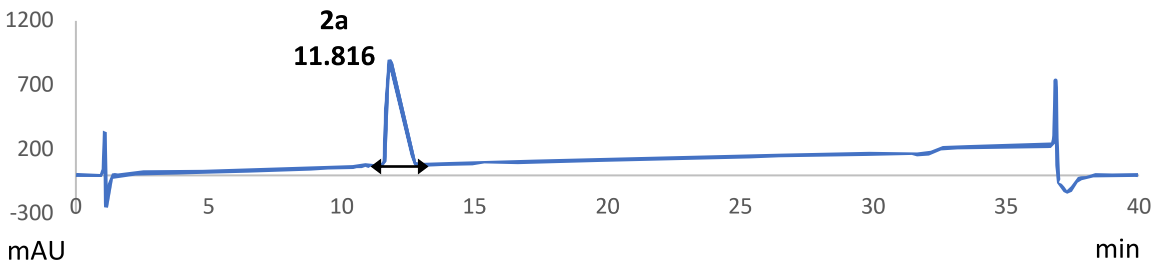

MS of Lysine Peptide 2a

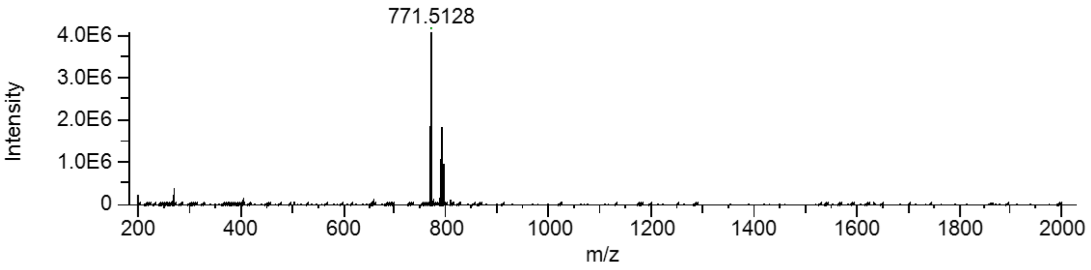

Alkylated Peptide 3a'. HRMS (+ESI): m/z 977.5858 (calcd $[M+H]^+$ = 977.5859), m/z 999.5671 (calcd $[M+Na]^+$ = 999.5684). (HPLC analysis at 220 nm). Retention time on HPLC: 23.653.

HPLC of Alkylated Peptide 3a'

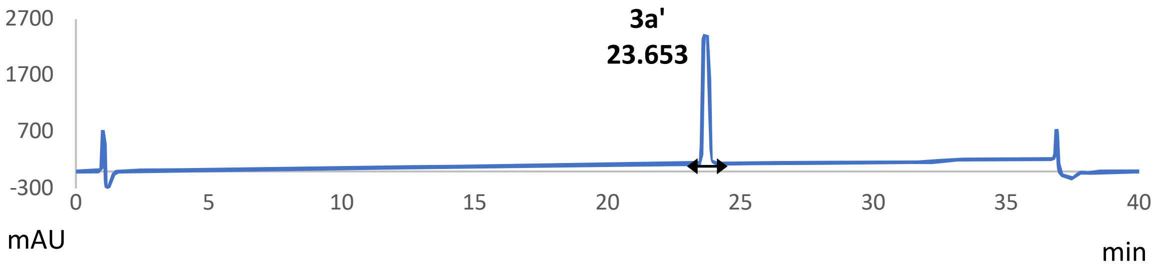

MS of Alkylated Peptide 3a'

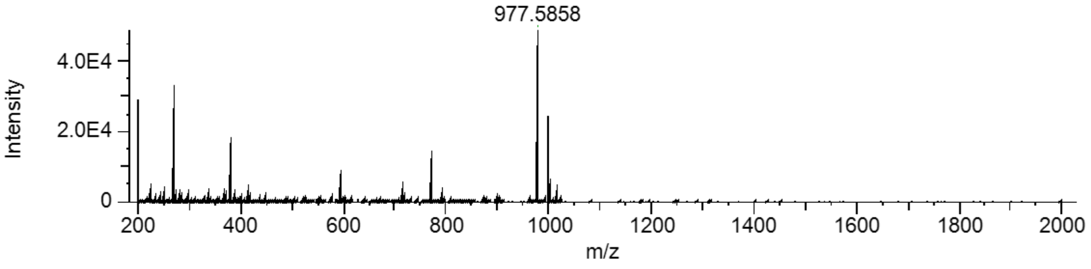

Pyridinium Peptide 3a. HRMS (+ESI): m/z 1165.6462 (calcd $[M]^+$ = 1165.6485). (HPLC analysis at 220 nm). Retention time on HPLC: 18.711.

HPLC of Pyridinium Peptide 3a

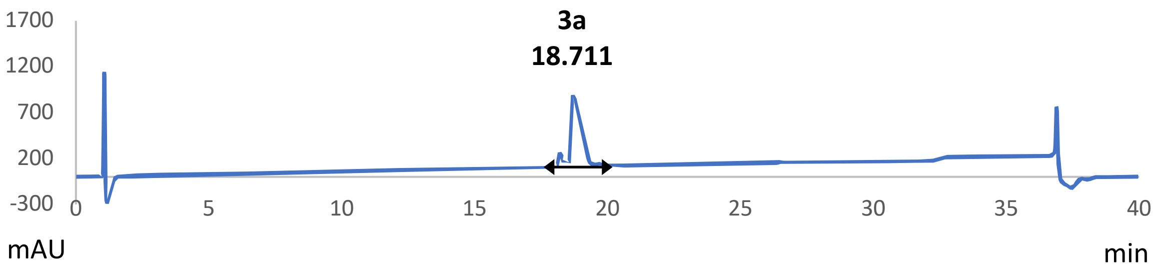

MS of Pyridinium Peptide 3a

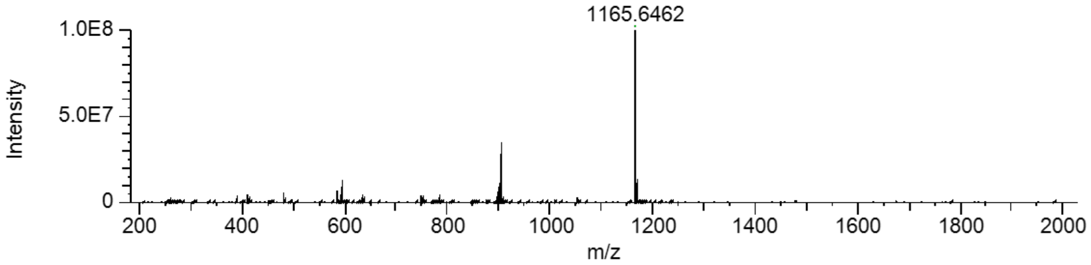

Entry 1 - Lysine Peptide 2a. HRMS (+ESI): m/z 771.5128 (calcd $[M+H]^+$ = 771.5127), m/z 793.4938 (calcd $[M+Na]^+$ = 793.4952). (HPLC analysis at 220 nm). Retention time on HPLC: 13.474.

Entry 1 - Alkylated Peptide 3a'. HRMS (+ESI): m/z 977.5858 (calcd $[M+H]^+$ = 977.5859), m/z 999.5671 (calcd $[M+Na]^+$ = 999.5684). (HPLC analysis at 220 nm). Retention time on HPLC: 24.667.

Entry 1 - Pyridinium Peptide 3a. HRMS (+ESI): m/z 1165.6462 (calcd $[M]^+$ = 1165.6485). (HPLC analysis at 220 nm). Retention time on HPLC: 20.053.

HPLC of Entry 1

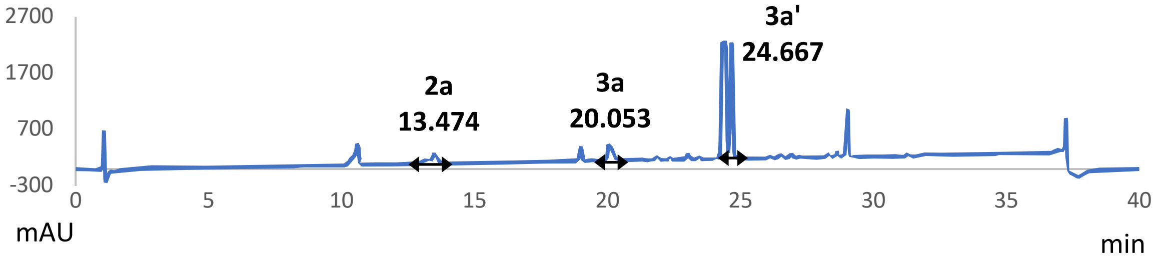

HPLC of Entry 2

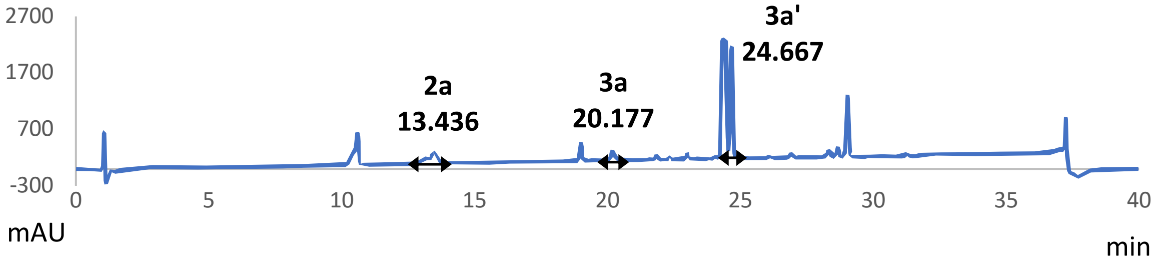

HPLC of Entry 3

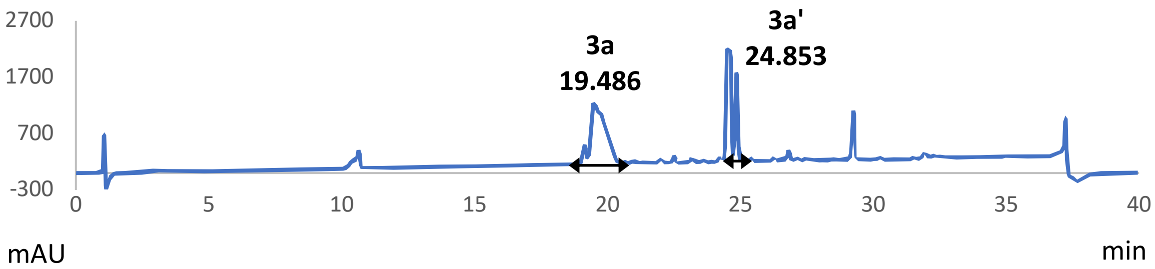

HPLC of Entry 4

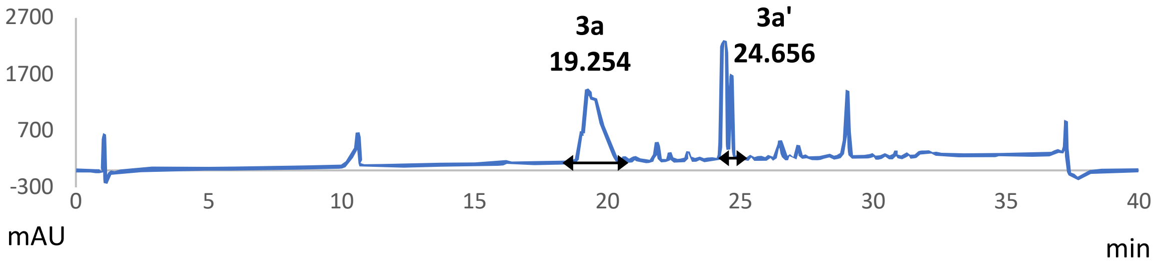

HPLC of Entry 5

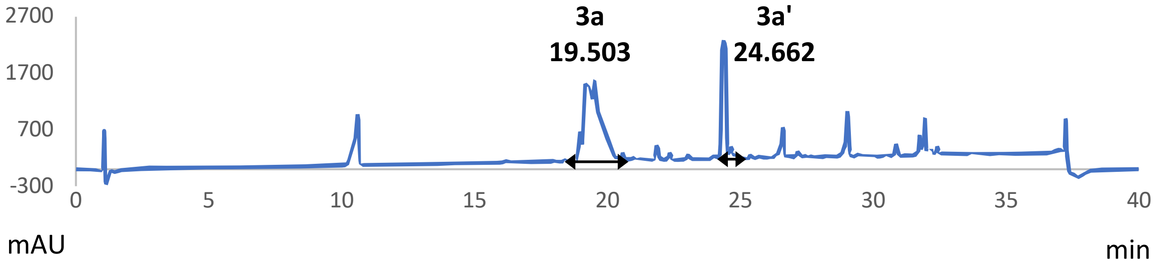

HPLC of Entry 6

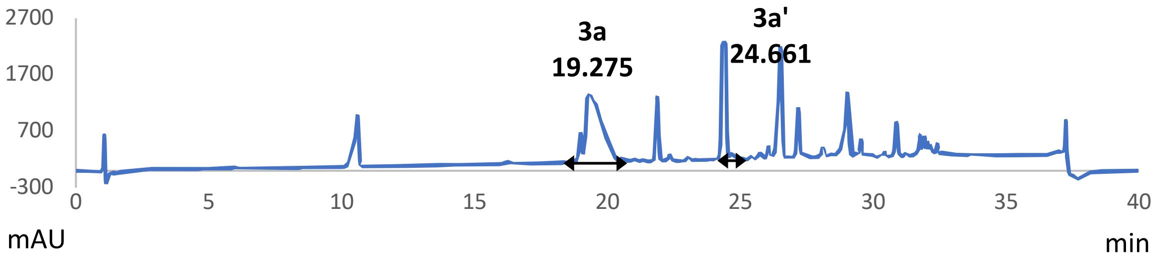

HPLC of Entry 7

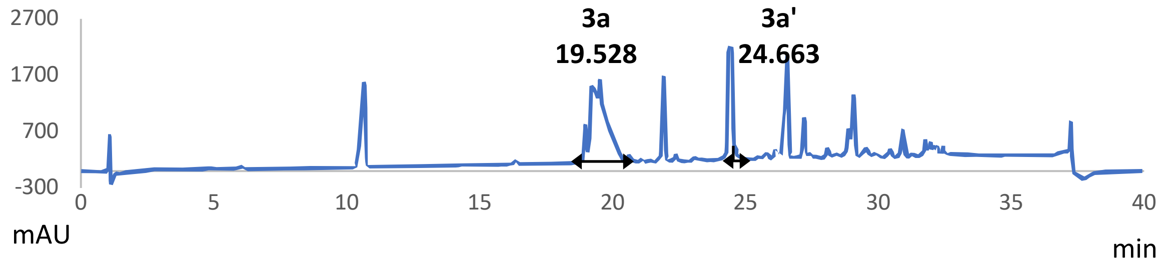

HPLC of Entry 8

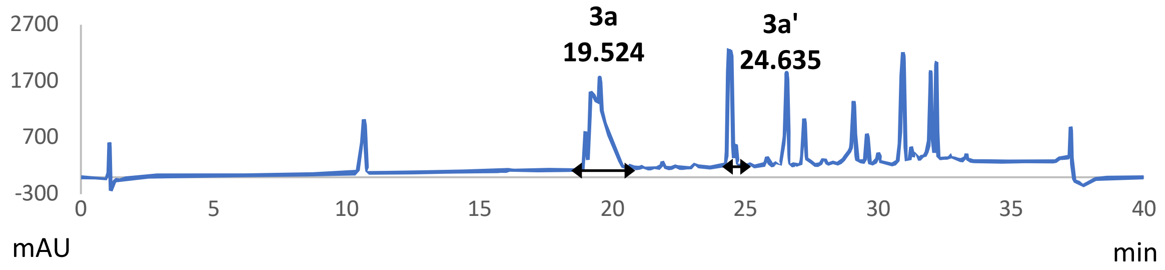

HPLC of Entry 9

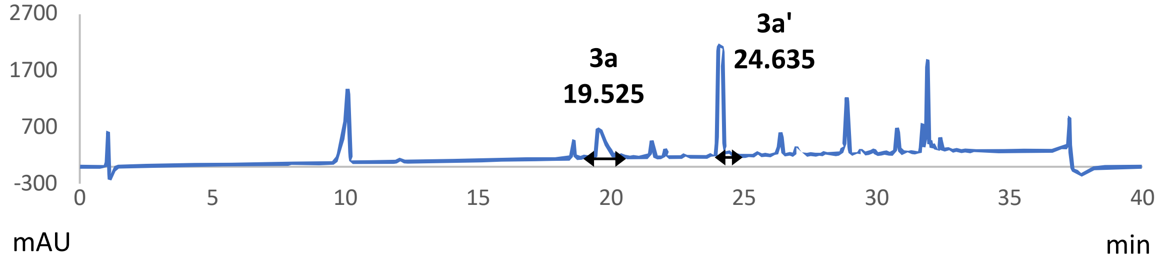

HPLC of Entry 10

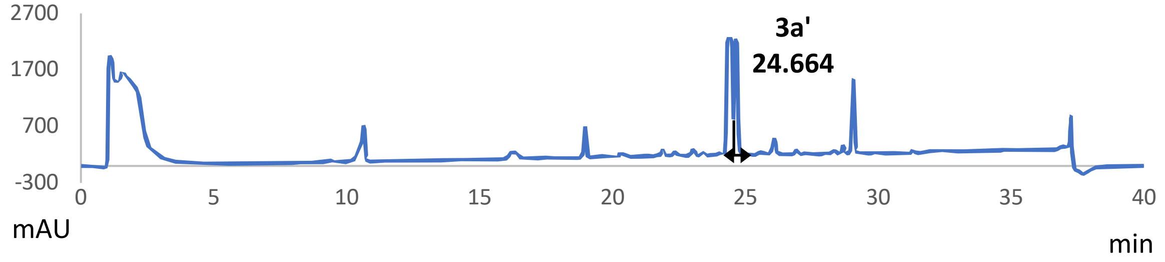

HPLC of Entry 11

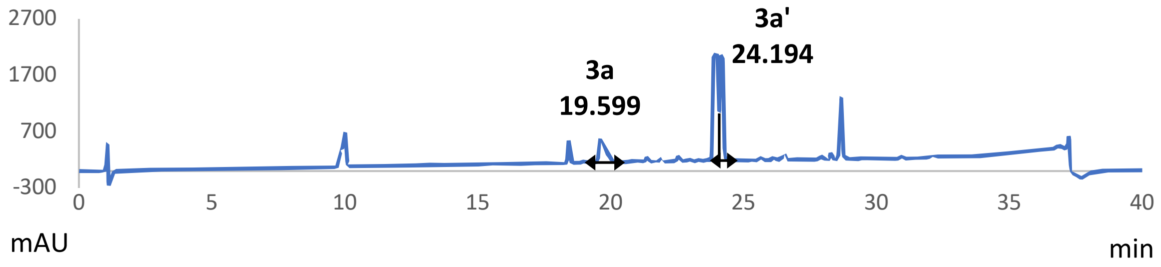

HPLC of Entry 12

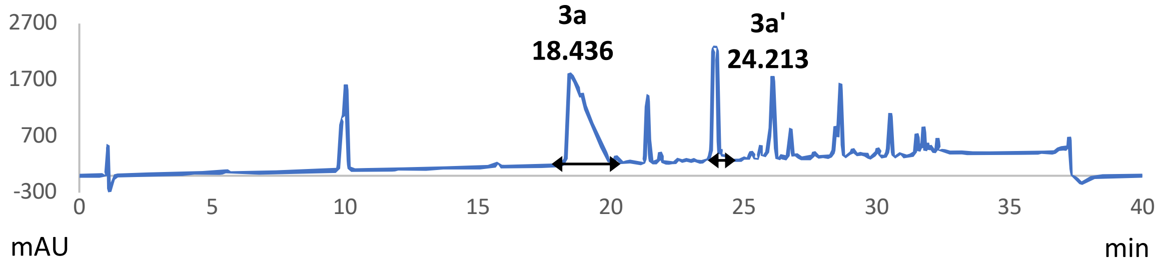

HPLC of Entry 13

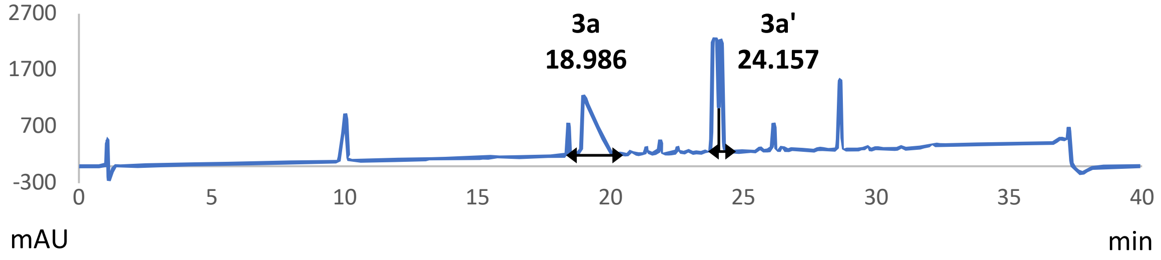

HPLC of Entry 14

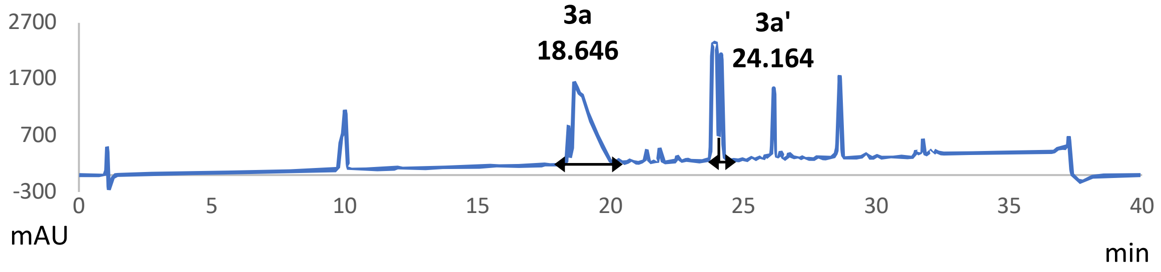

HPLC of Entry 15

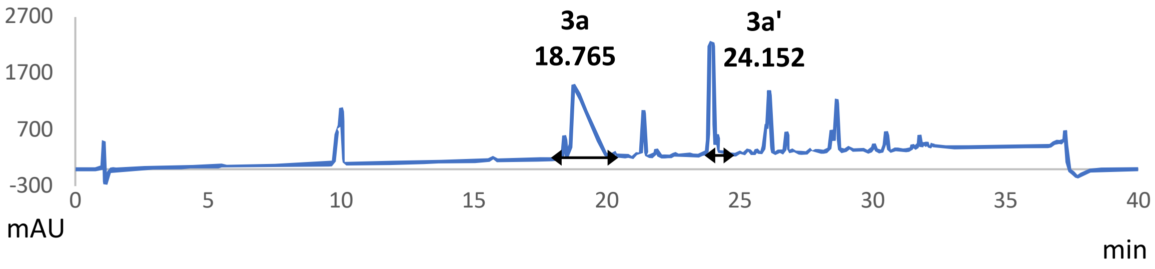

HPLC of Entry 16

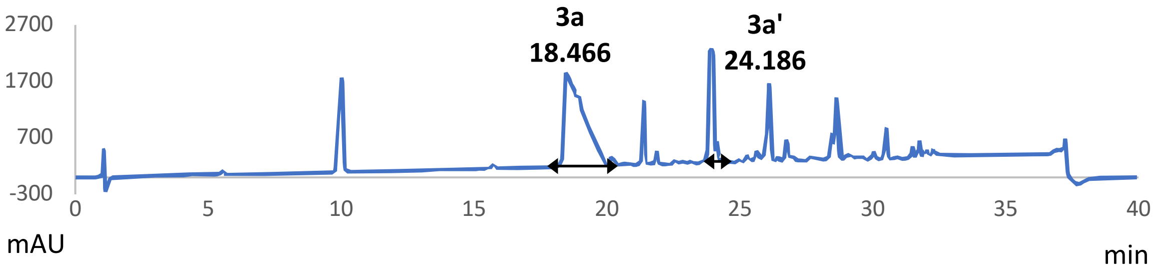

XII. Figure 3: Synthesis and characterization of mono-alkylated butylamine.

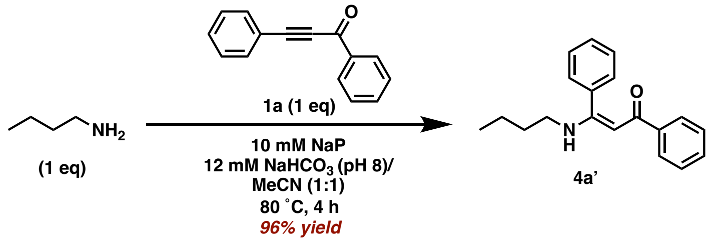

To a 25 mL round-bottom flask, ynone 1a (0.58 mmol, 1 eq), dissolved in 5 mL of MeCN, and n-butylamine (0.58 mmol, 1 eq), dissolved in 2 mL of 10 mM NaP/12 mM $NaHCO_3$ (pH 8), were added. Reaction was stirred for 4 hours under reflux. The MeCN was evaporated, and the reaction was diluted with water/EtOAc. The reaction was extracted with EtOAc (x3), and the combined organic layer was dried over sodium sulfate, filtered and concentrated. This compound was a yellow oil and was isolated to give 96% yield.

^{1}H NMR (400 MHz, $CDCl_3$): δ 11.43 (s, 1H), 7.89 (dd, J = 8.0, 1.7 Hz, 2H), 7.65 – 7.31 (m, 7H), 5.74 (s, 1H), 3.21 (td, J = 6.9, 6.0 Hz, 2H), 1.82 – 1.11 (m, 4H), 0.87 (t, J = 7.3 Hz, 3H)

^{13}C NMR (101 MHz, CDCl3): δ 188.65, 167.40, 140.83, 136.26, 131.05, 129.84, 128.94, 128.61, 128.14, 127.46, 93.64, 44.93, 33.26, 20.33, 14.11.

XIII. Figure 4: Mechanistic studies – converting mono-alkylated intermediate to pyridinium.

To a 2 mL vial, equipped with two stir bars, 1 mg (3.6 µmol) mono-alkylated butylamine 4a' was added and dissolved in 110 µL of MeCN and 110 µL of 10 mM NaP/12 mM $NaHCO_3$ (pH 8). Ynone 1a (18 µmol, 5 eq) was added and the reaction was stirred, at 1600 rpm, for 24 hours at 80 ˚C. The reaction was run in an oil bath, equipped with a thermometer. Aliquots of the reaction mixture were taken at several time intervals and injected into HPLC and MS to monitor the percent conversion to pyridinium product 4a**.** The reaction mixture was analyzed by the HPLC method reported in the analytical method. Percent conversion was determined by calculating the area under the HPLC peaks of reaction mixture.

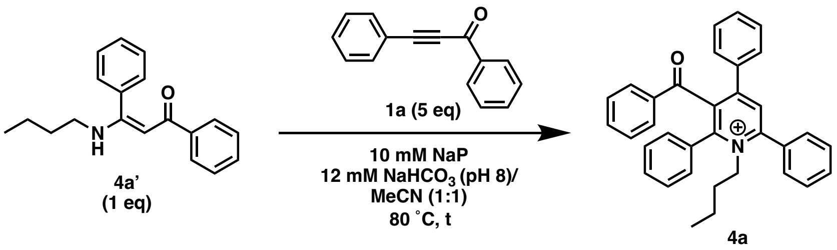

Entry	Time (h)	Conversion of 4a':4a (%)
1	1	95:5
2	3	64:36
3	5	36:64
4	7	21:79
5	9	17:83
6	15	10:90
7	24	3:97

mono-alkylated butylamine (4a'). (HPLC analysis at 220 nm). Retention time on HPLC: 26.

HPLC of 4a'

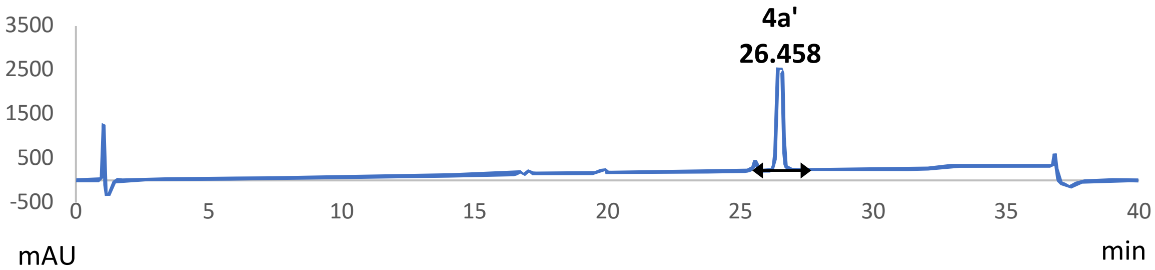

3-benzoyl-1-butyl-2,4,6-triphenylpyridin-1-ium (4a). HRMS (+ESI): m/z 468.2321 (calcd $[M]^+$ = 468.2322). (HPLC analysis at 220 nm). Retention time on HPLC: 18-20.

MS of 4a

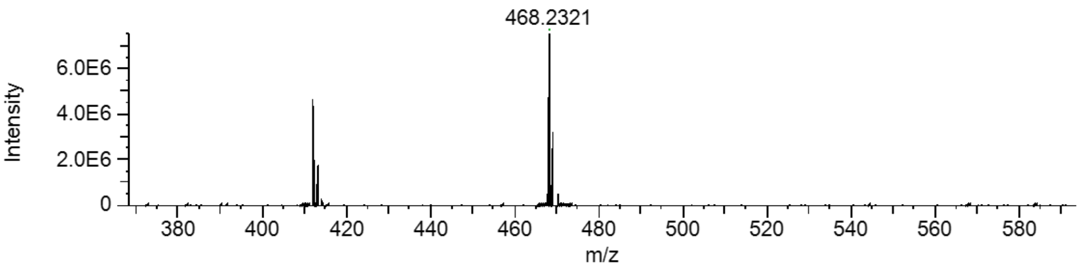

HPLC of Entry 1

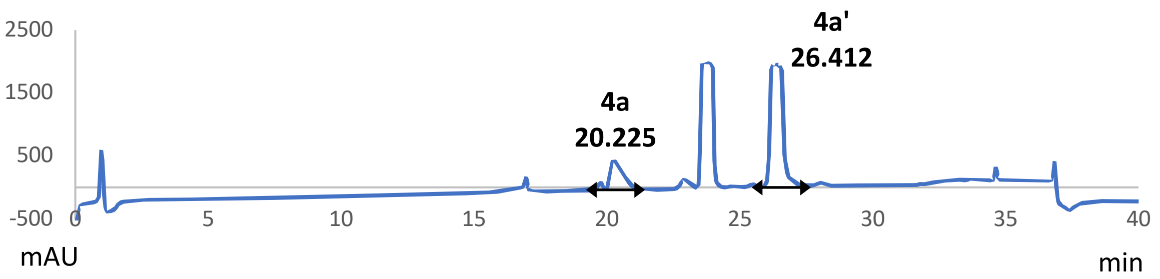

HPLC of Entry 2

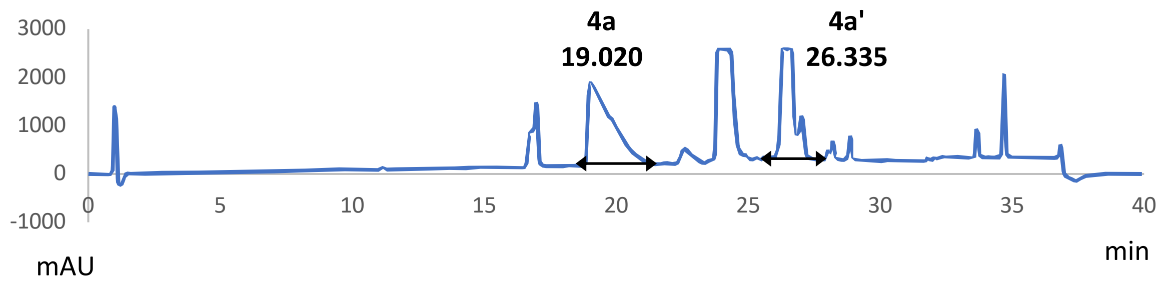

HPLC of Entry 3

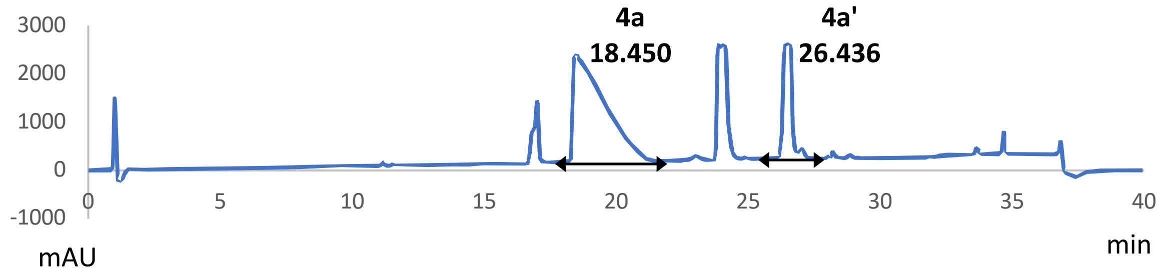

HPLC of Entry 4

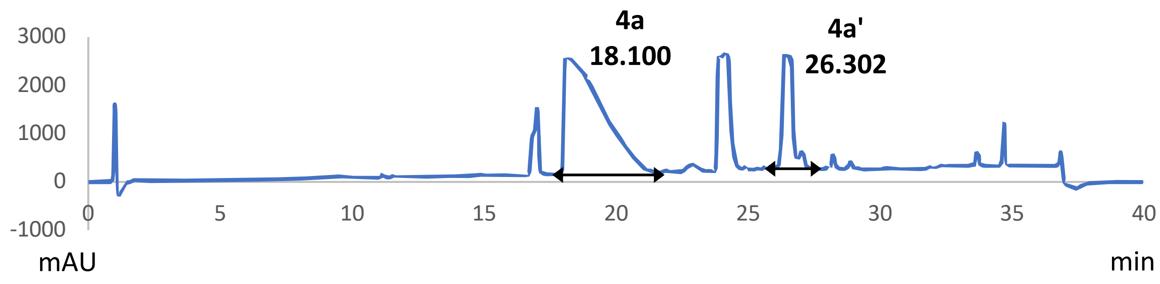

HPLC of Entry 5

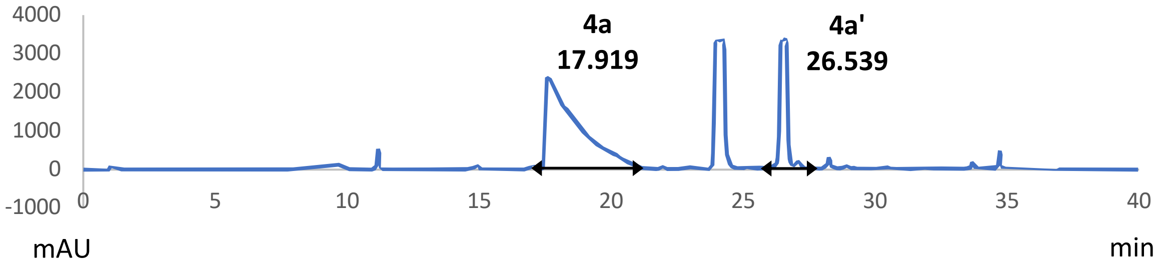

HPLC of Entry 6

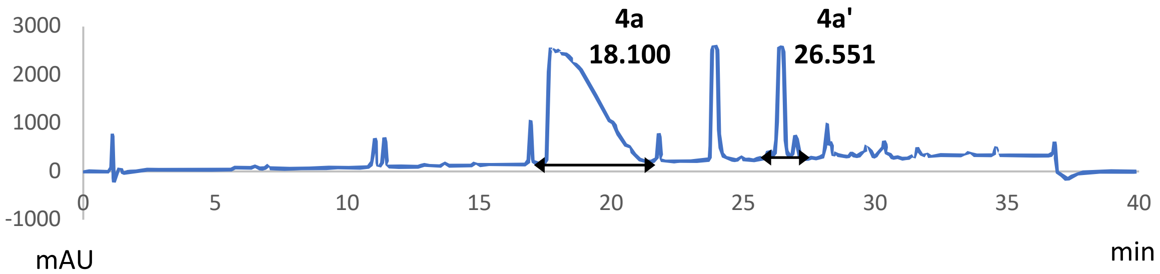

HPLC of Entry 7

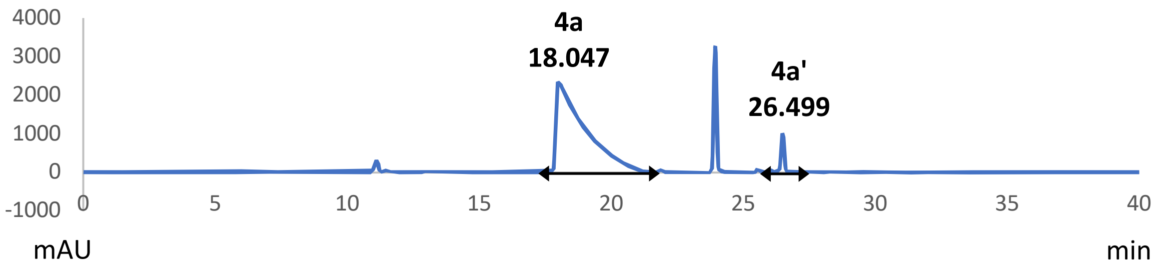

XIV. Figure 5: Synthesis of ynone library.

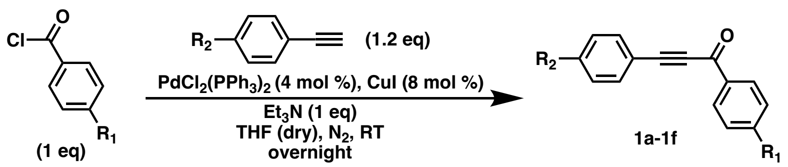

R_1 = H, CF_3, OMe; R_2 = H, OMe

To an oven-dried round bottom flask (100 mL), equipped with a stir bar, CuI (0.08 mmol) and $PdCl_2(PPh_3)_2$ (0.04 mmol) were added. The flask was then purged with nitrogen and anhydrous THF (16 mL) was injected into the flask. The benzoyl chloride (9 mmol) and phenyl acetylene (10.8 mmol) substrates were then added to the reaction flask, and were allowed to stir for 30 min. Et_3N (1.3 mL, 9 mmol) was added and the reaction was left stirring overnight, at room temperature.[3] The reaction was analyzed by TLC. The solvent was removed, and the reaction was dissolved in DCM. The crude reaction was then washed with saturated ammonium chloride solution (x3) and brine (x3). The organic layer was dried over sodium sulfate and the crude product was purified by silica gel column chromatography using ethyl acetate/hexane as eluent. The product eluted at 5% ethyl acetate/hexane (R_f = 0.5).

1,3-diphenylprop-2-yn-1-one (1a)

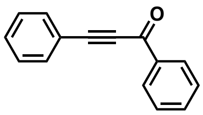

To an oven-dried round bottom flask (100 mL), 1-ethynyl-benzene and benzoyl chloride were added, following the general procedure. This compound was a yellow oil and was isolated to give 85% yield.

^{1}H NMR (400 MHz, CDCl$_3$) δ 8.26 – 8.21 (m, 2H), 7.73 – 7.67 (m, 2H), 7.67 – 7.60 (m, 1H), 7.56 – 7.47 (m, 3H), 7.46 – 7.39 (m, 2H). **^{13}C NMR (101 MHz, CDCl$_3$)** δ 178.18, 137.03, 134.27, 133.22, 130.94, 129.72, 128.83, 128.77, 120.27, 93.25, 87.03.

3-phenyl-1-(4-(trifluoromethyl)phenyl)prop-2-yn-1-one (1b)

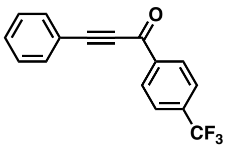

To an oven-dried round bottom flask (100 mL), 1-ethynyl-benzene and 4-(trifluoromethyl)benzoyl chloride was diluted with 10 mL of THF and added dropwise to the reaction mixture following the general procedure. This compound was a yellow powder and was isolated to give 56% yield.

^{1}H NMR (400 MHz, CDCl$_3$) δ 8.33 (d, J = 7.9 Hz, 2H), 7.80 (d, J = 8.2 Hz, 2H), 7.71 (d, J = 6.9 Hz, 2H), 7.53 – 7.45 (m, 3H).**^{13}C NMR (101 MHz, CDCl$_3$)** δ 125.86 (q, J = 3.7 Hz).**^{13}C NMR (101 MHz, CDCl$_3$)** δ 176.90, 139.54, 135.52, 135.20, 133.36, 131.37, 129.97, 128.96, 125.90, 125.86, 125.83, 119.83, 94.64, 86.74.**^{19}F NMR (376 MHz, CDCl$_3$)** δ -63.12.

3-(4-methoxyphenyl)-1-phenylprop-2-yn-1-one (1c)

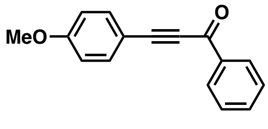

To an oven-dried round bottom (100 mL) flask, 1-ethynyl-4-methoxybenzene and benzoyl chloride were added, following the general procedure. This compound was a brown solid and was isolated to give 81 % yield.

^{1}H NMR (400 MHz, CDCl$_3$) δ 8.23 – 8.21 (m, 2H), 7.65 (d, J = 9.1 Hz, 2H), 7.63 – 7.60 (m, 1H), 7.52 (t, J = 7.5 Hz, 2H), 6.94 (d, J = 9.0 Hz, 2H), 3.86 (s, 3H). **^{13}C NMR (101 MHz, CDCl$_3$)** δ 177.10, 164.92, 133.38, 132.41, 131.02, 130.74, 129.08, 120.78, 114.32, 92.74, 87.35, 56.03.

3-(4-methoxyphenyl)-1-(4-(trifluoromethyl)phenyl)prop-2-yn-1-one (1d)

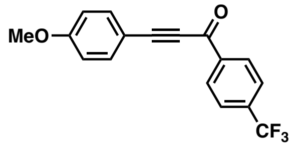

To an oven-dried round bottom flask (100 mL), 1-ethynyl-4-methoxybenzene and 4-(trifluoromethyl)benzoyl chloride were added, following the general procedure. This compound was a yellow solid and was isolated to give 71% yield.

^{1}H NMR (400 MHz, CDCl3) δ 8.31 (d, J = 8.0 Hz, 1H), 7.77 (d, J = 8.1 Hz, 2H), 7.65 (d, J = 8.9 Hz, 2H), 6.95 (d, J = 8.9 Hz, 2H), 3.87 (s, 3H).**^{13}C NMR** (101 MHz, CDCl$_3$) δ 176.81, 162.22, 139.72, 139.71, 135.49, 129.84, 125.83, 125.79, 125.75,125.71, 114.69, 111.52, 95.98, 86.89, 55.61.**^{19}F NMR** (376 MHz, CDCl$_3$) δ -63.08.

1-(4-methoxyphenyl)-3-phenylprop-2-yn-1-one (1e)

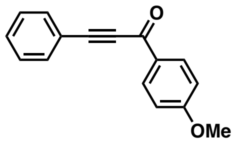

To an oven-dried round bottom flask (100 mL), 1-ethynyl-benzene and 4-methoxybenzoyl chloride were added, following the general procedure. This compound was a white powder and was isolated to give 84% yield.

^{1}H NMR (400 MHz, CDCl$_3$) δ 8.19 (d, J = 8.9 Hz, 2H), 7.67 (d, J = 7.0 Hz, 2H), 7.49 (d, 1H), 7.43 (d, 2H), 6.98 (d, J = 8.9 Hz, 2H), 3.89 (s, 3H). **^{13}C NMR (101 MHz, CDCl$_3$)** δ 177.10, 164.92, 133.38, 132.41, 131.02, 130.74, 129.08, 120.78, 114.32, 92.74, 87.35, 56.03.

1,3-bis(4-methoxyphenyl)prop-2-yn-1-one (1f)

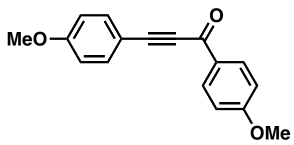

To an oven-dried round bottom flask (100 mL), 1-ethynyl-4-methoxybenzene and 4-methoxybenzoyl chloride were added, following the general procedure. This compound was an off-white solid and was isolated to give 85% yield.

^{1}H NMR (400 MHz, CDCl$_3$) δ 8.17 (s, 2H), 7.61 (s, 2H), 6.95 (d, J = 8.9 Hz, 2H), 6.90 (d, J = 8.8 Hz, 2H), 3.86 (s, 3H), 3.82 (s, 3H).**^{13}C NMR (101 MHz, CDCl$_3$)** δ 177.08, 170.53, 164.69, 164.15, 161.93, 135.33, 132.56, 132.20, 130.77, 129.82, 114.75, 114.18, 112.42, 93.83, 87.15, 55.93, 55.77.

XV. Figure 6: ACA reaction for synthesis of butyl pyridiniums.

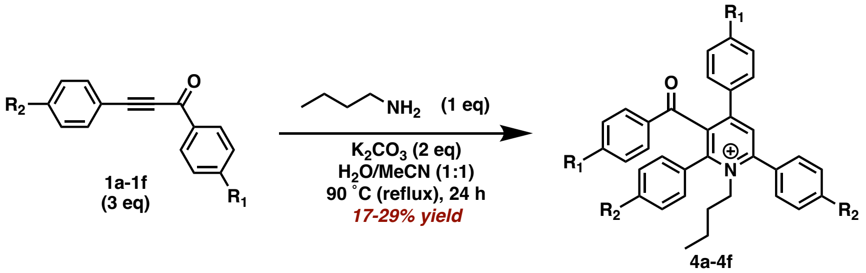

R$_1$ = H, CF$_3$, OMe; R$_2$ = H, OMe

To a 25 mL round-bottom flask (equipped with a stir bar), ynone derivative (0.58 mmol, 3 eq), dissolved in 5 mL of MeCN, and n-butylamine (0.19 mmol, 1 eq) were added. Potassium carbonate (0.38 mmol, 2 eq), dissolved in 5 mL of deionized water, was then added. The reaction was run in an oil bath and under reflux, heated to 90 ˚C. The reaction was left stirring for 24 h, at 1590 rpm, and was analyzed by TLC. After 24 h, the MeCN was evaporated, and DCM was added to the reaction mixture. The water layer was extracted with DCM (x3). The organic layer was dried over sodium sulfate and the crude product was purified by neutral aluminum oxide column chromatography, using ethyl acetate/methanol as eluent. The product eluted at 90% ethyl acetate/ methanol (R_f = 0.2).

3-benzoyl-1-butyl-2,4,6-triphenylpyridin-1-ium (4a)

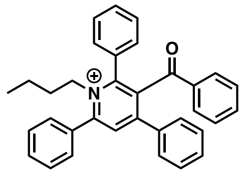

To a 25 mL round-bottom flask, ynone 1a (0.58 mmol, 3 eq), dissolved in 5 mL of MeCN, and n-butylamine (0.19 mmol, 1 eq) were added. Potassium carbonate (0.38 mmol, 2 eq), dissolved in 5 mL of deionized water, was added, following the general procedure. This compound was a yellow oil and was isolated to give 25% yield.

^{1}H NMR (800 MHz, CDCl$_3$) δ 8.88 (s, 1H), 8.45 (d, J = 7.8 Hz, 1H), 7.95 (d, J = 7.7 Hz, 2H), 7.83 (s, 1H), 7.61 (t, J = 7.1 Hz, 3H), 7.49 (dd, J = 7.3, 1.8 Hz, 2H), 7.42 – 7.37 (m, 3H), 7.36 – 7.30 (m, 4H), 7.30 – 7.27 (m, 2H), 7.23 (t, J = 7.7 Hz, 1H), 4.57 (td, J = 12.3, 5.5 Hz, 1H), 4.45 (td, J = 12.5, 5.5 Hz, 1H), 1.49 (h, J = 8.3 Hz, 2H), 0.80 (qd, J = 7.3, 3.4 Hz, 2H), 0.40 (t, J = 7.4 Hz, 3H). **^{13}C NMR (201 MHz, CDCl$_3$)** δ 193.17, 156.60, 155.23, 153.40, 139.44, 135.49, 134.69, 134.55, 132.90, 131.12, 130.93, 130.68, 130.59, 130.29, 129.77, 129.12, 128.99, 128.97, 127.93, 55.33, 31.75, 19.44, 12.64. **HRMS (+ESI):** m/z 468.2321 (calcd $[M]^+$ = 468.2322).

1-butyl-2,6-diphenyl-3-(4-(trifluoromethyl)benzoyl)-4-(4-(trifluoromethyl)phenyl)pyridin-1-ium (4b)

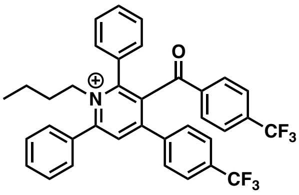

To a 25 mL round-bottom flask, ynone 1b (0.58 mmol, 3 eq), dissolved in 5 mL of MeCN, and n-butylamine (0.19 mmol, 1 eq) were added. Potassium carbonate (0.38 mmol, 2 eq), dissolved in 5 mL of deionized water, was added, following the general procedure. This compound was a yellow oil and was isolated to give 17% yield.

^{1}H NMR (800 MHz, CDCl3) δ 9.22 (s, 1H), 8.88 – 8.77 (m, 1H), 8.50 – 8.39 (m, 1H), 8.15 (d, J = 8.1 Hz, 1H), 7.97 – 7.77 (m, 4H), 7.75 – 7.51 (m, 4H), 7.50 – 7.36 (m, 4H), 7.36 – 7.27 (m, 3H), 4.93 – 4.14 (m, 2H), 1.48 (tt, J = 11.1, 7.2 Hz, 2H), 0.94 – 0.75 (m, 2H), 0.49 – 0.35 (m, 3H). **^{13}C NMR (201 MHz, $CDCl_3$)** δ 192.81, 192.43, 192.21, 157.43, 155.89, 155.64, 155.48, 155.34, 155.25, 153.97, 152.36, 152.12, 140.07, 139.35, 138.80, 135.19, 135.05, 134.21, 132.54, 131.46, 131.42, 131.19, 131.18, 131.07, 130.97, 130.94, 130.78, 130.61, 129.95, 129.27, 129.26, 129.23, 129.17, 129.16, 129.14, 129.12, 128.17, 126.13, 126.11, 55.84, 55.64, 55.57, 31.89, 19.44, 12.59. **^{19}F NMR (753 MHz, $CDCl_3$)** δ -63.03, -63.05, -63.07, -63.28, -63.42, -63.49. **HRMS (+ESI):** m/z 604.2069 (calcd $[M]^+$ = 604.2070).

MS of 4b

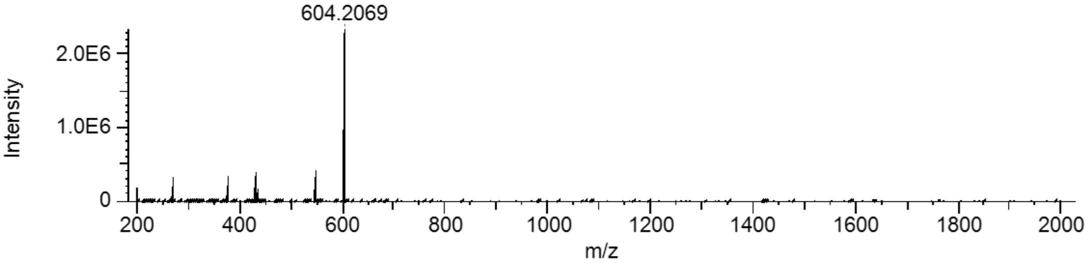

3-benzoyl-1-butyl-2,6-bis(4-methoxyphenyl)-4-phenylpyridin-1-ium (4c)

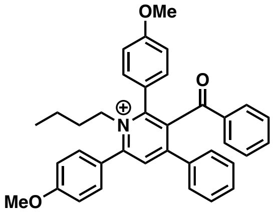

To a 25 mL round-bottom flask, ynone 1c (0.58 mmol, 3 eq), dissolved in 5 mL of MeCN, and n-butylamine (0.19 mmol, 1 eq) were added. Potassium carbonate (0.38 mmol, 2 eq), dissolved in 5 mL of deionized water, was added, following the general procedure. This compound was a yellow oil and was isolated to give 22% yield.

^{1}H NMR (400 MHz, CDCl3) 1H NMR (400 MHz, CDCl3) δ 7.81 (s, 2H), 7.75 (d, J = 7.9 Hz, 3H), 7.62 (s, 3H), 7.49 (d, J = 8.7 Hz, 3H), 7.41 (t, J = 7.6 Hz, 2H), 7.30 (d, J = 7.9 Hz, 3H), 6.91 (d, J = 8.1 Hz, 1H), 6.81 (d, J = 8.8 Hz, 2H), 6.73 (s, 1H), 4.43 (s, 3H), 3.74 (d, J = 2.6 Hz, 6H), 1.43 (s, 2H), 0.81 (q, J = 7.3 Hz, 3H), 0.44 (t, J = 7.3 Hz, 3H). **^{13}C NMR (101 MHz, CDCl3)** δ 178.21, 161.89, 137.21, 135.31, 129.65, 128.71, 114.58, 112.07, 94.46, 87.03, 55.60, 31.73, 22.80, 14.27. **HRMS (+ESI):** m/z 528.2533 (calcd $[M]^+$ = 528.2533).

MS of 4c

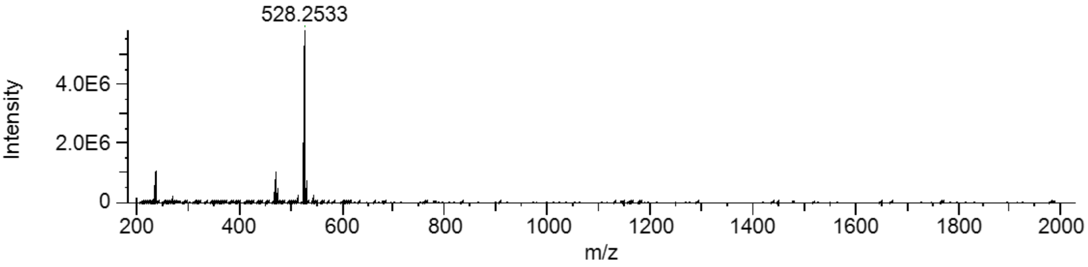

1-butyl-2,6-bis(4-methoxyphenyl)-3-(4-(trifluoromethyl)benzoyl)-4-(4-(trifluoromethyl)phenyl)pyridin-1-ium (4d)

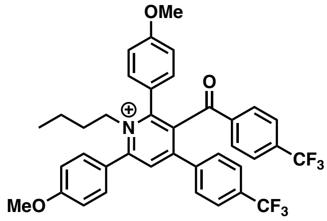

To a 25 mL round-bottom flask, ynone 1d (0.58 mmol, 3 eq), dissolved in 5 mL of MeCN, and n-butylamine (0.19 mmol, 1 eq) were added. Potassium carbonate (0.38 mmol, 2 eq), dissolved in 5 mL of deionized water, was added, following the general procedure. This compound was a yellow oil and was isolated to give 29% yield.

^{1}H NMR (800 MHz, $CDCl_3$) δ 9.06 (s, 1H), 8.59 (d, *J* = 8.0 Hz, 1H), 8.08 (td, *J* = 10.0, 6.2 Hz, 1H), 8.02 – 7.73 (m, 5H), 7.73 – 7.64 (m, 2H), 7.63 – 7.39 (m, 6H), 6.80 (p, *J* = 3.2 Hz, 3H), 4.44 (td, *J* = 13.3, 5.9 Hz, 1H), 4.29 (ddd, *J* = 15.6, 11.9, 5.6 Hz, 1H), 3.89 (dd, *J* = 7.3, 1.9 Hz, 1H), 3.74 (dd, *J* = 6.4, 4.4 Hz, 5H), 1.43 (dp, *J* = 12.5, 7.4 Hz, 2H), 0.81 (tt, *J* = 14.7, 7.3 Hz, 2H), 0.49 – 0.34 (m, 3H). **^{13}C NMR (201 MHz, $CDCl_3$)** δ 191.24, 165.14, 162.19, 155.17, 154.55, 151.99, 139.56, 136.23, 133.39, 133.29, 133.23, 133.03, 132.87, 132.63, 131.97, 131.19, 131.15, 131.08, 130.99, 130.82, 130.34, 130.20, 128.41, 128.27, 126.47, 125.97, 125.95, 124.95, 124.93, 124.23, 124.06, 122.87, 122.70, 114.84, 55.76, 55.48, 31.85, 19.39, 12.56. **^{19}F NMR (753 MHz, $CDCl_3$)** δ -63.06, -63.23. **HRMS (+ESI):** m/z 664.2280 (calcd $[M]^+$ = 664.2281).

MS of 4d

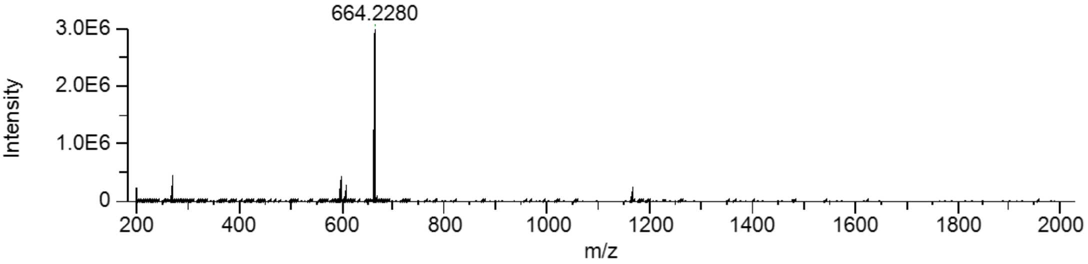

1-butyl-3-(4-methoxybenzoyl)-4-(4-methoxyphenyl)-2,6-diphenylpyridin-1-ium (4e)

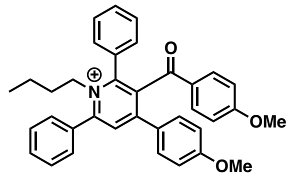

To a 25 mL round-bottom flask, ynone 1e (0.58 mmol, 3 eq), dissolved in 5 mL of MeCN, and n-butylamine (0.19 mmol, 1 eq) were added. Potassium carbonate (0.38 mmol, 2 eq), dissolved in 5 mL of deionized water, was added, following the general procedure. This compound was a yellow oil and was isolated to give 18% yield.

^{1}H NMR (800 MHz, $CDCl_3$) δ 8.49 (d, *J* = 7.8 Hz, 1H), 7.98 – 7.87 (m, 2H), 7.77 (d, *J* = 1.1 Hz, 1H), 7.52 – 7.43 (m, 3H), 7.39 – 7.35 (m, 2H), 7.34 – 7.29 (m, 3H), 7.29 – 7.26 (m, 2H), 7.24 (t, *J* = 7.6 Hz, 1H), 7.10 (s, 2H), 6.78 (d, *J* = 8.6 Hz, 2H), 4.60 (dt, *J* = 14.1, 8.2 Hz, 1H), 4.43 (dt, *J* = 14.0, 8.1 Hz, 1H), 3.88 (s, 3H), 3.75 (s, 3H), 1.44 (h, *J* = 6.7 Hz, 2H), 0.85 – 0.73 (m, 2H), 0.42 (td, *J* = 7.4, 1.1 Hz, 3H). **^{13}C NMR (201 MHz, $CDCl_3$)** δ 191.25, 164.74, 161.59, 156.56, 154.93, 153.22, 139.43, 134.93,

131.16, 130.90, 130.82, 130.60, 130.27, 130.09, 129.10, 129.02, 128.99, 128.95, 128.88, 127.90, 125.16, 114.60, 55.64, 31.73, 19.53, 12.80. **HRMS (+ESI):** m/z 528.2532 (calcd $[M]^+$ = 528.2533).

MS of 4e

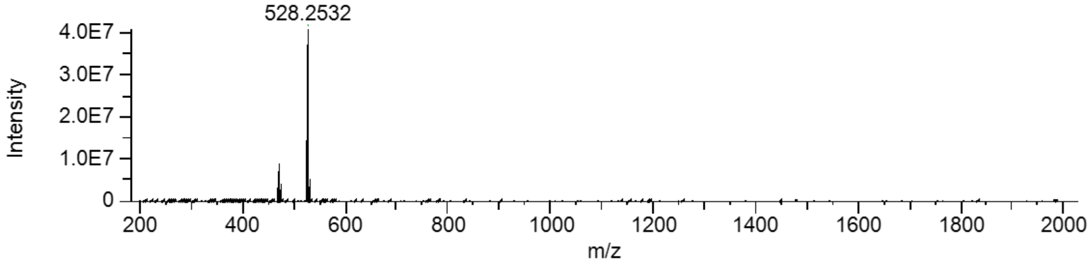

1-butyl-3-(4-methoxybenzoyl)-2,4,6-tris(4-methoxyphenyl)pyridin-1-ium (4f)

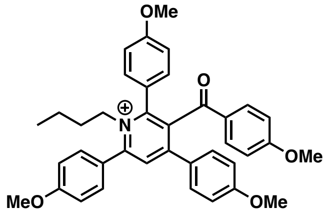

To a 25 mL round-bottom flask, ynone 1f (0.58 mmol, 3 eq), dissolved in 5 mL of MeCN, and n-butylamine (0.19 mmol, 1 eq) were added. Potassium carbonate (0.38 mmol, 2 eq), dissolved in 5 mL of deionized water, was added, following the general procedure. This compound was a yellow oil and was isolated to give 28% yield.

^{1}H NMR (800 MHz, $CDCl_3$) δ 8.41 (dd, J = 8.8, 2.3 Hz, 1H), 7.92 (d, J = 7.6 Hz, 2H), 7.70 (s, 1H), 7.44 (d, J = 8.9 Hz, 2H), 7.29 – 7.25 (m, 2H), 7.08 (s, 2H), 6.85 (dd, J = 8.6, 2.7 Hz, 1H), 6.78 (dd, J = 9.4, 7.5 Hz, 4H), 6.73 (dd, J = 8.7, 2.7 Hz, 1H), 4.56 (ddd, J = 14.1, 10.7, 5.9 Hz, 1H), 4.43 (ddd, J = 14.0, 10.8, 5.7 Hz, 1H), 3.87 (s, 3H), 3.76 (s, 3H), 3.72 (d, J = 2.7 Hz, 6H), 1.43 – 1.33 (m, 2H), 0.80 (hd, J = 7.0, 4.0 Hz, 2H), 0.44 (t, J = 7.4 Hz, 3H). **^{13}C NMR (201 MHz, $CDCl_3$)** δ 191.81, 164.68, 161.66, 161.48, 161.06, 156.18, 154.28, 153.24, 138.99, 132.87, 131.95, 130.99, 130.12, 128.86, 127.07, 125.43, 122.15, 114.57, 114.54, 114.20, 113.47, 55.75, 55.61, 55.42, 55.36, 54.85, 31.70, 19.56, 12.91. **HRMS (+ESI):** m/z 588.2736 (calcd $[M]^+$ = 588.2744).

MS of 4f

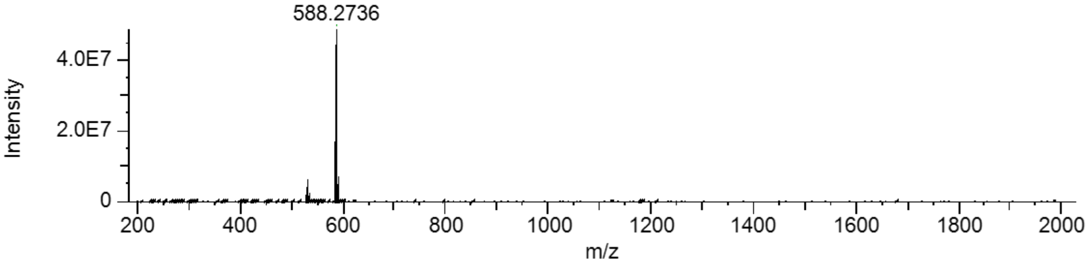

XVI. Figure 7: UV-Vis spectra of butyl pyridiniums 4a-4f.

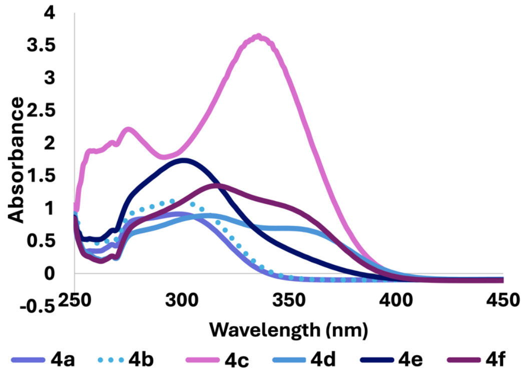

UV/Vis was measured in 1:1 DMSO/Water (50 μM)

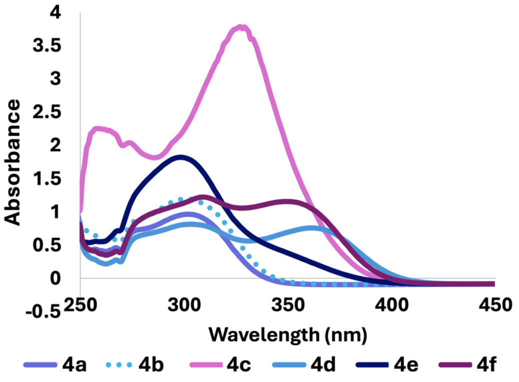

UV/Vis was measured in ethanol (50 μM)

XVII. Figure 8: Fluorescence and quantum yield calculations of butyl pyridiniums.

Fluorescence of pyridiniums **4d**, **4e**, and **4f** was measured at λ_{ex}= 358 nm, in 1:1 DMSO/water (50 μM).

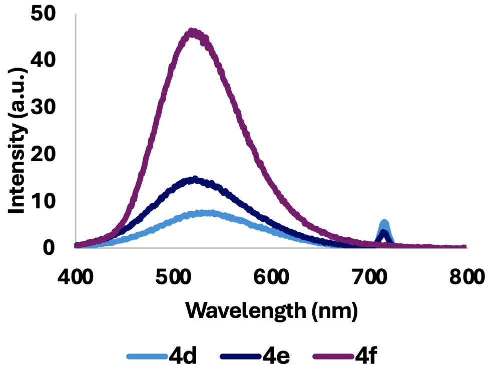

Quantum Yield Studies of 4e and 4f

Quantum yield was calculated using the area under the curve of fluorescence versus absorption. Absorption was measured with a Agilent Cary 3500 UV-Vis utilizing the samples at using four separate concentrations with values being divided by ten for accurate analysis. Fluorescence area was measured with a Cary Eclipse fluorimeter using the same samples above with a 10X dilution. All measurements were run in triplicate. Quantum yields of 4e and 4f were determined using Cy2 as a reference compound.

$$Q = Qr(Ms/Mr)(ns/Nr)^2$$

Q is the quantum yield; m is the slope of the line described above; n is the refractive index of the solvent. Subscript r denotes the appropriate values for the reference (Cy2). All studies are done in 1:1 DMSO/water.

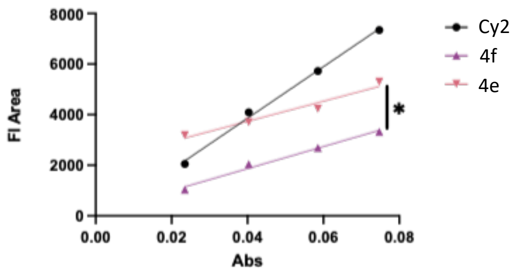

Compound	Quantum Yield (φ)
**4e**	0.0267283
**4f**	0.03104841

XVIII. Figure 9: Chemoselectivity of ACA reaction.

To 1.3 µmol of peptide, dissolved in 217 µL of solvent (6 mM), 2.1 mg of ynone 1f (8 µmol, 6 eq) was added. All reactions were run in a 2 mL glass vial, equipped with two stir bars. All of the reactions were run in an oil bath at 80 ˚C and were left stirring, at 1600 rpm, for 15 h. The solvent used for the reactions is a Buffer/MeCN mixture (1:1). The buffer is 10 mM NaP/12 mM $NaHCO_3$ (pH 8). Samples were taken from the reaction mixture and injected into the HPLC and MS to monitor the reaction. The reaction mixture was analyzed by the HPLC method reported in the analytical method. Percent conversion was determined by calculating the area under the HPLC peaks of reaction mixture.

Reactivity of Trp

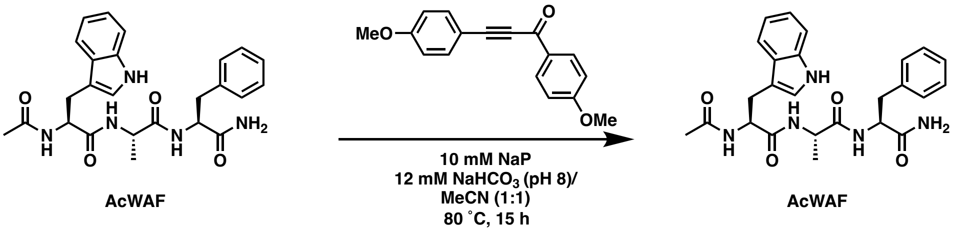

AcWAF. HRMS (+ESI): m/z 486.2110 (calcd [M+Na]$^+$ = 486.2118), m/z 927.4511 (calcd [2M]$^+$ = 926.4440), m/z 949.4328 (calcd [2M+Na]$^+$ = 949.4338). (HPLC analysis at 220 nm). Retention time on HPLC: 12.853.

HPLC of AcWAF

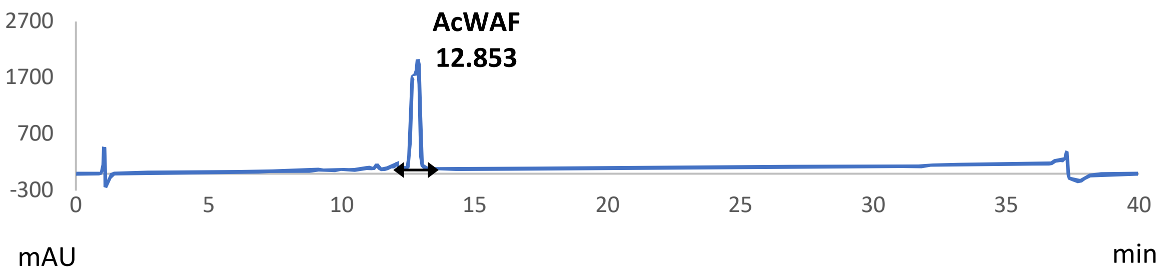

MS of AcWAF

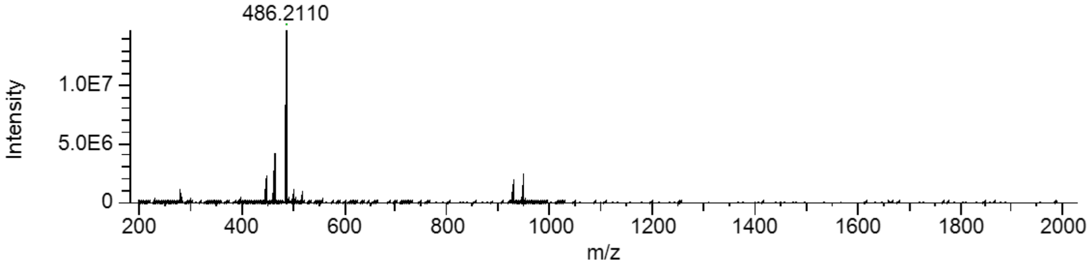

HPLC of AcWAF under ACA reaction

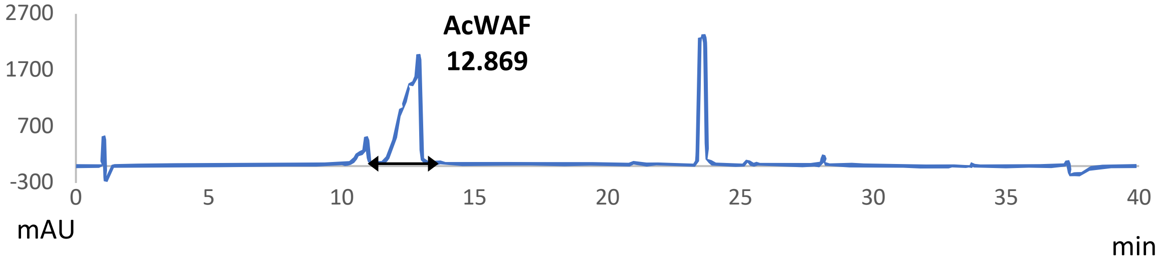

Reactivity of Ser, Met and Arg

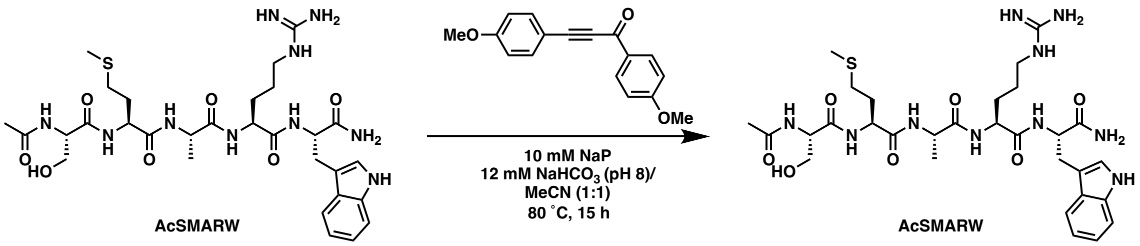

AcSMARW. HRMS (+ESI): m/z 691.3315 (calcd $[M+H]^+$ = 691.3344). (HPLC analysis at 220 nm). Retention time on HPLC: 8.

HPLC of AcSMARW

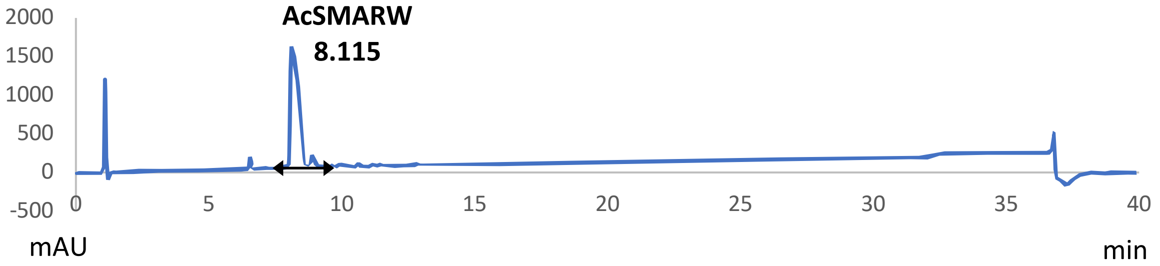

MS of AcSMARW

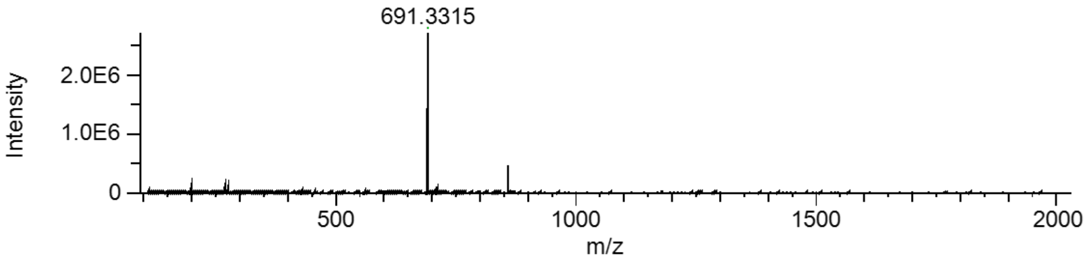

HPLC of AcSMARW under ACA reaction

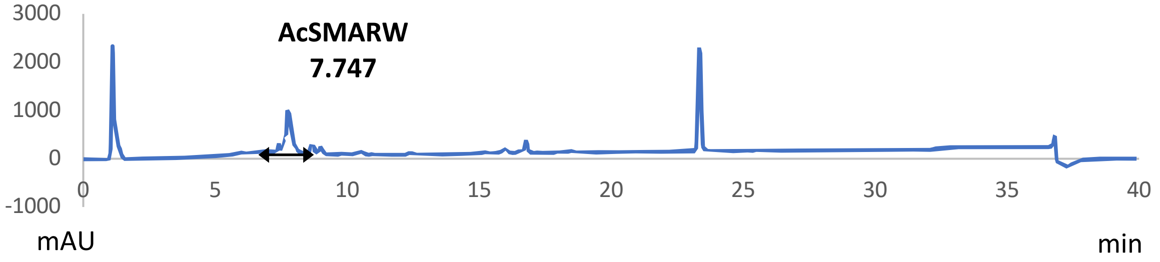

Reactivity of Asp and Asn

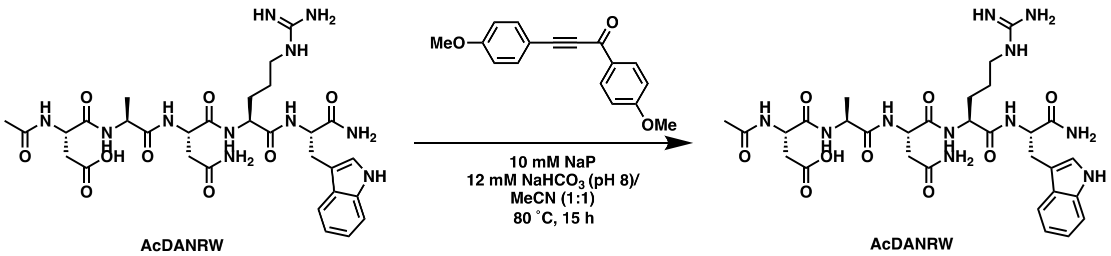

AcDANRW. HRMS (+ESI): m/z 702.3288 (calcd $[M+H]^+$ = 702.3318). (HPLC analysis at 220 nm). Retention time on HPLC: 6.

HPLC of AcDANRW

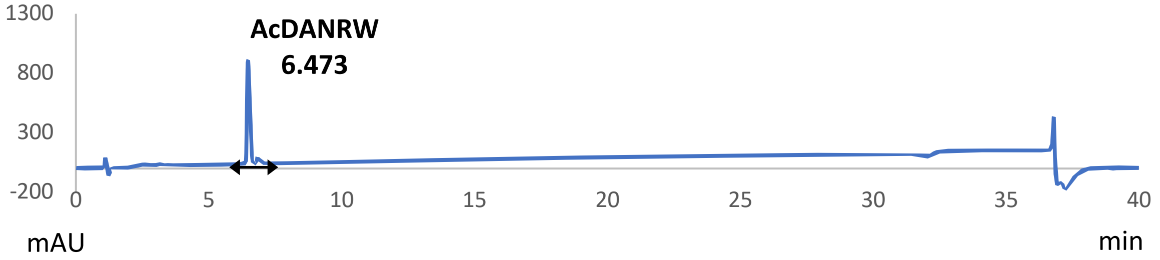

MS of AcDANRW

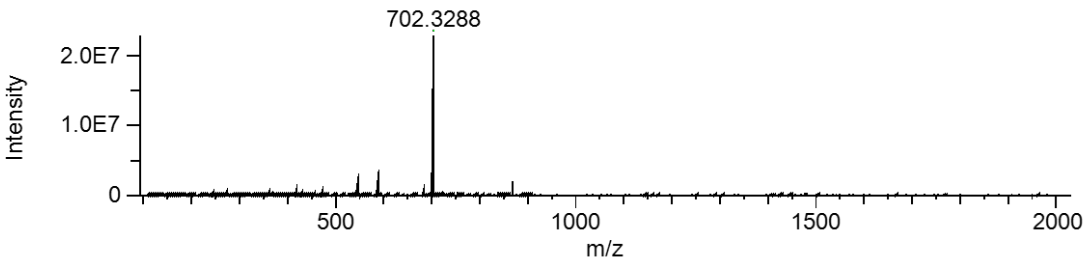

HPLC of AcDANRW under ACA reaction

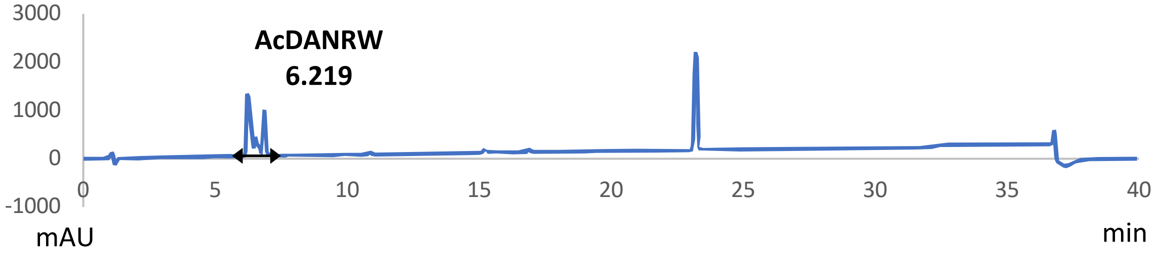

Reactivity of Tyr

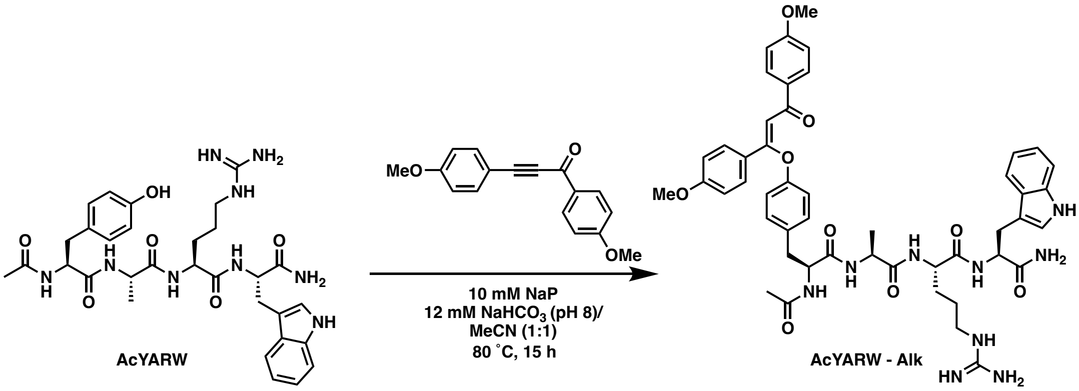

AcYARW. HRMS (+ESI): m/z 636.3222 (calcd $[M+H]^+$ = 636.3253). (HPLC analysis at 220 nm). Retention time on HPLC: 8.

AcYARW - Alk. HRMS (+ESI): m/z 902.4139 (calcd $[M+H]^+$ = 902.4196), m/z 451.7105 (calcd $[(M+2H)/2]^+$ = 451.7134). (HPLC analysis at 220 nm). Retention time on HPLC: 15. Peak Area on HPLC: 19303.7 mAU*s (**52 %**).

HPLC of AcYARW

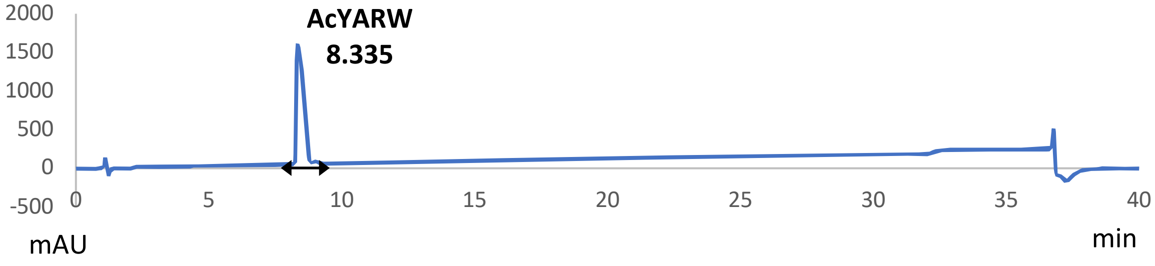

MS of AcYARW

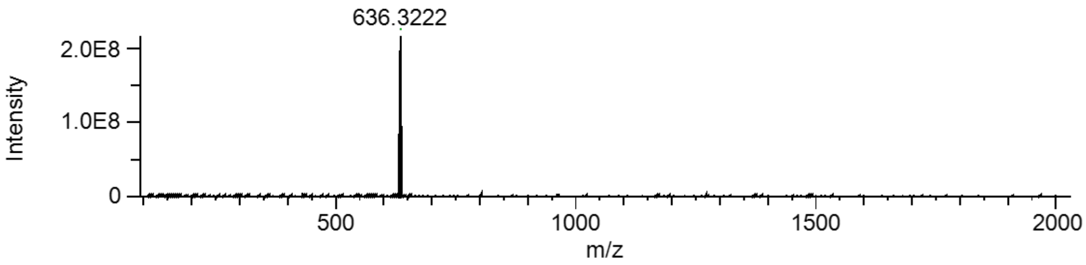

HPLC of AcYARW under ACA reaction

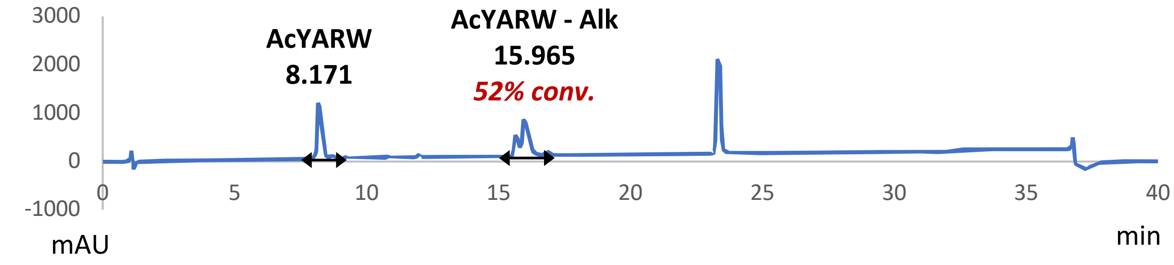

MS of AcYARW - Alk

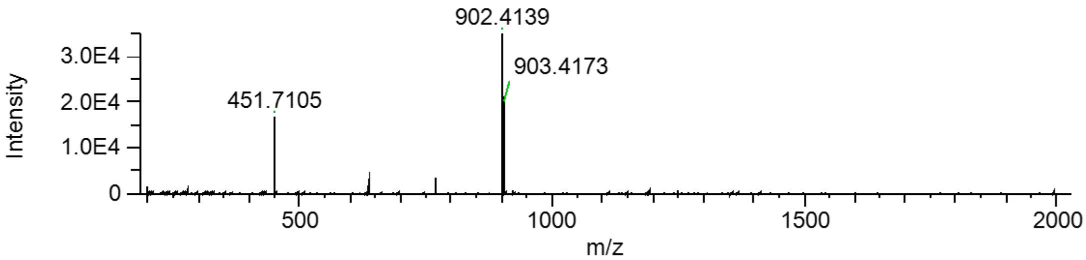

Reactivity of His

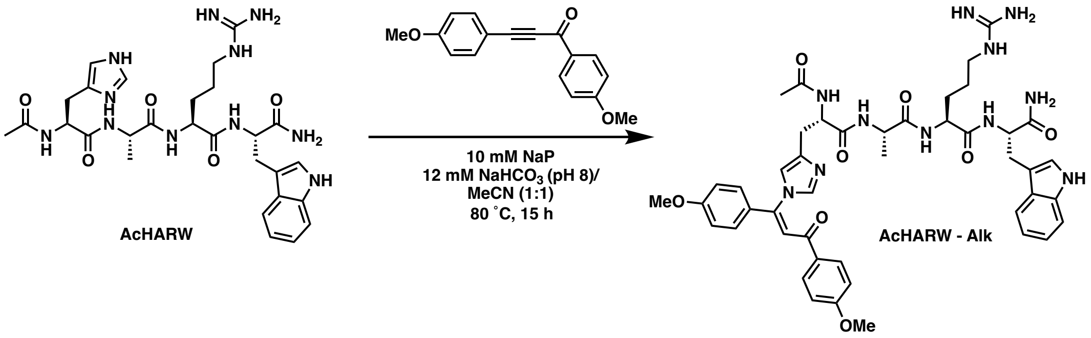

AcHARW. HRMS (+ESI): m/z 610.3176 (calcd $[M+H]^+$ = 610.3208), m/z 305.6624 (calcd $[(M+2H)/2]^+$ = 305.6640). (HPLC analysis at 220 nm). Retention time on HPLC: 4.

AcHARW - Alk. HRMS (+ESI): m/z 876.4113 (calcd $[M+H]^+$ = 876.4151), m/z 438.7093 (calcd $[(M+2H)/2]^+$ = 438.7112). Retention time on HPLC: 12. Peak Area on HPLC: 31983.2 mAU*s (**51 %**).

HPLC of AcHARW

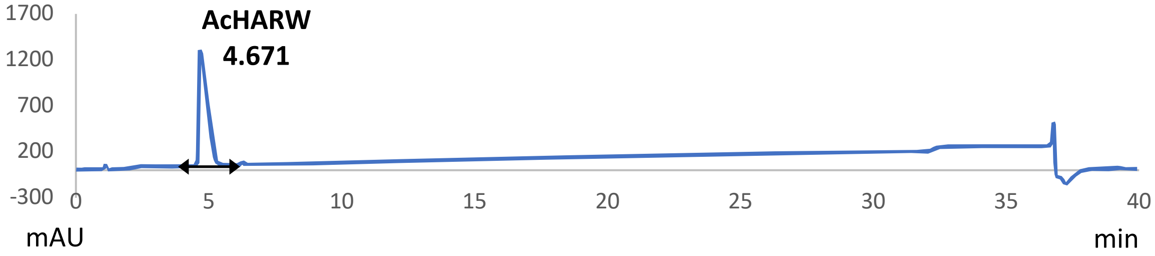

MS of AcHARW

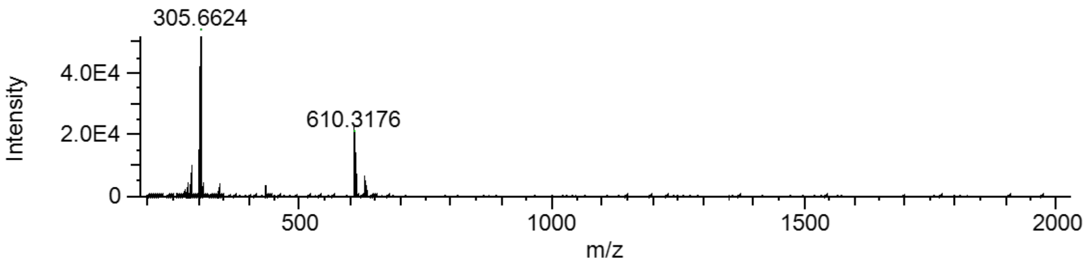

HPLC of AcHARW under ACA reaction

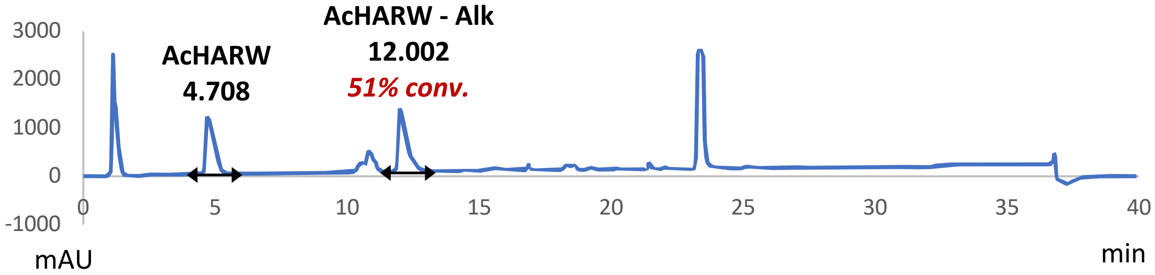

MS of AcHARW - Alk

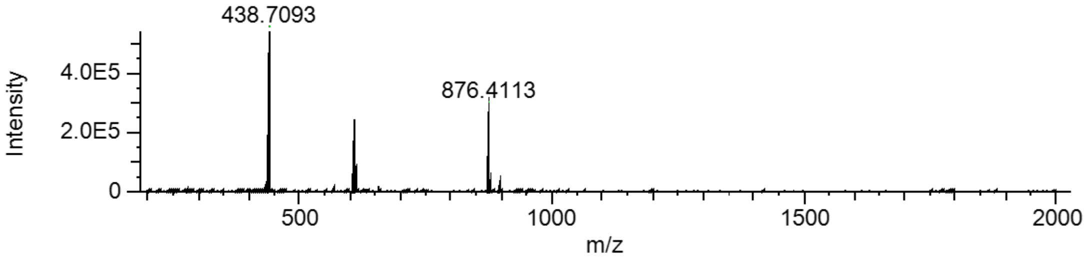

Reactivity of Cys

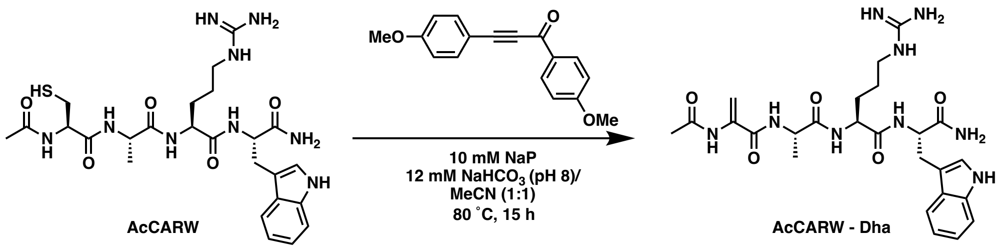

AcCARW. HRMS (+ESI): m/z 576.2680 (calcd $[M+H]^+$ = 576.2711). (HPLC analysis at 220 nm). Retention time on HPLC: 7.

AcCARW - Dha. HRMS (+ESI): m/z 542.2781 (calcd $[M+H]^+$ = 542.2834). (HPLC analysis at 220 nm). Retention time on HPLC: 7. Peak Area on HPLC: 13636.4 mAU*s (**>99 %**).

HPLC of AcCARW

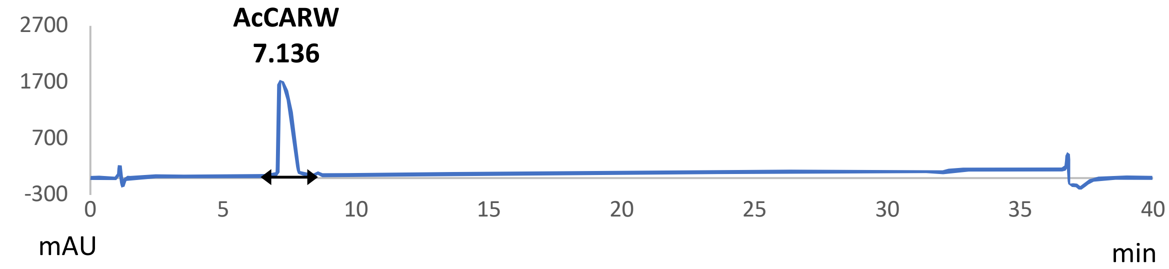

MS of AcCARW

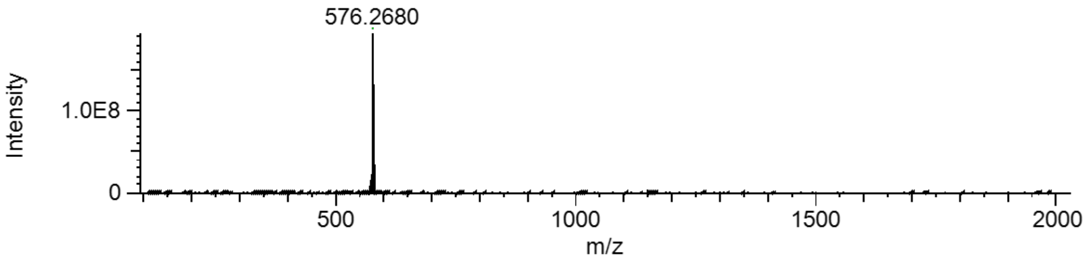

HPLC of AcCARW under ACA reaction

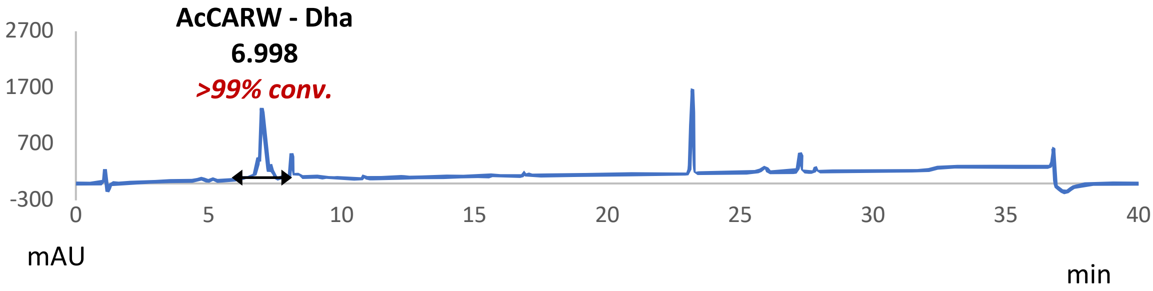

MS of AcCARW - Dha

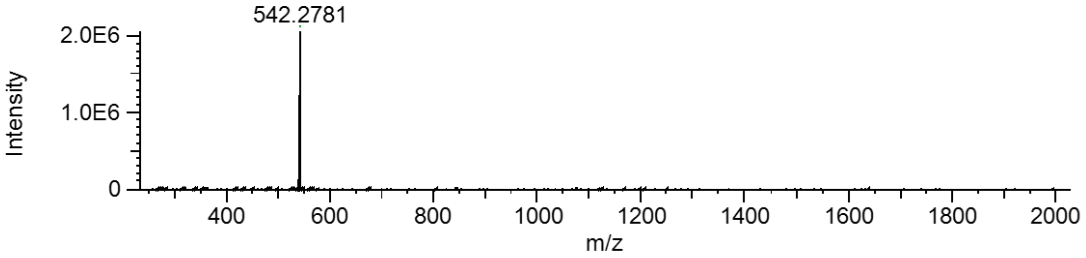

Reactivity of N-terminus

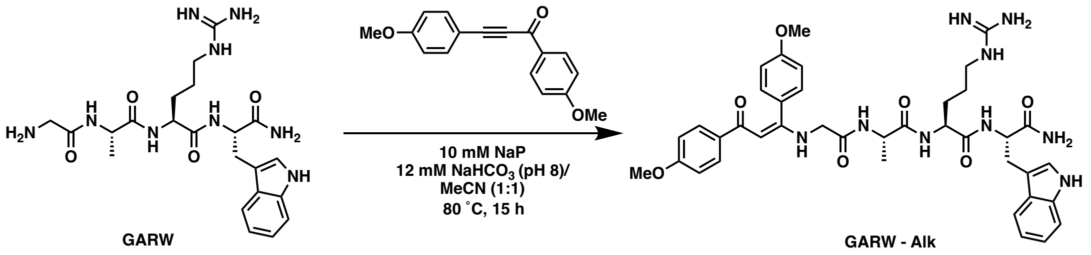

GARW. HRMS (+ESI): m/z 488.2705 (calcd $[M+H]^+$ = 488.2728), m/z 244.6389 (calcd $[(M+2H)/2]^+$ = 244.6400). (HPLC analysis at 220 nm). Retention time on HPLC: 4.

GARW - Alk. HRMS (+ESI): m/z 754.3646 (calcd $[M+H]^+$ = 754.3671), m/z 377.6858 (calcd $[(M+2H)/2]^+$ = 377.6872). (HPLC analysis at 220 nm). Retention time on HPLC: 14.757. Peak Area on HPLC: 39972.9 mAU*s (**90 %**).

HPLC of GARW

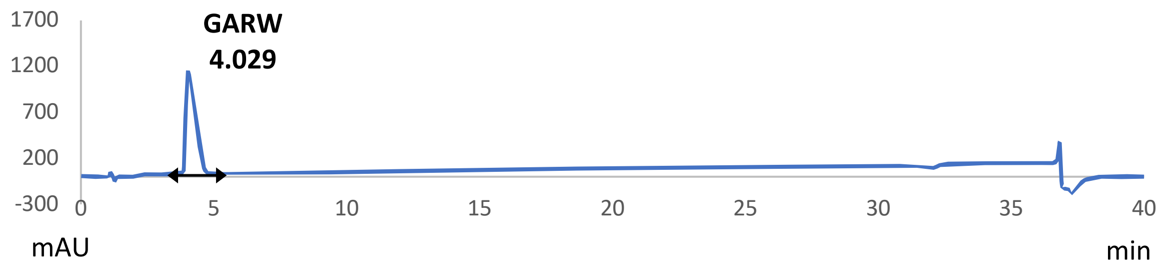

MS of GARW

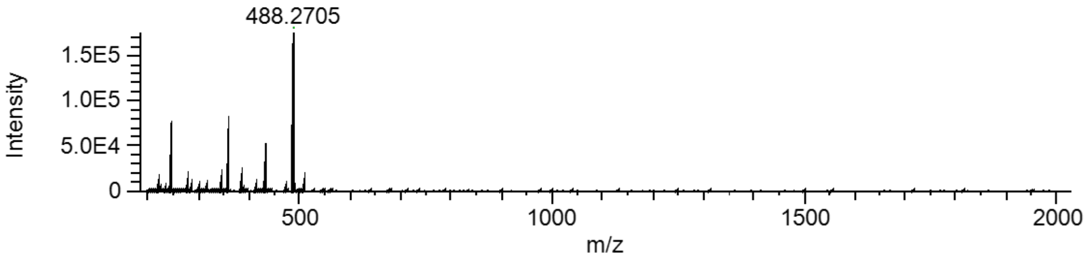

HPLC of GARW under ACA reaction

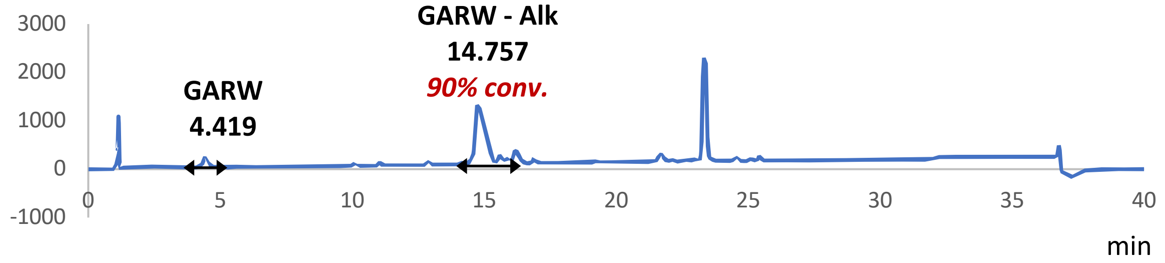

MS of GARW - Alk

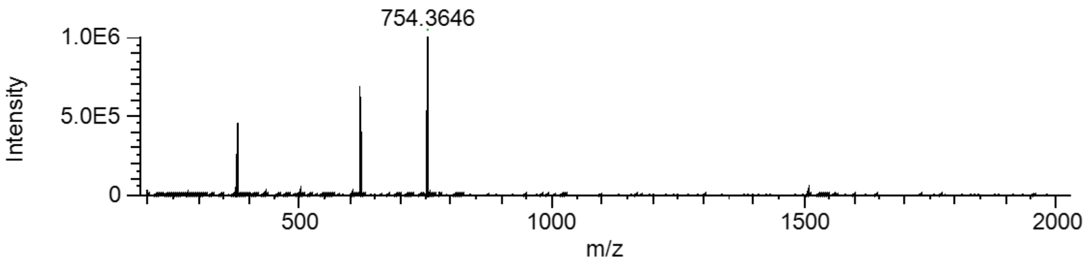

Reactivity of Lys

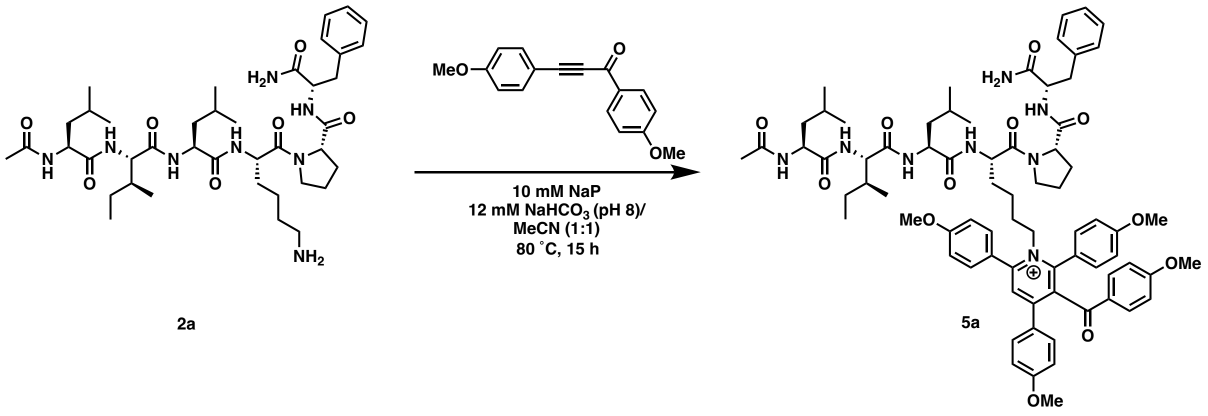

Peptide 5a. HRMS (+ESI): m/z 1285.6898 (calcd $[M]^+$ = 1285.6907), m/z 643.3493 (calcd $[(M+H)/2]^+$ = 643.3490). (HPLC analysis at 220 nm). Retention time on HPLC: 19.020. Peak Area on HPLC: 75019.6 mAU*s (>**99 %**).

HPLC of Peptide 2a under ACA reaction

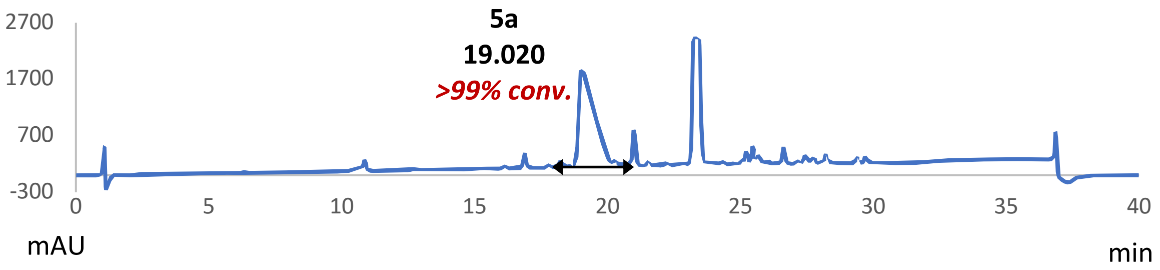

MS of Peptide 5a

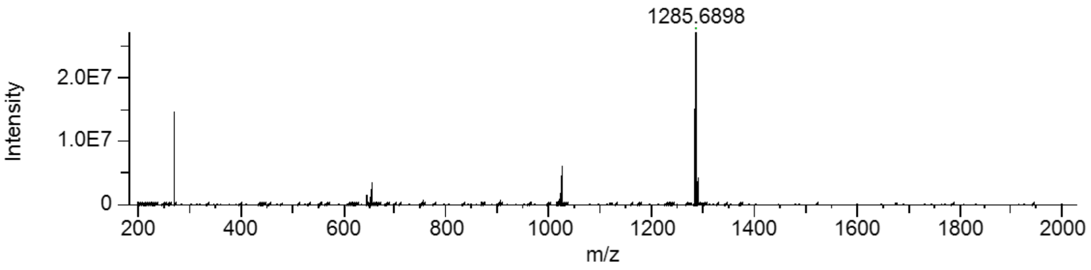

XIX. Figure 10: Reversing Tyr and His alkylation.

First, to 1.3 µmol of peptide, dissolved in 217 µL of solvent (6 mM), 2.1 mg of ynone 1f (8 µmol, 6 eq) was added. All reactions were run in a 2 mL glass vial, equipped with two stir bars. The first step was run in an oil bath at 80 ˚C and was left stirring, at 1600 rpm, for 15 h. The solvent used for the reactions is a Buffer/MeCN mixture (1:1). The buffer is 10 mM NaP/12 mM $NaHCO_3$ (pH 8).

After 15 h, 1, 1.9, or 3.8 µL of butylamine (10, 20, or 39 µmol; 8, 15, or 30 eq) was added to the crude reaction mixture, to determine the reversibility of the alkylated product. The reaction was stirred at 65 ˚C, 1600 rpm, for 8 h. Samples were taken from the reaction mixture and injected into the HPLC and MS to monitor the reaction. The reaction mixture was analyzed by the HPLC method reported in the

analytical method. Percent conversion was determined by calculating the area under the HPLC peaks of reaction mixture.

Reversing Tyr

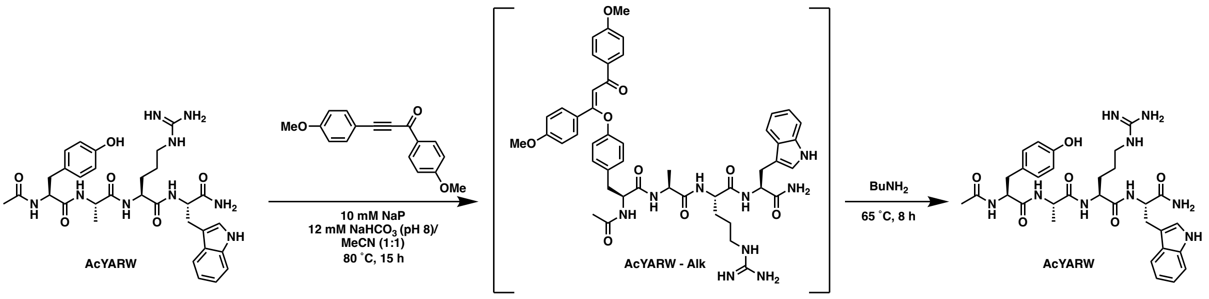

Entry	Butylamine Eq.	Conversion of AcYARW-Alk:AcYARW (%)
1	8	15:85
2	15	7:93
3	30	0:>99

AcYARW. HRMS (+ESI): m/z (calcd $[M+H]^+$ = 636.3253). (HPLC analysis at 220 nm). Retention time on HPLC: 8.

AcYARW - Alk. HRMS (+ESI): m/z (calcd $[M+H]^+$ = 902.4196). (HPLC analysis at 220 nm). Retention time on HPLC: 15-16.

HPLC of Entry 1

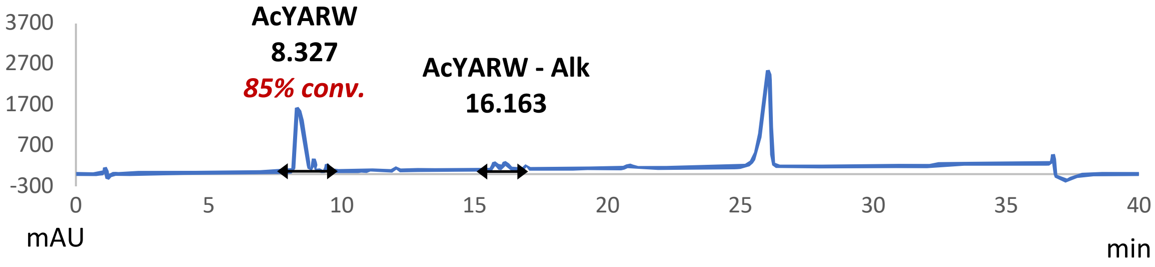

HPLC of Entry 2

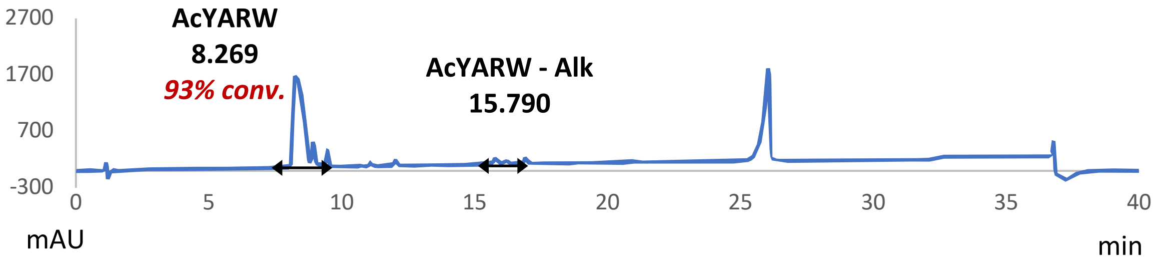

HPLC of Entry 3

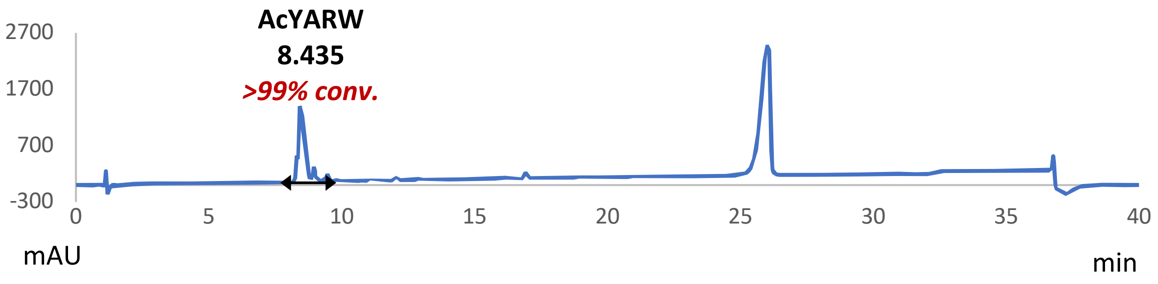

Reversing His

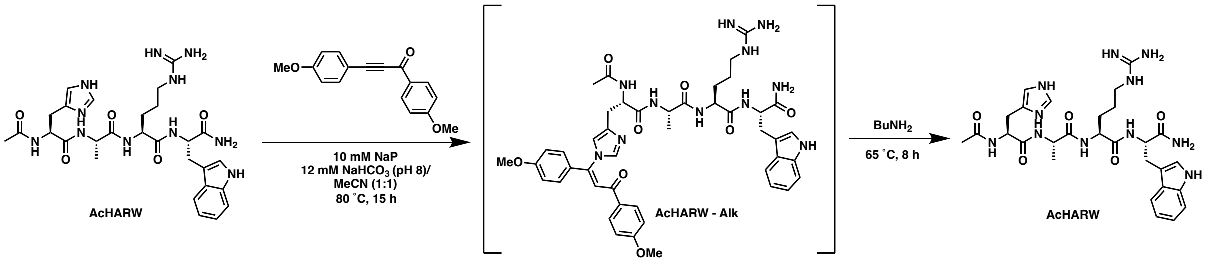

Entry	Butylamine Eq.	Conversion of AcHAF-Alk:AcHAF (%)
1	8	0:>99
2	15	0:>99
3	30	0:>99

AcHARW. HRMS (+ESI): m/z (calcd $[M+H]^+$ = 610.3208). (HPLC analysis at 220 nm). Retention time on HPLC: 4.

AcHARW - Alk. HRMS (+ESI): m/z (calcd $[M+H]^+$ = 876.4151). Retention time on HPLC: 12.

HPLC of Entry 1

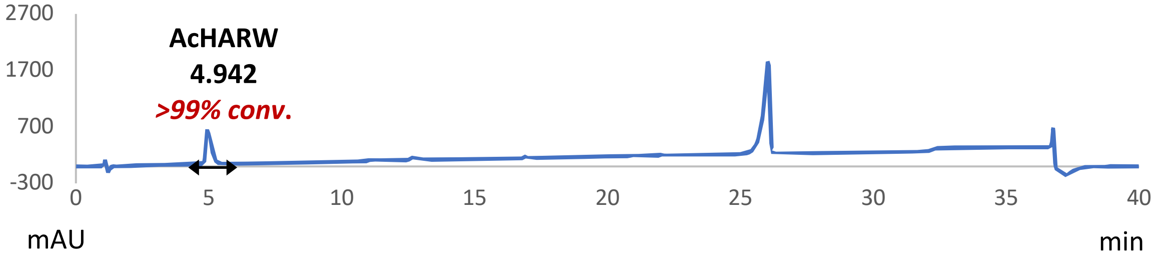

HPLC of Entry 2

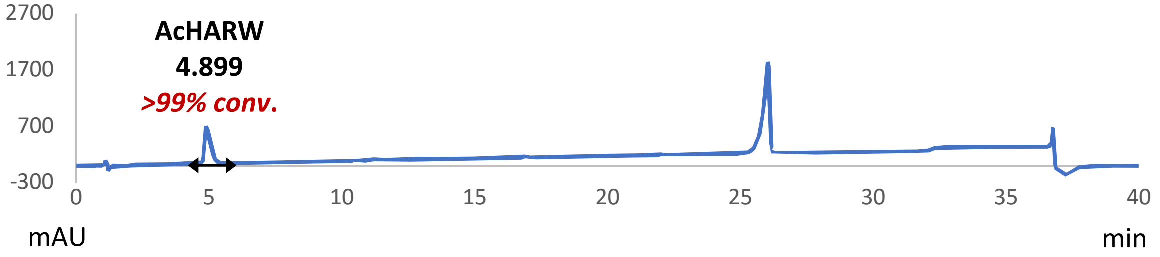

HPLC of Entry 3

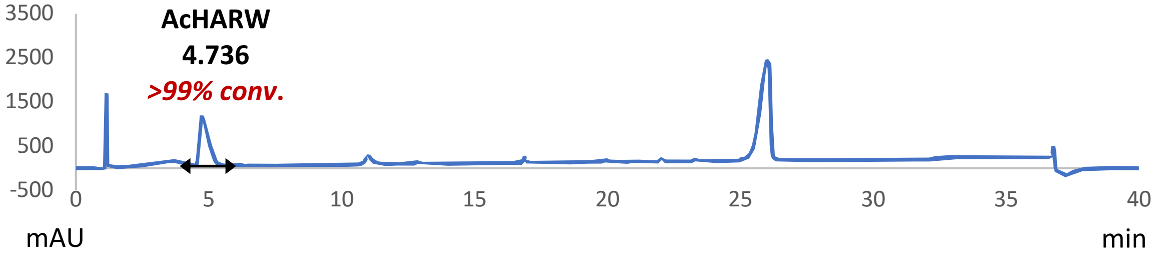

Stability of Lys pyridinium under reversing condition

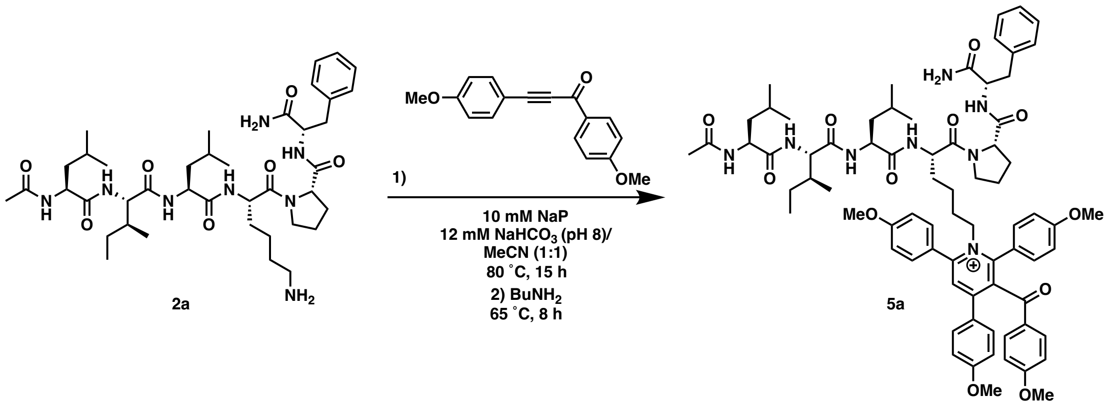

Entry	Butylamine Eq.	Conversion of 2a:5a (%)
1	8	2:97
2	15	3:96
3	30	6:93

HPLC of Entry 1

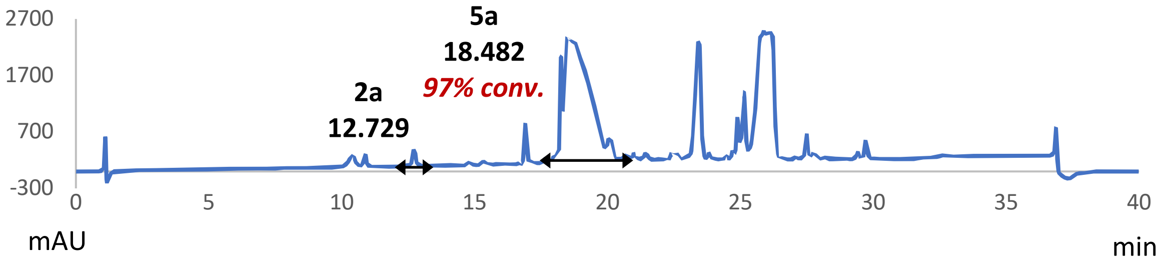

HPLC of Entry 2

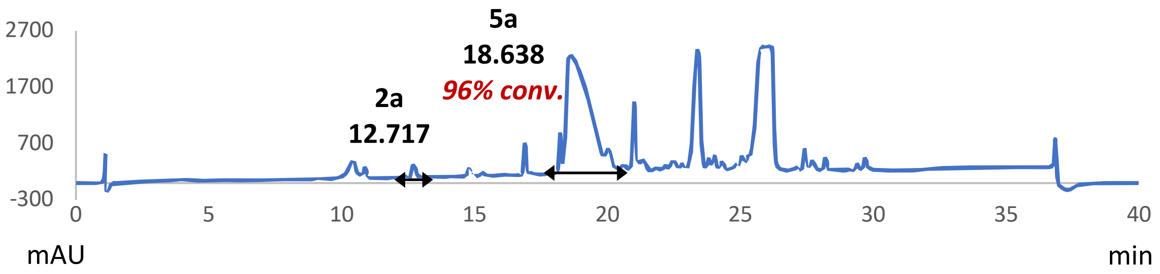

HPLC of Entry 3

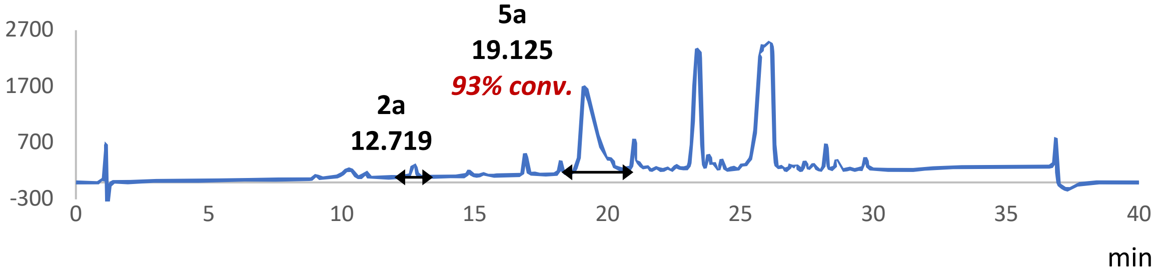

XX. Figure 11: Synthesis of ACA-pyridinium peptides.

To 1.3 µmol of peptide, dissolved in 217 µL of solvent (6 mM), 2.1 mg of ynone 1f (8 µmol, 6 eq) was added. All reactions were run in a 2 mL glass vial, equipped with two stir bars. All of the reactions were run in an oil bath at 80 ˚C and were left stirring, at 1600 rpm, for 15 h. The solvent used for the reactions is a Buffer/MeCN mixture (1:1). The buffer is 10 mM NaP/12 mM $NaHCO_3$ (pH 8). Samples were taken from the reaction mixture and injected into the HPLC and MS to monitor the reaction. The reaction mixture was analyzed by the HPLC method reported in the analytical method. Percent conversion was determined by calculating the area under the HPLC peaks of reaction mixture.

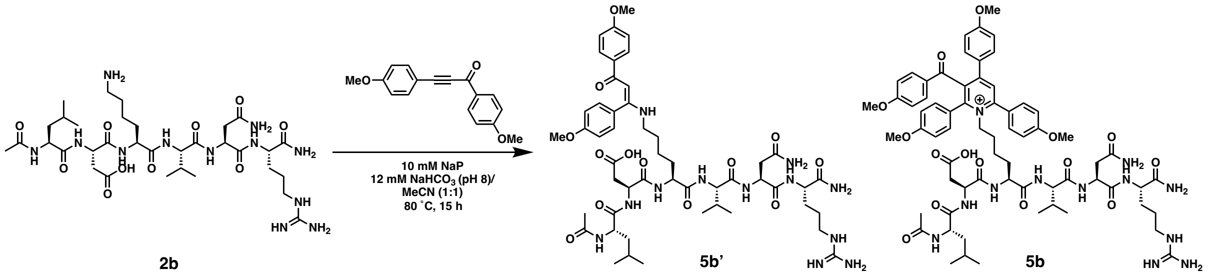

Peptide 2b. HRMS (+ESI): m/z 785.4622 (calcd $[(M+H)]^+$ = 785.4628), m/z 393.2349 (calcd $[(M+2H)/2]^+$ = 393.2351). (HPLC analysis at 220 nm). Retention time on HPLC: 5.106.

Peptide 5b'. HRMS (+ESI): m/z 1051.5565 (calcd $[M+H]^+$ = 1051.5571), m/z 526.2823 (calcd $[(M+2H)/2]^+$ = 526.2822). (HPLC analysis at 220 nm). Retention time on HPLC: 15.698. Peak Area on HPLC: 4928.9 mAU*s (**15 %**).

Peptide 5b. HRMS (+ESI): m/z 1299.6401 (calcd $[M]^+$ = 1299.6408), m/z 650.3242 (calcd $[(M+H)/2]^+$ = 650.3241). (HPLC analysis at 220 nm). Retention time on HPLC: 14.364. Peak Area on HPLC: 28260.1 mAU*s (**85 %**).

HPLC of Peptide 2b

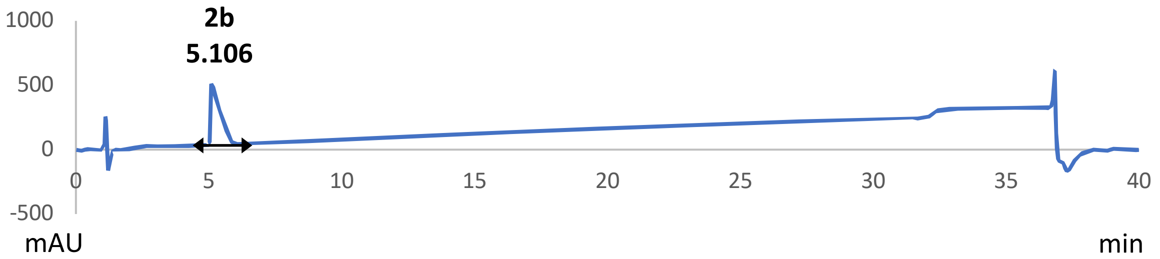

MS of Peptide 2b

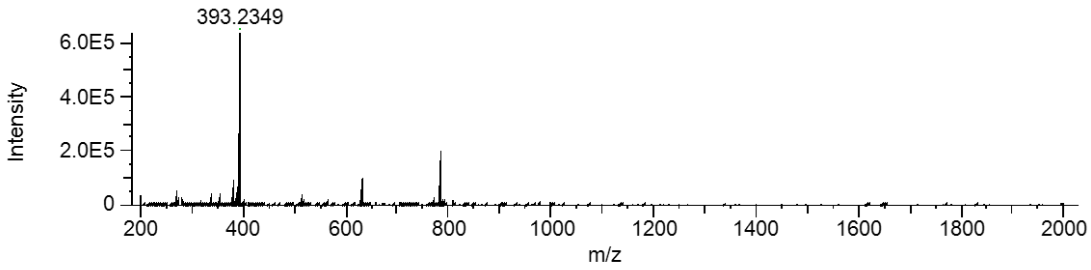

HPLC of Peptide 2b under ACA reaction

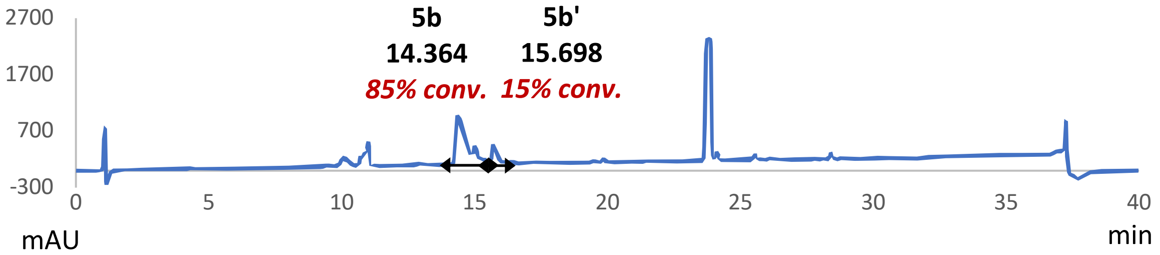

MS of Peptide 5b

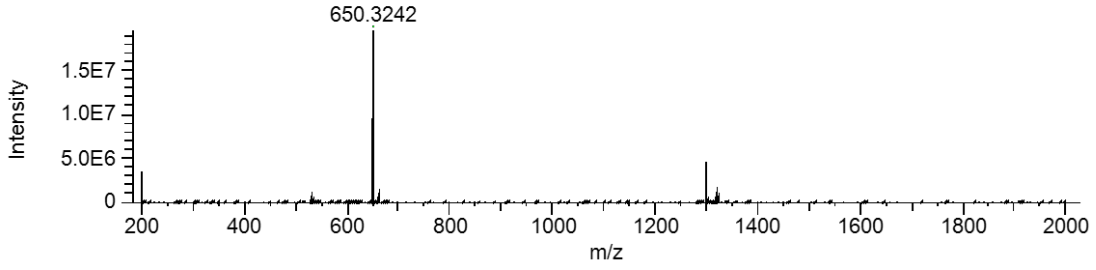

MS of Peptide 5b’

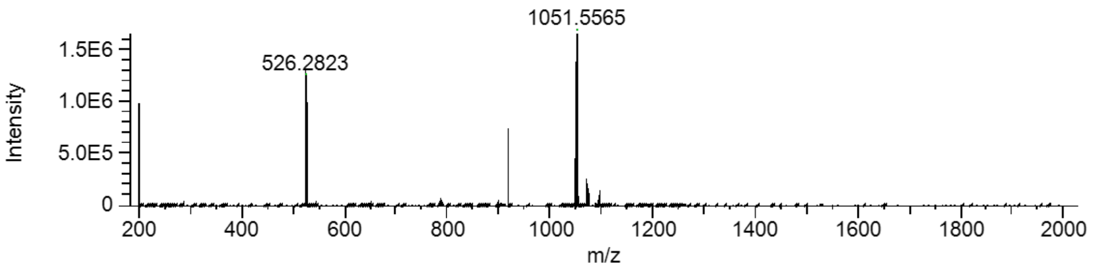

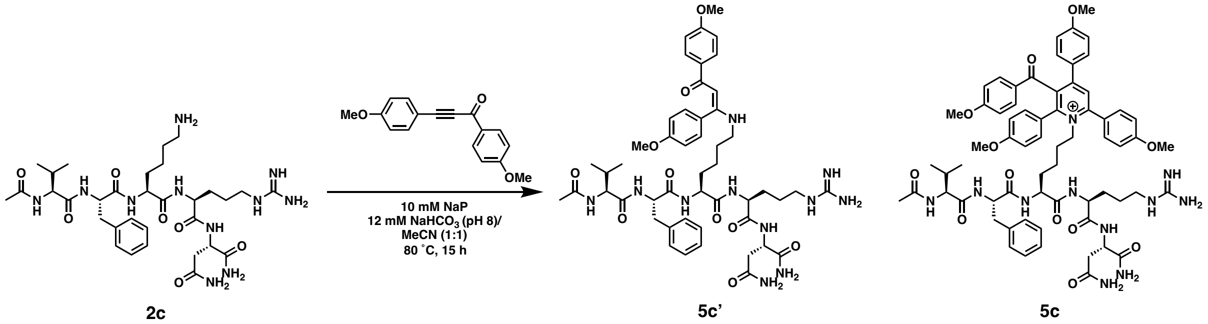

Peptide 2c. HRMS (+ESI): m/z 704.4141 (calcd $[M+H]^+$ = 704.4202), m/z 352.7100 (calcd $[(M+2H)/2]^+$ = 352.7138). (HPLC analysis at 220 nm). Retention time on HPLC: 4.345.

Peptide 5c'. HRMS (+ESI): m/z 970.5136 (calcd $[M+H]^+$ = 970.5145), m/z 485.7607 (calcd $[(M+2H)/2]^+$ = 485.7609), m/z 992.4946 (calcd $[(M+Na]^+$ = 992.4970). (HPLC analysis at 220 nm). Retention time on HPLC: 16.222. Peak Area on HPLC: 12086.1 mAU*s (**20 %**).

Peptide 5c. HRMS (+ESI): m/z 1218.5975 (calcd $[M]^+$ = 1218.5982), m/z 609.8028 (calcd $[(M+H)/2]^+$ = 609.8028). (HPLC analysis at 220 nm). Retention time on HPLC: 14.675. Peak Area on HPLC: 48727.2 mAU*s (**80 %**).

HPLC of Peptide 2c

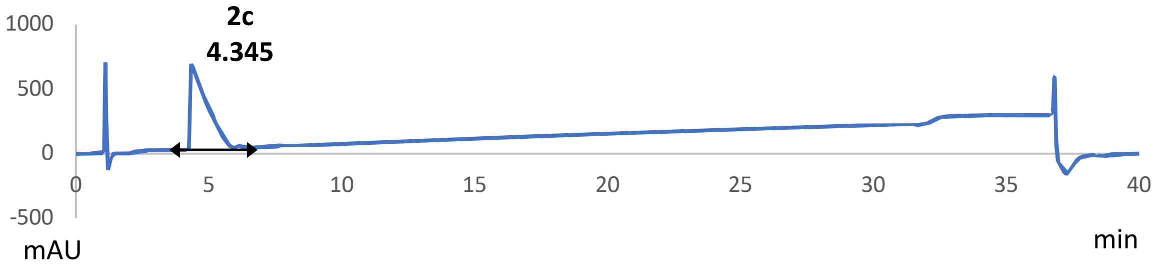

MS of Peptide 2c

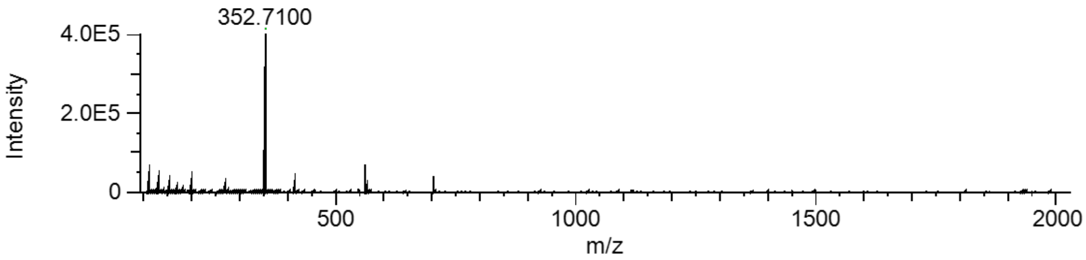

HPLC of Peptide 2c under ACA reaction

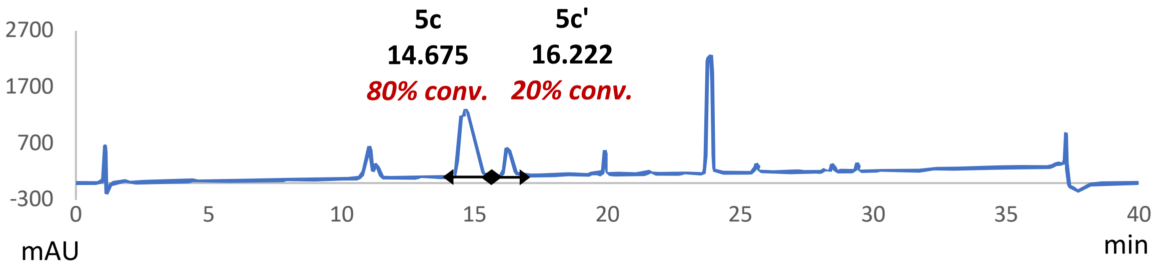

MS of Peptide 5c

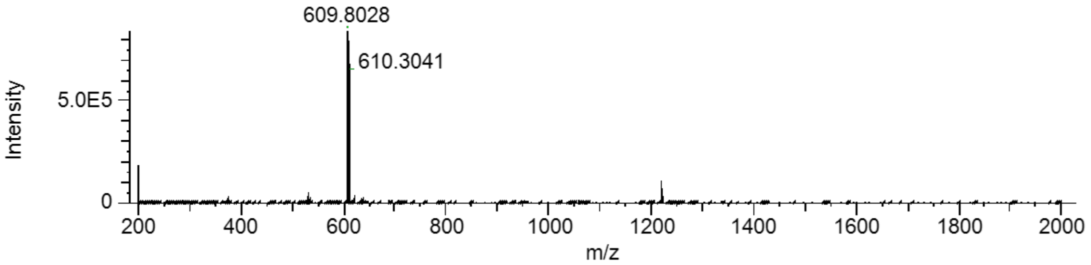

MS of Peptide 5c'

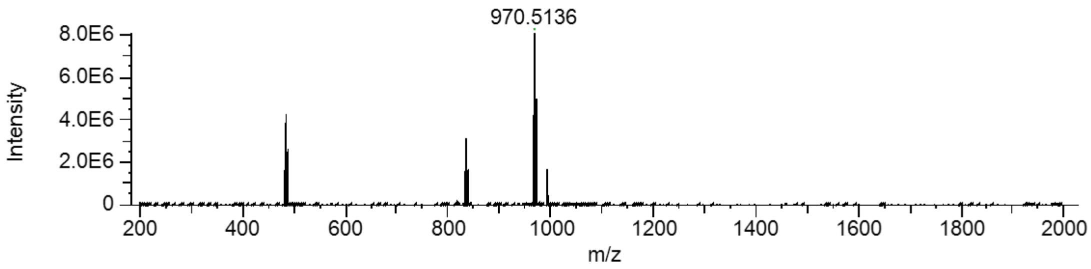

XXI. Figure 12: Synthesis of ACA-pyridinium peptides containing Tyr and His.

First, to 1.3 µmol of peptide, dissolved in 217 µL of solvent (6 mM), 2.8 mg of ynone 1f (10.4 µmol, 8 eq) was added. All reactions were run in a 2 mL glass vial, equipped with two stir bars. The first step was run in an oil bath at 80 ˚C and was left stirring, at 1600 rpm, for 15 h. The solvent used for the reactions is a Buffer/MeCN mixture (1:1). The buffer is 10 mM NaP/12 mM $NaHCO_3$ (pH 8). After 15 h, 3.8 µL of butylamine (39 µmol, 30 eq) was added to the crude reaction mixture. The reaction was stirred at 65 ˚C, 1600 rpm, for 8 h. Samples were taken from the reaction mixture and injected into the HPLC and MS to monitor the reaction. The reaction mixture was analyzed by the HPLC method reported in the analytical method. Percent conversion was determined by calculating the area under the HPLC peaks of reaction mixture.

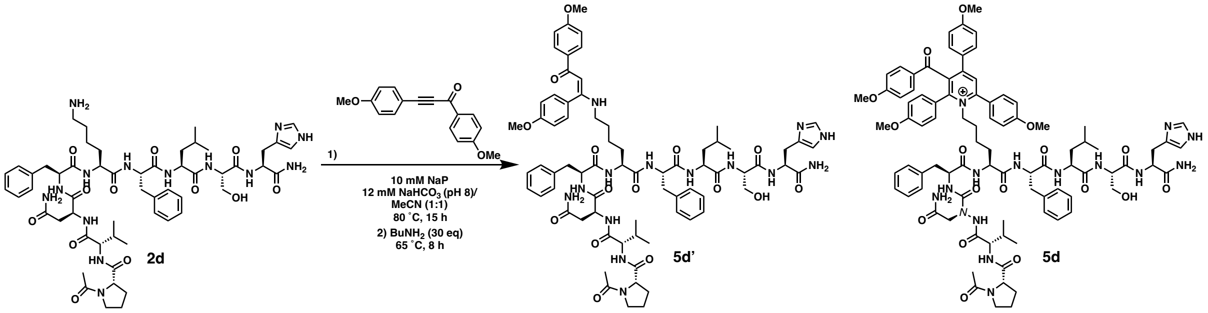

Peptide 2d. HRMS (+ESI): m/z 1129.6144 (calcd $[M+H]^+$ = 1129.6153), m/z 1151.5958 (calcd $[M+Na]^+$ = 1151.5978), m/z 565.3112 (calcd $[(M+2H)/2]^+$ = 565.3113). (HPLC analysis at 220 nm). Retention time on HPLC: 9.714.

Peptide 5d'. HRMS (+ESI): m/z 1395.7083 (calcd $[M+H]^+$ = 1395.7096), m/z 1417.6899 (calcd $[M+Na]^+$ = 1417.6921), m/z 698.3585 (calcd $[(M+2H)/2]^+$ = 698.3585). (HPLC analysis at 220 nm). Retention time on HPLC: 17.197. Peak Area on HPLC: 25518.7 mAU*s (**55 %**).

Peptide 5d. HRMS (+ESI): m/z 822.9017 (calcd $[(M+H)/2]^+$ = 822.8979), m/z 1644.7954 (calcd $[M]^+$ = 1644.7886). (HPLC analysis at 220 nm). Retention time on HPLC: 15.768. Peak Area on HPLC: 31193.5 mAU*s (**44 %**).

HPLC of Peptide 2d

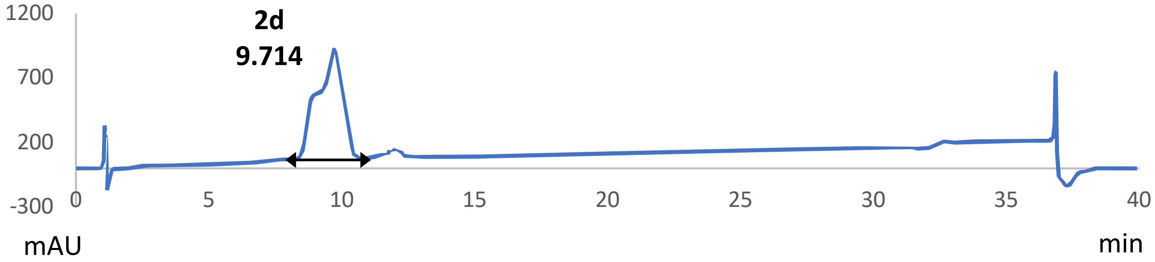

MS of Peptide 2d

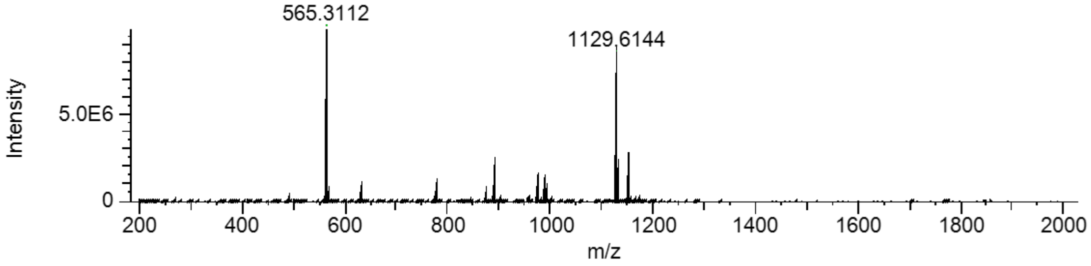

HPLC of Peptide 2d under ACA reaction and reversing condition

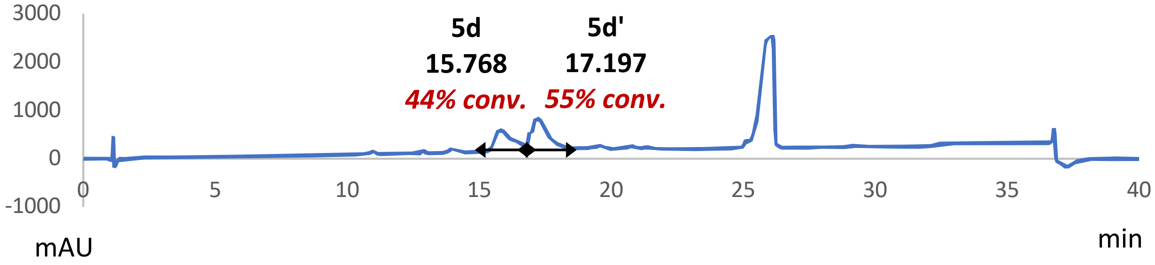

MS of Peptide 5d

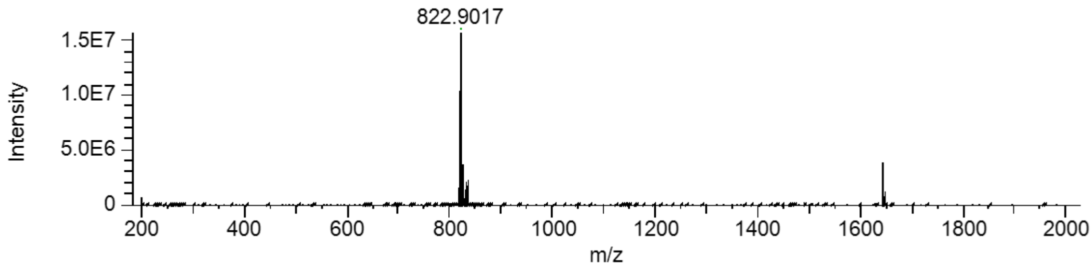

MS of Peptide 5d’

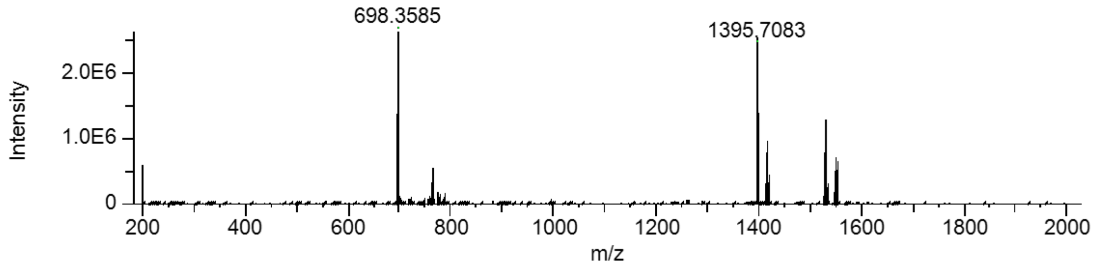

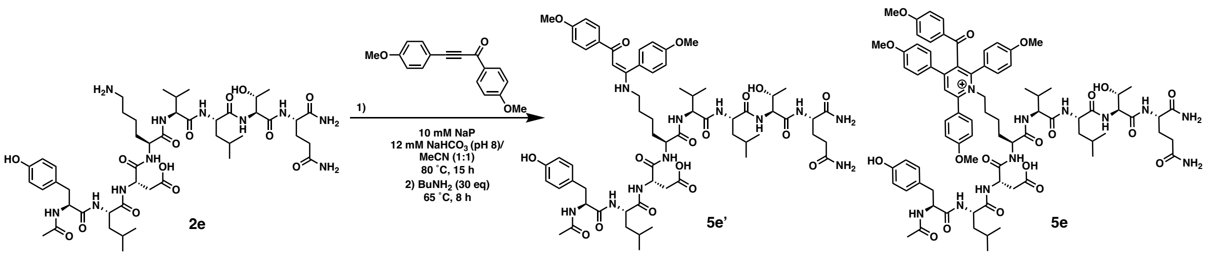

Peptide 2e. HRMS (+ESI): m/z 1020.5718 (calcd $[M+H]^+$ = 1020.5724), m/z 1042.5527 (calcd $[M+Na]^+$ = 1042.5549. (HPLC analysis at 220 nm). Retention time on HPLC: 10.418.

Peptide 5e’. HRMS (+ESI): m/z 1286.6660 (calcd $[M+H]^+$ = 1286.6667), m/z 643.8370 (calcd $[(M+H)/2]^+$ = 643.8370). (HPLC analysis at 220 nm). Retention time on HPLC: 18.495. Peak Area on HPLC: 6550.4 mAU*s (**28 %**).

Peptide 5e. HRMS (+ESI): m/z 1534.7490 (calcd $[M]^{+}$ = 1534.7504), m/z 767.8790 (calcd $[(M+H)/2]^{+}$ = 767.8789). (HPLC analysis at 220 nm). Retention time on HPLC: 17.343. Peak Area on HPLC: 16700.1 mAU*s (**71 %**).

HPLC of Peptide 2e

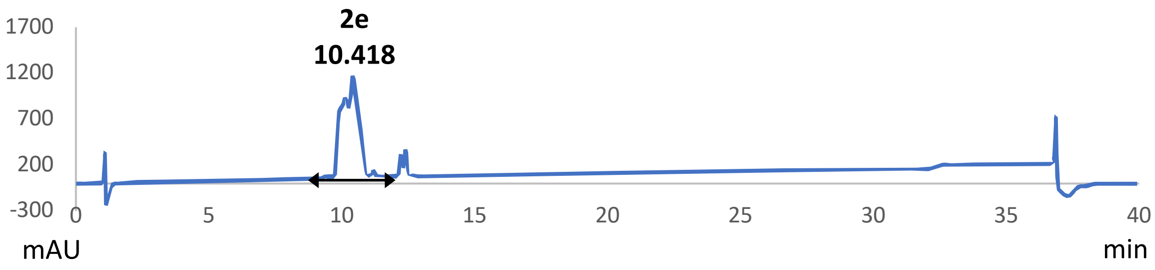

MS of Peptide 2e

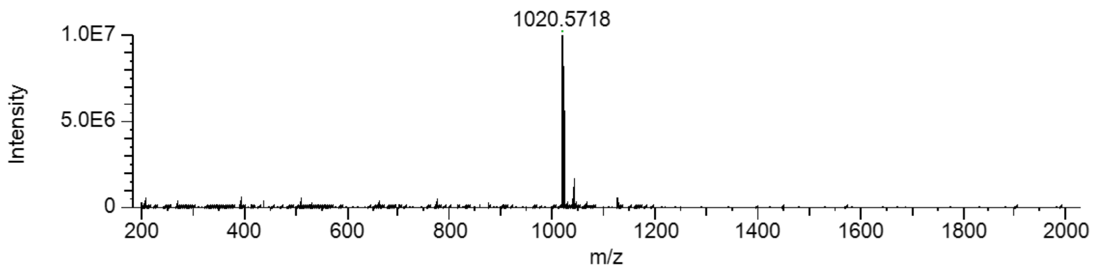

HPLC of Peptide 2e under ACA reaction and reversing condition

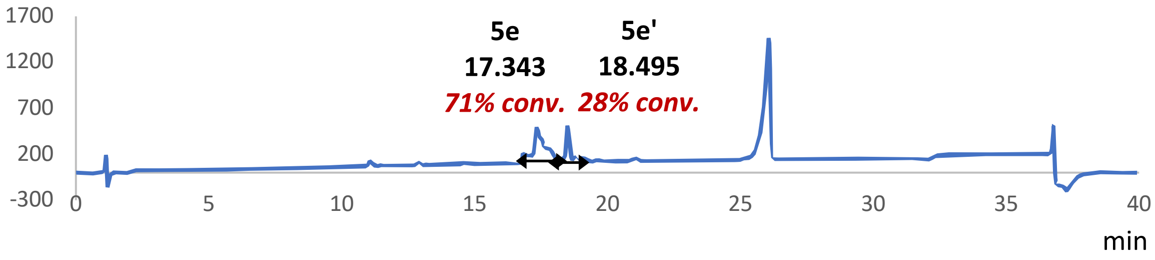

MS of Peptide 5e

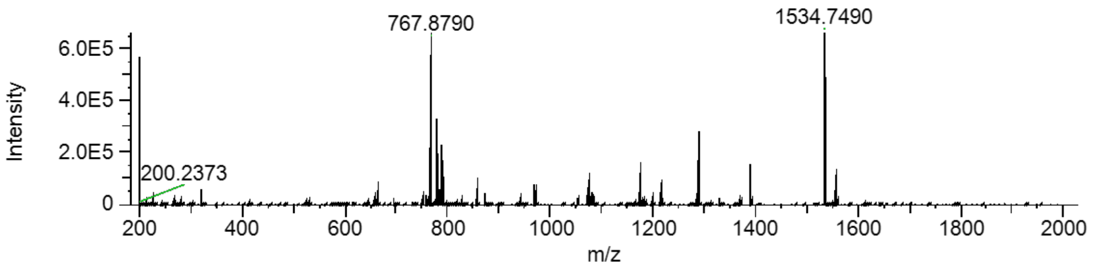

MS of Peptide 5e'

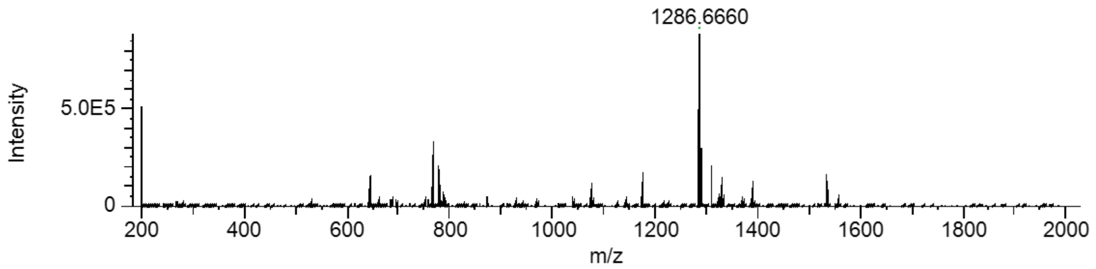

XXII. Figure 13: Synthesis, purification, and isolated yield of ACA-pyridinium peptide 5e.

First, to 3 µmol of peptide 2e, dissolved in 490 µL of solvent (6 mM), 6 mg of ynone 1f (24 µmol, 8 eq) was added. The reaction was run in a 2 mL glass vial, equipped with two stir bars. The first step was run in an oil bath at 80 ˚C and was left stirring, at 1600 rpm, for 15 h. The solvent used for the reactions is a Buffer/MeCN mixture (1:1). The buffer is 10 mM NaP/12 mM $NaHCO_3$ (pH 8). After 15 h, 9 µL of butylamine (90 µmol, 30 eq) was added to the crude reaction mixture. The reaction was stirred at 65 ˚C, 1600 rpm, for 8 h. The ACA-pyridinium peptide 5e was purified by PREP-HPLC, using the method reported in the purification section, to give 47% yield (2 mg).

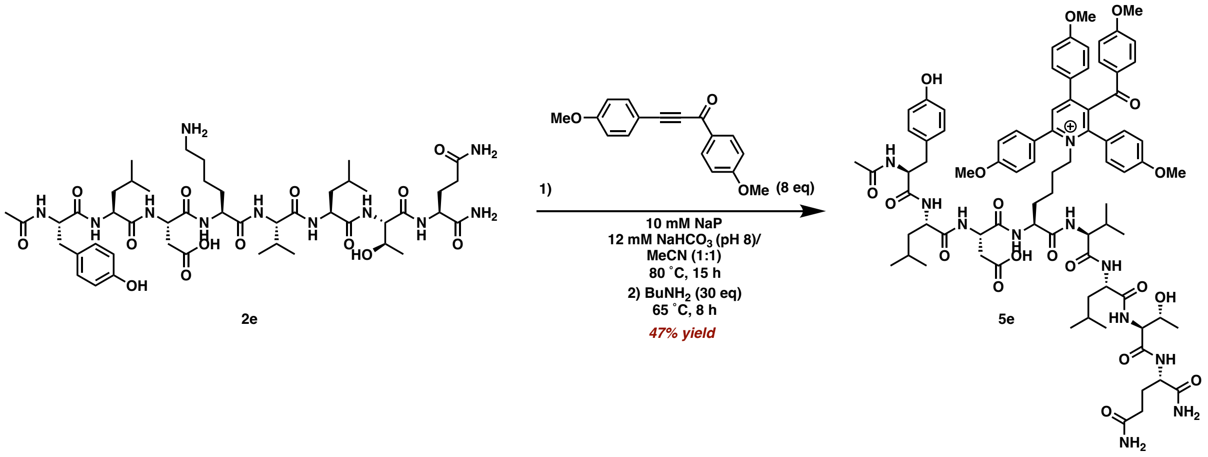

XXIII. Figure 14: Solid-support synthesis of pyridinium peptides by ACA reaction.

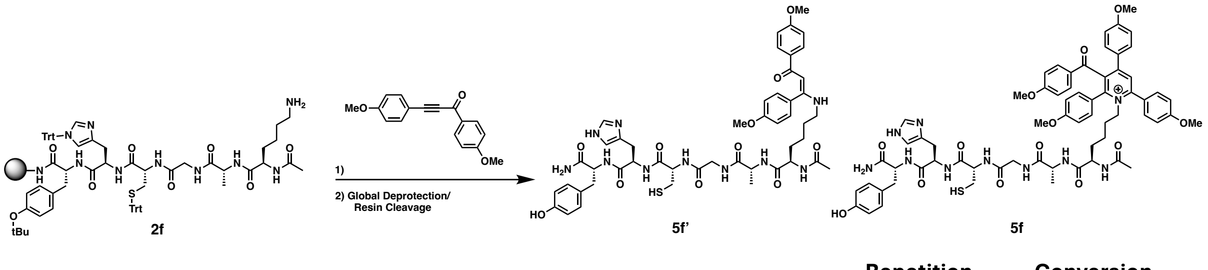

Entry	Ynone Eq.	Temp. (˚C)	Solvent/ Ratio	Time (h)	Stirring (rpm)	Repetition of step 1	Conversion of 5f' :5f (%)
1	6	90	Buffer/DMF (1:1)	15	80	n/a	71:28
2	6	90	Buffer/DMF (1:1)	15	80	1	47:52
3	6	110	Buffer/DMF (1:1)	15	0	1	71:28
4	6	65	Buffer/MeCN (1:1)	15	80	1	0:0
5[a]	6	65	H_2O/MeCN (1:1)	15	80	1	82:17

[a]Reaction was run with K_2CO_3 (1 eq).

Peptide 2f was synthesized based on the standard protocol for Fmoc-SPPS. The peptide was synthesized on a 75 µmol/395 mg scale using Fmoc-PAL-PEG-PS resin and Fmoc-L-Lys(Mtt)-OH. Lys(Mtt) was selectively deprotected using 1.8% TFA-DCM for 3 min (x10). After each deprotection step, the resin was washed with DCM, DMF, and 10% DiPEA-DMF (x2). After lysine deprotection, to 50 mg of resin (9.5 µmol of peptide), in 1585 µL of solvent (6 mM), 15 mg of ynone 1f (57 µmol, 6 eq) was added. All reactions were run in a 4 mL glass vial, equipped with a stir bar. All of the reactions were run in an oil bath, equipped with a thermometer, and were left stirring at the appropriate speed (rpm) for 15 h. For the reactions run in Buffer/MeCN, a pre-made 10 mM NaP/12 mM $NaHCO_3$ (pH 8) buffer was used. After 15 h, the peptide was deprotected, cleaved from resin, and washed with Et_2O as stated in the Fmoc-SPPS section. The crude cleavage mixture was injected into the HPLC and MS to analyze the reaction. The reaction mixture was analyzed by the HPLC method reported in the analytical method. Percent conversion was determined by calculating the area under the HPLC peaks of the cleavage mixture.

Peptide 5f'. HRMS (+ESI): m/z 985.4229 (calcd $[M+H]^+$ = 985.4236). (HPLC analysis at 220 nm). Retention time on HPLC: 19.030.

MS of Peptide 5f'

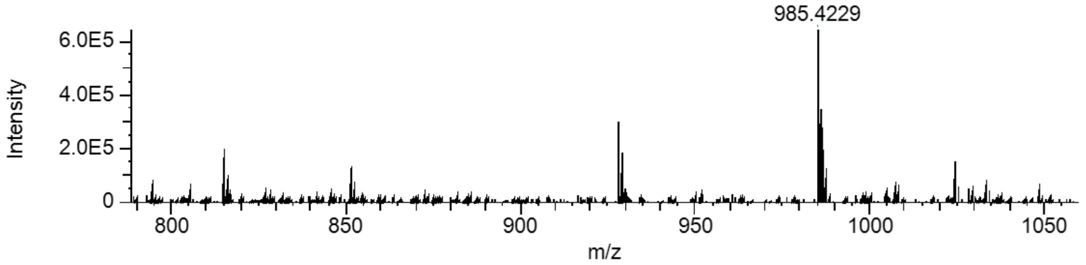

Peptide 5f. HRMS (+ESI): m/z 1233.5017 (calcd $[M]^+$ = 1233.5074), m/z 617.2572 (calcd $[(M+H)/2]^+$ = 617.2574). (HPLC analysis at 220 nm). Retention time on HPLC: 17.272.

MS of Peptide 5f

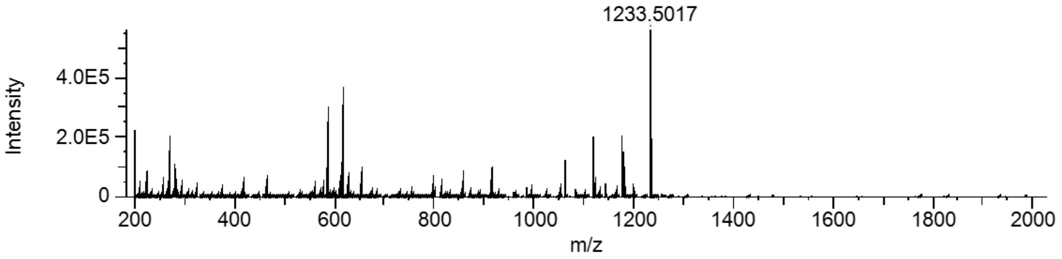

Entry 1 - Peptide 5f'. HRMS (+ESI): m/z 985.4229 (calcd $[M+H]^+$ = 985.4236). (HPLC analysis at 220 nm). Retention time on HPLC: 19.030.

Entry 1 - Peptide 5f. HRMS (+ESI): m/z 1233.5017 (calcd $[M]^+$ = 1233.5074), m/z 617.2572 (calcd $[(M+H)/2]^+$ = 617.2574). (HPLC analysis at 220 nm). Retention time on HPLC: 13.706.

HPLC of Entry 1

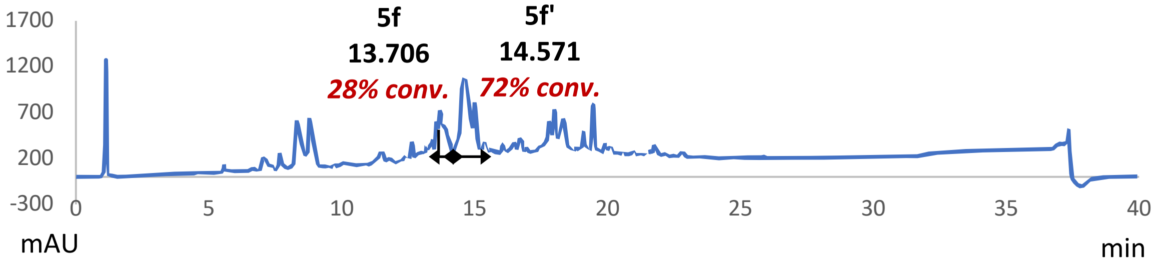

HPLC of Entry 2

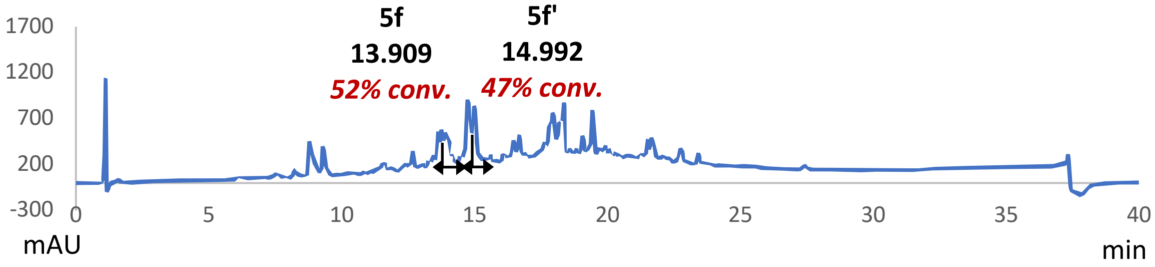

HPLC of Entry 3

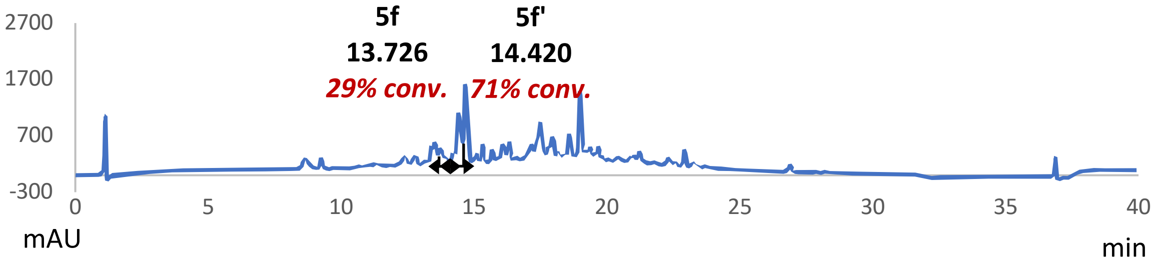

HPLC of Entry 4

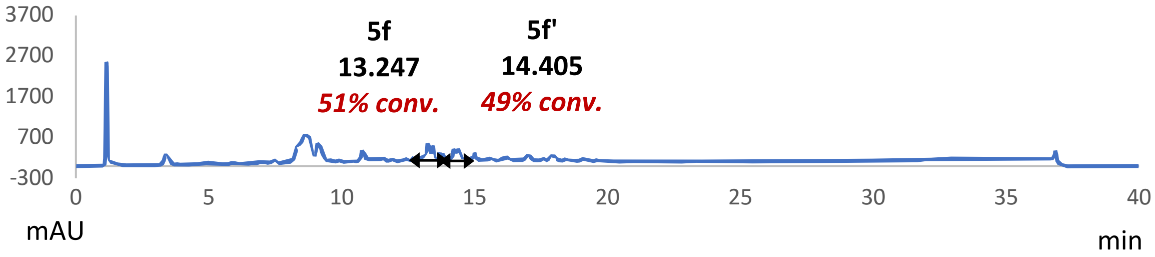

HPLC of Entry 5

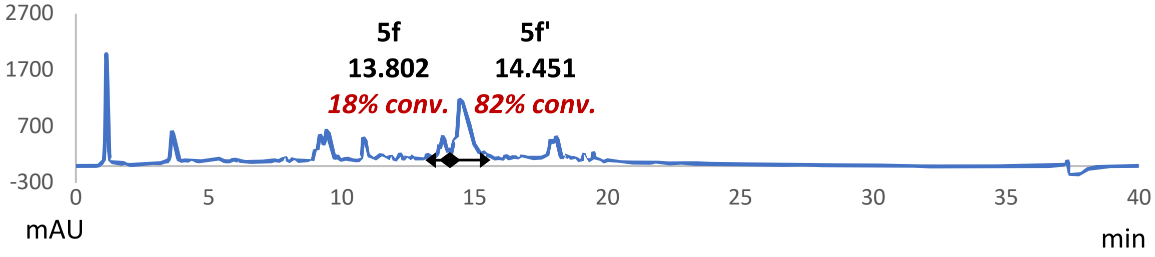

XXIV. Figure 15. Flow cytometry analysis of butyl pyridinium 4f and peptide pyridinium 5b.

Gating Strategy

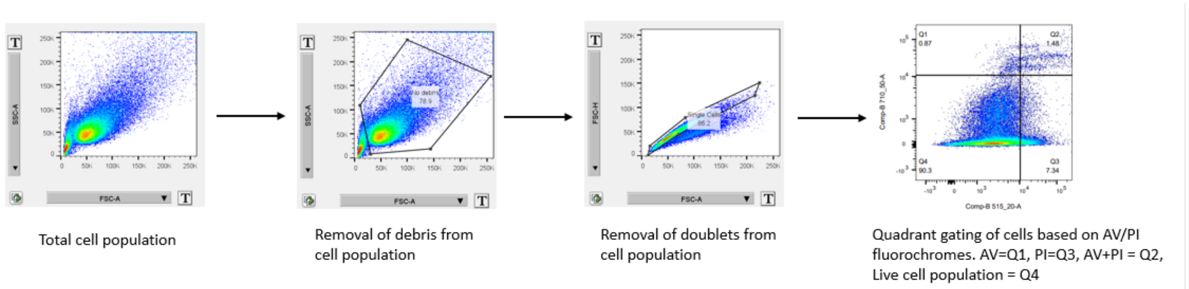

Flow Cytometry Analysis Butyl Pyridinium 4f and Peptide Pyridinium 5b

T-47D cells were treated with butyl pyridinium 4f and peptide pyridinium 5b (100 nM) individually for 1 h. The positive control for peptide 5b being the unfunctionalized peptide 2b. Peptide 5b was checked after 2 hr incubation for mitochondrial co-localisation studies. After incubation, cells were washed with PBS, detached with trypsin, and stained with Annexin V/PI, according to manufacturer's protocol. Annexin V (AV) conjugated to FITC was used to determine apoptosis and propidium iodide (PI) was used to determine necrosis within the cell population. Cells were analyzed via flow cytometry within 1 h to quantify cell death. FlowJo software (version 10.8.1) was used to analyze data collected on the cytometer. PI and AV controls were used to determine quadrant placement. All the experiments were performed duplicates (n= 3 biological replicates). Data are represented as mean ± SD.

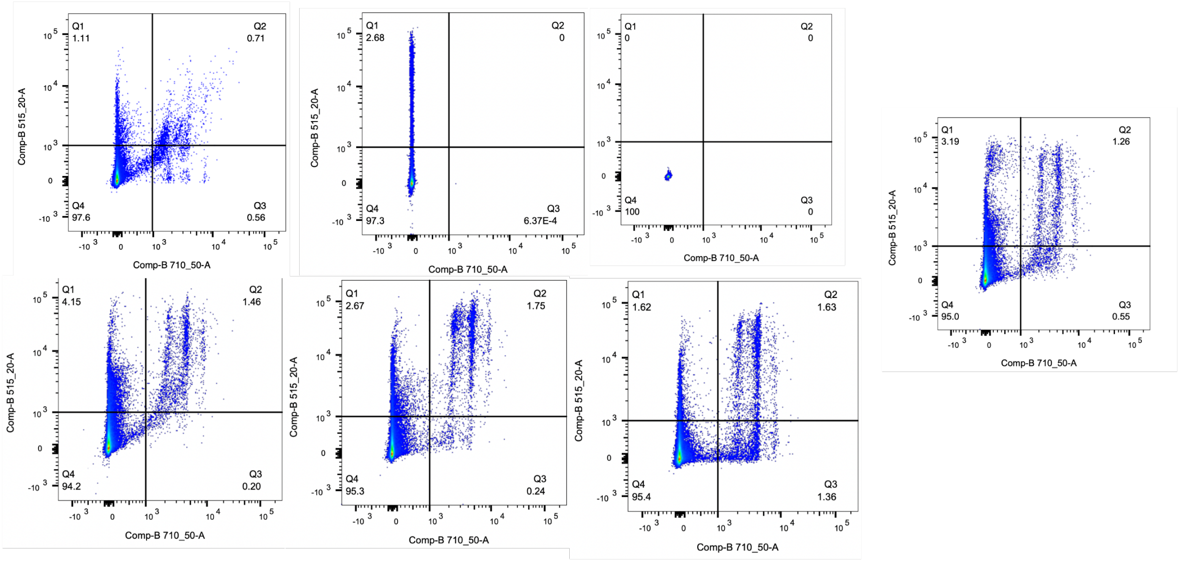

Lane1 – Left – Naive cells, Stained control, Unstained control. **Lane 2 –** Butyl pyridinium 4f, peptide 2b, peptide pyridinium 5b. Percentage Cell death of butyl pyridinium 4f and peptide pyridinium 5b were on an average 4.6%.

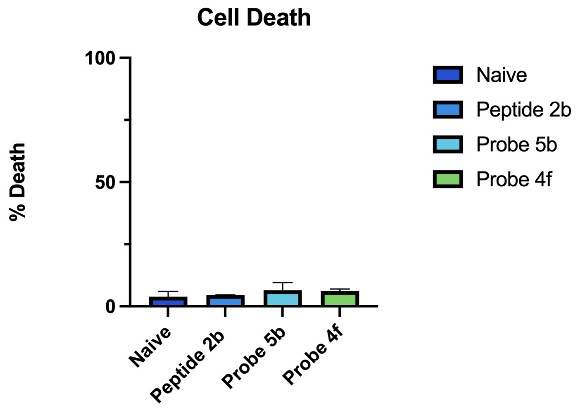

XXV. Figure 16: Imaging analysis for mitochondrial co-localization of butyl pyridinium 4f.

Live T-47D cells were plated on two 3 cm petri dishes supplemented with RPMI media and incubated for 24 h. Cells were then treated with butyl pyridinium 4f and Red-MitoTracker (100 nM) for 1 h. After 1 h, cells were washed 3 times with cold PBS. Another was kept unstained as control. The cells were then imaged in the following channels- DAPI, Furan, TRITC, Bright-field. Where Furan (380 nm) and DAPI (402 nm) correspond to the excitation wavelength of our probe and TRITC corresponds to excitation wavelength of the Red-MitoTracker, our positive control for the colocalization studies. These were initial studies done with TIRF\Epi fluorescence microscope.

Butyl Pyridinium 4f Co-localization Studies

We see >92 % overlap between the Red-MitoTracker and 4f localisation intensities.

FURAN	DAPI	TRITC	MERGED	BF

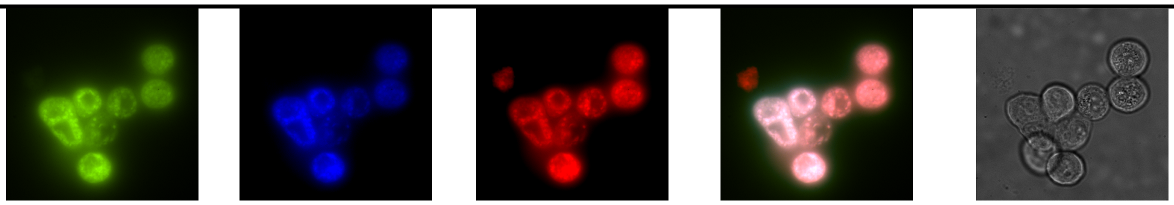

Lane 1 – Left – Images of T-47D cells incubated with Red-MitoTracker and butyl pyridinium 4f (at 380 nm, 405 nm, and 566 nm), merged images, and the last lane is bright field images. The merged images clearly show an overlap between the Red-MitoTracker and butyl pyridinium 4f.

XXVI. Figure 17: Pixel Intensity, comparison between Red-MitoTracker and Butyl Pyridinium 4f.

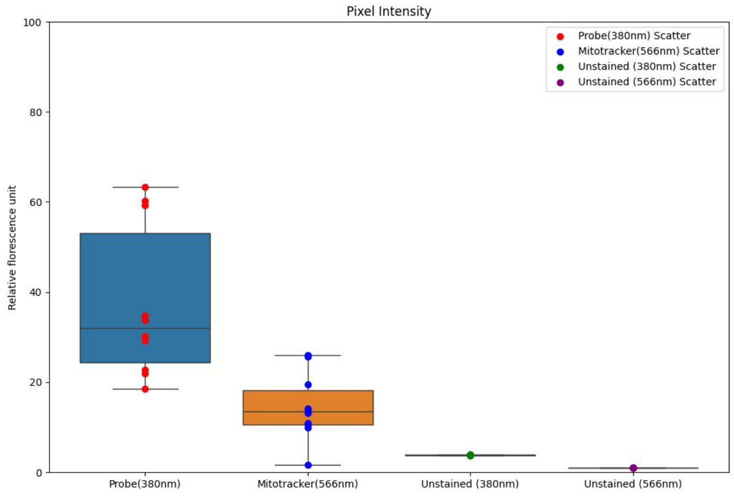

Pixel intensities were calculated in ImageJ and plotted in a jitter plot where the Y axis represents the relative fluorescence unit and X axis represents the categories. The RFU values of the probe here clearly states the higher pixel intensity of the probe is significantly higher than the unstained Furan and TRITC channels. The Pixel Intensity calculation has been derived from TIRF images of butyl pyridinium 4f, it is a representation of 15 individual cell images taken across 3 biological replicates.

XXVII. Figure 18: Imaging analysis for mitochondrial co-localization of peptide pyridinium 5b, 5c, and 5e.

Cells were plated in a glass bottom chamber in RPMI media and allowed to adhere overnight at 37 °C, 5% CO2. Fresh 10 mM stock solutions of probes were prepared in DMSO on the day of experimentation. Working solutions of all compounds in media were prepared the day of experimentation. Culture media was removed from wells and 1 mL of 100 nM probe was added to the plates and placed in an incubator for 30 minutes. Media was removed from wells and 1 mL of ACA-py peptide (100 nM) and MitoTracker-Red (100 nM) was added to desired wells and placed in an incubator for 15 minutes. Staining media was removed, and cells were washed with 4 mL of PBS for 5 minutes (repeated 3 times). PBS was removed and replaced with 1 mL of fresh media followed by immediate imaging. Five images were captured for each plate.

Protocol for Generating Scatterplot

Raw images were imported into Fiji (v1.54) and converted to 8-bit. Background was removed with a 30-pixel rolling-ball subtraction. For each cell, a region of interest encompassing the cytoplasm was drawn to exclude extracellular signal. The Co-localization Finder plugin was run with Costes' automatic

thresholding and 100 randomizations ($p < 0.01$) for each of the images across both the channels. We generated the colocalization graph. Intensity in the probe channel on the abscissa and in the reference (e.g., MitoTracker) channel on the ordinate; the overall shape therefore encodes all spatial information in a single graph. A narrow, upward-slanting plume that terminates well away from either axis, as observed here, signifies a strong positive correlation between the fluorophores, consistent with the high Pearson's coefficient ($R \approx 0.8$) reported for these data. The colour gradient (white → yellow → red → magenta → blue) reflects point density on a logarithmic scale: a bright core near the origin corresponds to low-intensity background pixels, whereas the smooth tapering of cooler hues toward higher intensities indicates a continuous, artefact-free dynamic range. Parallel streaks within the plume reveal two intensity regimes of comparable probe-to-marker ratios, suggestive of cell-to-cell variability or distinct sub-populations that may warrant further gating. The oblique white line depicts Costes automatic thresholds, ensuring that Manders overlap coefficients are calculated only for statistically significant pixels and guarding against background inflation. The absence of pronounced horizontal or vertical arms confirms minimal channel-specific bleed-through or saturation, underpinning the conclusion that the probe peptide predominantly colocalizes with mitochondria under the TIRF imaging conditions employed.

ACA-Py Peptide	Modified Peptide Sequence	Peptide Charge*	Pearson's Coefficient
5b	Ac-LDK(Py)VNR-COHN$_2$ **(6-mer)**	0	0.849
5c	Ac-VFK(Py)RN-CONH$_2$ **(5-mer)**	+1	0.899
5e	Ac-YLDK(Py)VLTQ-CONH$_2$ **(8-mer)**	-1	0.917

*Peptide charge was determined based on physiological pH (7.4). The charge does not consider the permanent positive charge observed for the pyridinium modified lysine residue.

Cellular Images of Co-localization Studies

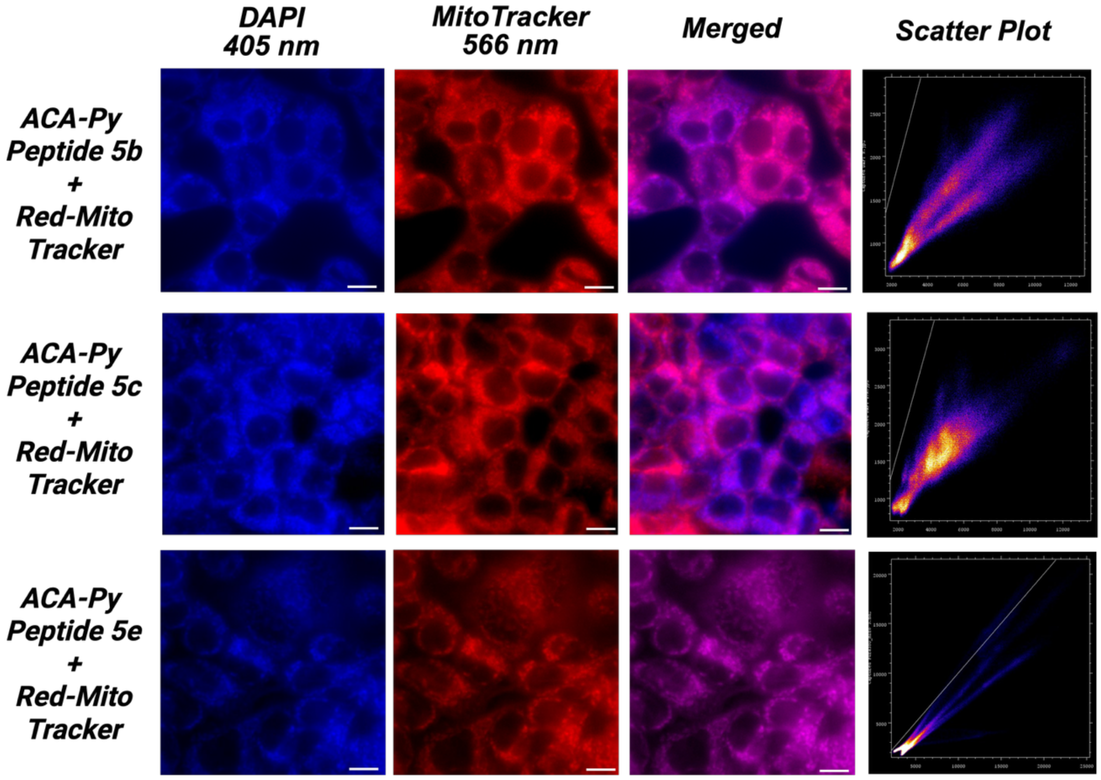

Lane 1 – DAPI (405 nm) – Images of T-47D cells incubated with Red-MitoTracker and peptide pyridinium 5b, 5c, and 5e (at 405 nm, DAPI channel). **Lane 2 – MitoTracker (566 nm) –** Images of

T-47D cells incubated with Red-MitoTracker and peptide pyridinium 5b, 5c, and 5e (at 566 nm, $\lambda_{max,ex}$ of Red-MitoTracker). **Lane 3 – Merged** – merged cellular images of Red-MitoTracker and peptide pyridiniums, taken at DAPI (405 nm) and MitoTracker (566 nm) channels. **Lane 4 – Scatter Plot** – Scatterplot of merged images, representing co-localization of Red-MitoTracker and peptide pyridiniums.

XXVIII. Figure 19: Synthesis and purification of antibacterial ACA-Py peptide 5g.

To 3.4 μmol (3 mg) of peptide 2g, dissolved in 570 μL of solvent (6 mM), 5.4 mg of ynone 1f (20 μmol, 6 eq) was added. The reaction was run in a 2 mL glass vial, equipped with two stir bars. The reaction was run in an oil bath at 80 ˚C and was left stirring, at 1600 rpm, for 15 h. The solvent used for the reactions is a Buffer/MeCN mixture (1:1). The buffer is 10 mM NaP/12 mM $NaHCO_3$ (pH 8). Samples were taken from the reaction mixture and injected into the HPLC and MS to monitor the reaction. The reaction mixture was analyzed by the HPLC method reported in the analytical method. The ACA-pyridinium peptide 5g was purified by PREP-HPLC, using the method reported in the purification section, to give 26% yield (1.2 mg).

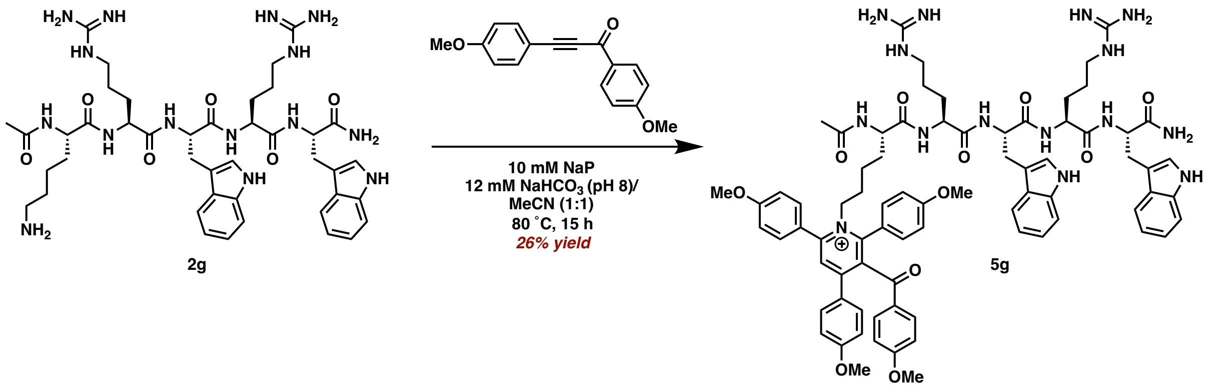

Peptide 2g. HRMS (+ESI): m/z 872.5001 (calcd $[M+H]^+$ = 872.5002), m/z 436.7537 (calcd $[(M+2H)/2]^+$ = 436.7538). (HPLC analysis at 220 nm). Retention time on HPLC: 21.742. HPLC Method: 0-50% MeCN/H_2O (0.1% FA) in 30 min.

Peptide 5g. HRMS (+ESI): m/z 1386.6777 (calcd $[M]^+$ = 1386.6782), m/z 693.8426 (calcd $[(M+H)/2]^+$ = 693.8428), m/z 462.8976 (calcd $[(M+2H)/3]^+$ = 462.8976). (HPLC analysis at 220 nm). Retention time on HPLC: 14.776.

HPLC of Peptide 2g

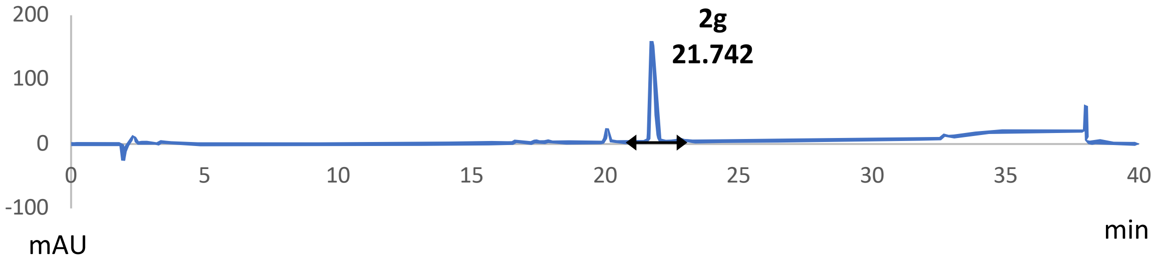

MS of Peptide 2g

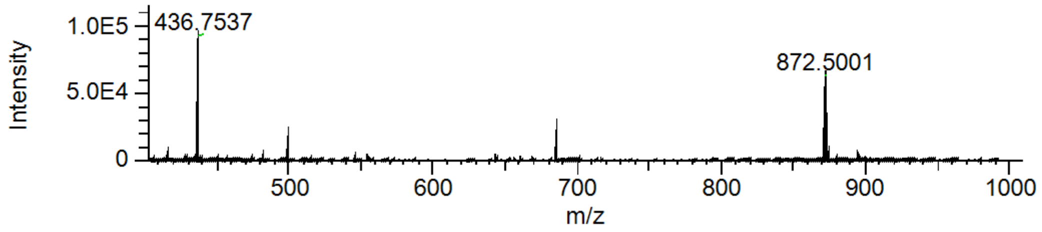

HPLC of Peptide 2g under ACA reaction

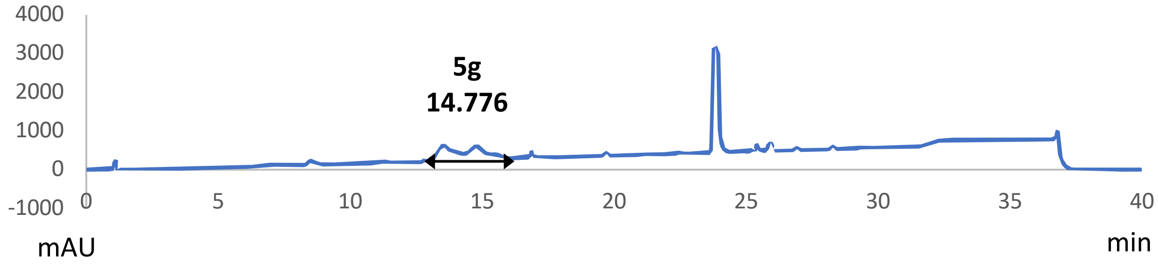

MS of Peptide 5g

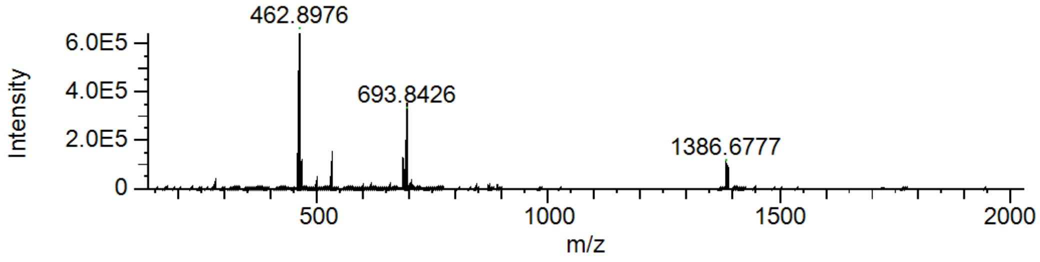

XXIX. Figure 20: Synthesis and purification of ACA-Py peptide 5a.

To 3.9 µmol (3 mg) of peptide 2a, dissolved in 648 µL of solvent (6 mM), 6.2 mg of ynone 1f (23 µmol, 6 eq) was added. The reaction was run in a 2 mL glass vial, equipped with two stir bars. The reaction was run in an oil bath at 80 ˚C and was left stirring, at 1600 rpm, for 15 h. The solvent used for the reactions is a Buffer/MeCN mixture (1:1). The buffer is 10 mM NaP/12 mM $NaHCO_3$ (pH 8). Samples were taken from the reaction mixture and injected into the HPLC and MS to monitor the reaction. The reaction mixture was analyzed by the HPLC method reported in the analytical method. The ACA-pyridinium peptide 5a was purified by PREP-HPLC, using the method reported in the purification section, to give 22% yield (1 mg).

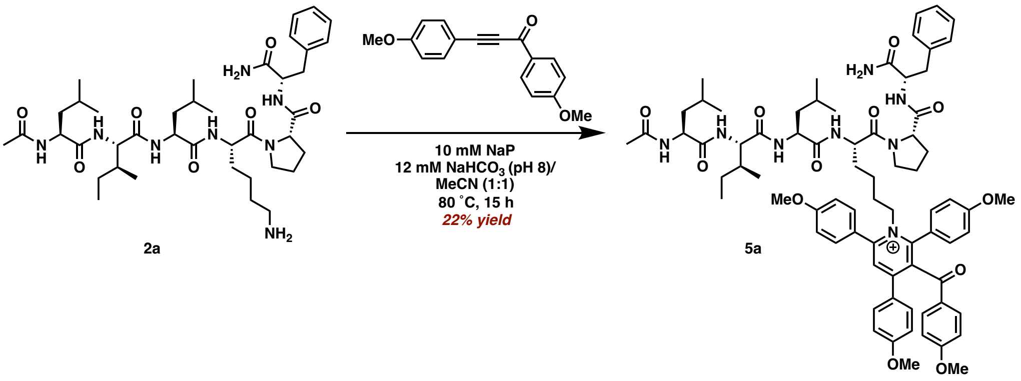

XXX. Figure 21. Antibacterial potency of ACA-Py peptide 5g (MIC assay, *E. coli*).

Minimum-Inhibitory-Concentration (MIC) Assay

MIC values for the ACA-modified antibacterial peptide 5g and its parent peptide 2g were determined by the broth-microdilution method following CLSI M07-A10 guidelines. Briefly, each compound was dissolved in Mueller–Hinton broth (MHB) at 2 mM, then dispensed into column 11 of a sterile 96-well plate and serially two-fold diluted across the plate to give a final range of 1.0 mM to 1.95 µM (11 concentrations). Wells in column 12 contained sterile medium (blank) and column 1 contained bacteria without peptide (growth control). A standardized inoculum of *E. coli* (≈ 5 × 10^5 CFU mL^{-1}) in MHB was added (50 µL per well) giving a total volume of 100 µL per well. Plates were sealed and incubated at 37 °C for 18 h, after which OD_{600} was recorded on a plate reader.

Data Analysis and MIC Definition

Raw OD values were blank-subtracted and normalized to the mean growth control in GraphPad Prism 10. A four-parameter logistic (variable-slope) fit of log_{10}[peptide] versus % growth was applied; MIC was taken as the concentration corresponding to 10 % growth (inverse prediction). The traditional (discrete) MIC was defined as the lowest tested concentration with ≤ 10 % growth relative to control.

Under these conditions the discrete MIC was 0.125 mM for both peptides, while curve fitting gave interpolated MIC values of 0.0715 mM (modified peptide) and 0.0693 mM (parent peptide).

Microdilution Plate Layout Showing Serial Two-Fold Dilutions for MIC Determination

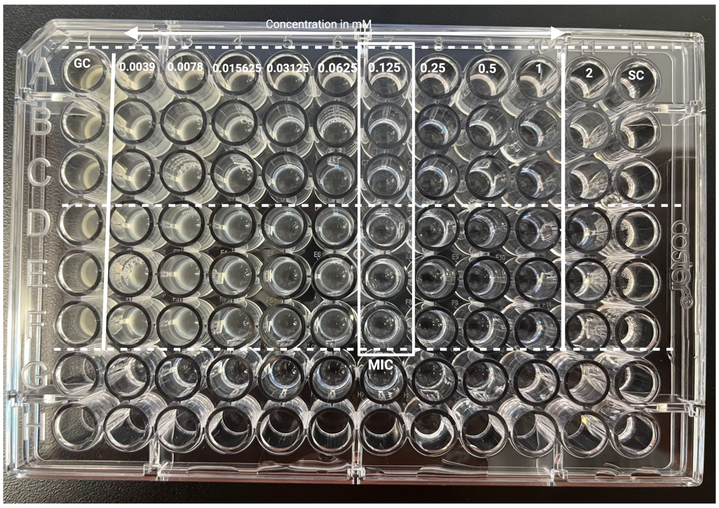

The figure displays a 96-well microplate used for MIC assay with concentrations of the tested compound ranging from 0.0039 mM to 2 mM across columns. Column 1 (GC) represents the growth control, while column 12 (SC) is the sterility control. The minimum inhibitory concentration (MIC) was identified as the lowest concentration with no visible bacterial growth (highlighted).

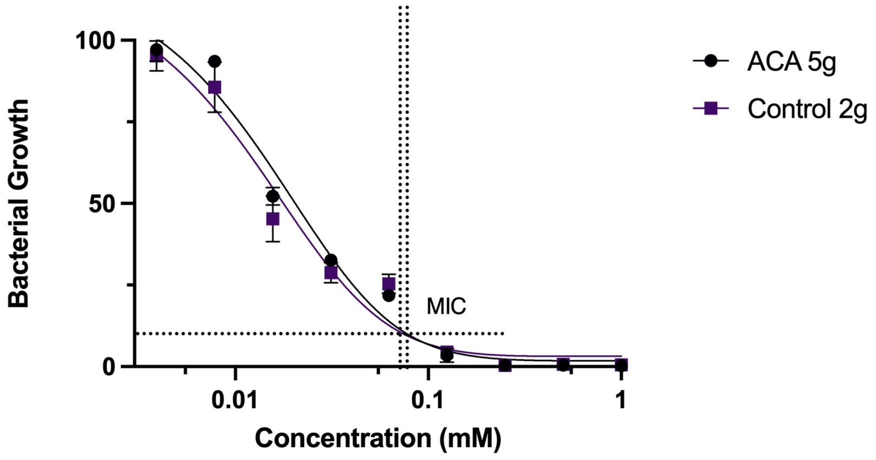

Metric	Parent Peptide (2g)	ACA-Py peptide (5g)
**Discrete-well MIC** (CLSI definition, two-fold dilutions)	0.125 mM	0.125 mM
**Extrapolated MIC** (4-parameter logistic fit)	0.069 ± 0.06 mM	0.072 ± 0.01 mM

Interpretation – The ACA-modified peptide (5g) shows an MIC indistinguishable (≤ 5 % difference) from the parent, indicating full retention of antibacterial potency.

XXXI. Figure 22. Mammalian cytotoxicity of ACA-Py peptide 5a (Annexin V / PI flow cytometry).

Human breast-carcinoma T-47D cells were seeded in 6 cm dishes (1 × 10^6 cells $dish^{-1}$) and allowed to adhere overnight in DMEM supplemented with 10 % FBS and 1 % penicillin–streptomycin. The following day, the medium was replaced with serum-free DMEM containing either buffer alone (naïve control), the parent peptide 2a, or the ACA-modified peptide 5a (each 10 µM; total volume = 4 mL). After 2 h, at 37 °C, cells and supernatants were harvested, washed once with ice-cold PBS, and resuspended in 100 µL 1 × Annexin V binding buffer. Suspensions were stained for 15 min at room temperature with Annexin V-FITC (5 µL) and propidium iodide (PI, 5 µg mL^{-1}), diluted to 500 µL with binding buffer, and analyzed immediately on a BD FACSymphony (2000000 events per sample). Data was processed in FlowJo v10; double-positive (Annexin V^+/PI^+) events were taken as dead cells and expressed as % death (mean ± SD, n = 3). Under these conditions buffer-treated T-47D cells showed ≤ 5 % death, whereas peptides 2a and 5a produced 78 ± 3 % and 82 ± 5 % death, respectively.

Condition (10 µM, 2 h)	% Dead cells (Annexin V^+/PI^+, *n* = 3)
Buffer control	4 ± 1 %
Parent Peptide (2g)	78 ± 3 %
ACA-Py peptide (5g)	82 ± 5 %

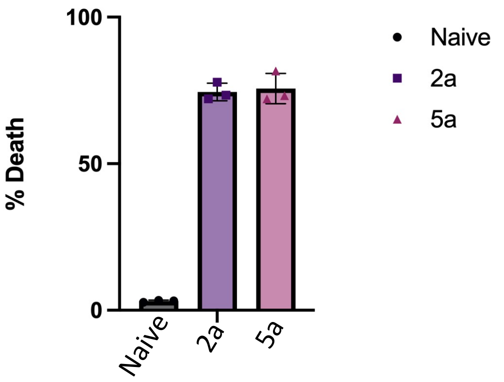

Interpretation – Both peptides elicit comparable cell death (no statistical difference, $P > 0.05$), confirming that ACA-modification does not abolish cytotoxic activity.

XXXII. Figure 23: Synthesis of Doxorubicin pyridinium (DOX-Py).

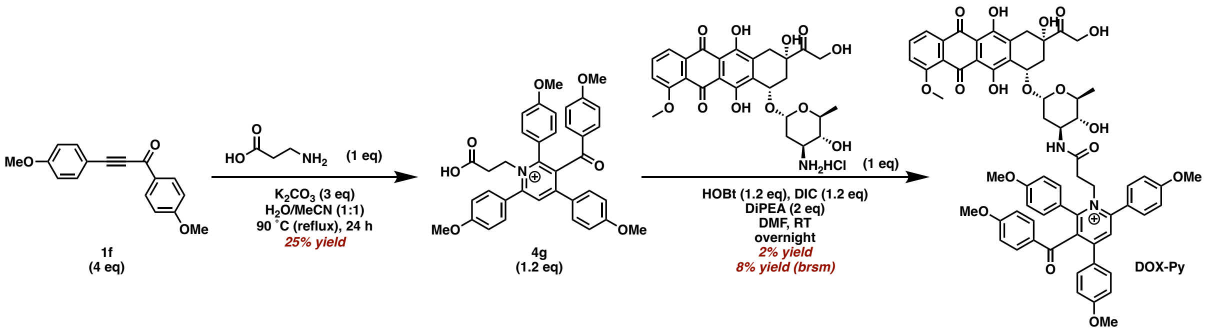

1-(2-carboxyethyl)-3-(4-methoxybenzoyl)-2,4,6-tris(4-methoxyphenyl)pyridin-1-ium (4g)

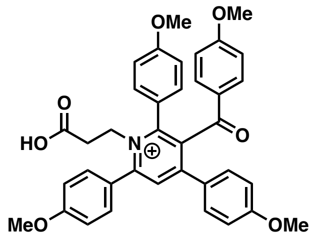

To a 25 mL round-bottom flask (equipped with a stir bar), ynone 1f (1.5 mmol, 4 eq), dissolved in 5 mL of MeCN, and beta-alanine (0.38 mmol, 1 eq) were added. Potassium carbonate (1.1 mmol, 3 eq), dissolved in 5 mL of deionized water, was then added. The reaction was run in an oil bath and under reflux, heated to 90 ˚C. The reaction was left stirring for 24 h, at 1590 rpm. After 24 h, the solvent was concentrated, and the crude reaction was purified through PREP-HPLC using the method reported in the purification section. The PREP-HPLC fractions were concentrated and lyophilized. This compound was isolated as a yellow-brown solid with 25% yield.

^{1}H NMR (400 MHz, CDCl$_3$) δ 12.91 (s, 1H), 8.52 (s, 1H), 7.69 (s, 1H), 7.64 – 7.59 (m, 2H), 7.56 (d, J = 8.6 Hz, 2H), 7.40 (d, J = 8.8 Hz, 2H), 7.35 (dd, J = 8.6, 2.2 Hz, 1H), 7.28 – 7.23 (m, 1H), 7.02 (d, J = 8.4 Hz, 2H), 6.89 (dd, J = 8.6, 2.6 Hz, 1H), 6.76 (d, J = 8.8 Hz, 2H), 6.70 (d, J = 8.7 Hz, 2H), 6.59 (dd, J = 8.6, 2.6 Hz, 1H), 4.67 – 4.59 (m, 2H), 3.81 (s, 3H), 3.71 (s, 3H), 3.68 (d, J = 5.5 Hz, 6H), 2.37 (t, J = 7.4 Hz, 2H). **^{13}C NMR (101 MHz, CDCl$_3$)** δ 190.90, 164.63, 161.85, 161.79, 161.26, 156.60, 154.59, 154.45, 138.34, 132.36, 131.68, 130.96, 130.88, 130.62, 129.98, 128.87, 126.66, 124.71, 121.68, 115.05, 114.70, 114.30, 55.62, 55.57, 55.40, 55.31. **HRMS (+ESI):** m/z 604.2322 (calcd $[M]^+$ = 604.2330).

MS of 4g

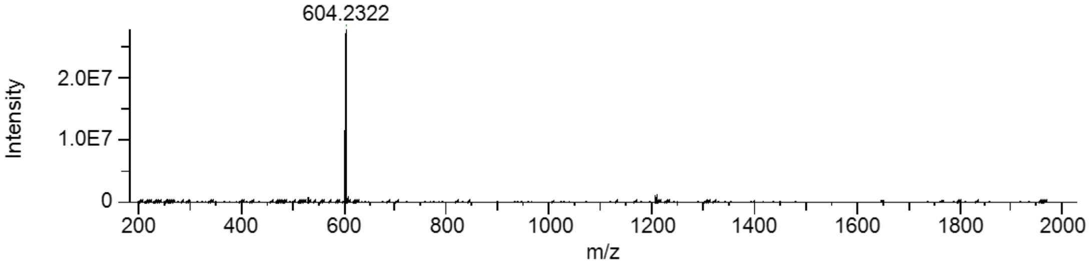

1-(3-(((2*S*,3*R*,4*S*,6*R*)-3-hydroxy-2-methyl-6-(((1*S*,3*S*)-3,5,12-trihydroxy-3-(2-hydroxyacetyl)-10-methoxy-6,11-dioxo-1,2,3,4,6,11-hexahydrotetracen-1-yl)oxy)tetrahydro-2*H*-pyran-4-yl)amino)-3-oxopropyl)-3-(4-methoxybenzoyl)-2,4,6-tris(4-methoxyphenyl)pyridin-1-ium (DOX-Py)

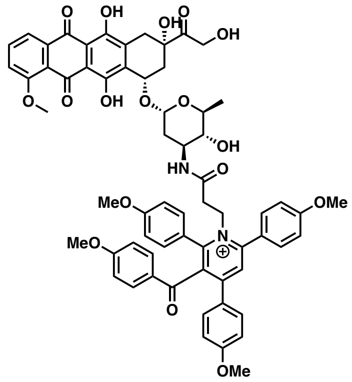

To a 10 mL round-bottom flask (equipped with a stir bar), pyridinium 4h (0.09 mmol, 1.2 eq), dissolved in 1 mL of DMF, and Doxorubicin hydrochloride (0.08 mmol, 1 eq) were added. HOBt (0.09 mmol, 1.2 eq), DIC (0.09 mmol, 1.2 eq), and DiPEA (0.15 mmol, 2 eq), all dissolved in 1 mL of DMF, were then added. The reaction was left stirring overnight, at room temperature. Reaction progress was analyzed by HPLC. The crude reaction was then purified by PREP-HPLC using the method reported in the purification section. The PREP-HPLC fractions were concentrated and lyophilized. This compound was isolated as an orange-brown solid with 2% yield. During purification, 0.06 mmol of pyridinium 4h was recovered to give 8% yield (based on recovered starting material).

HRMS (+ESI): m/z 1129.3960 (calcd $[M]^+$ = 1129.3965).

MS of DOX-Py

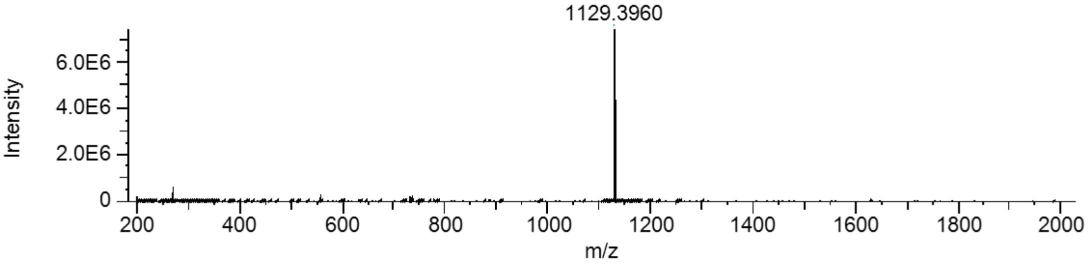

XXXIII. Figure 24: Fluorescent properties of DOX-Py.

Fluorescence of DOX and DOX-Py was measured at λ_{ex}= 480 nm, in 1:1 DMSO/water (50 µM).

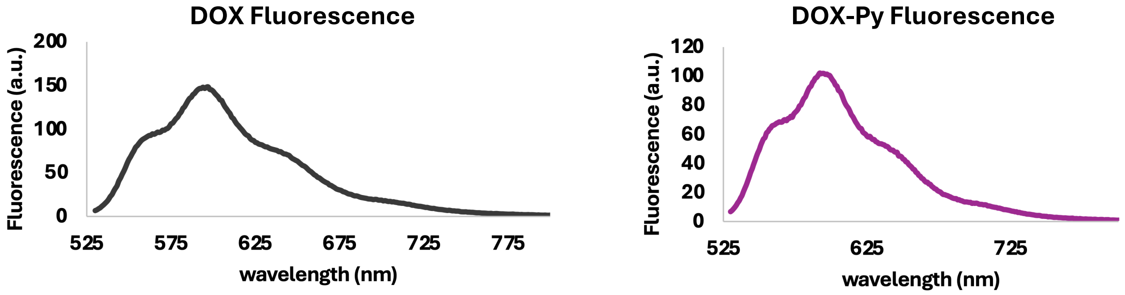

XXXIV. Figure 25: Flow cytometry analysis of Doxorubicin and DOX-Py.

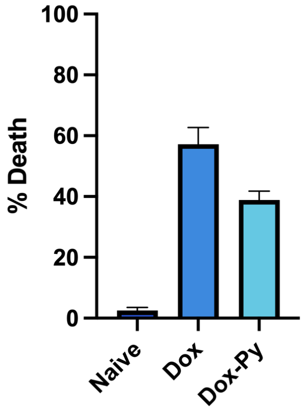

T-47D cells were treated with Doxorubicin and DOX-Py (100 nM) individually for 1 h. Untreated naive cells were used as control. After incubation, cells were washed with PBS, detached with trypsin, and stained with Annexin V/PI, according to manufacturer's protocol. Annexin V (AV) conjugated to FITC was used to determine apoptosis and propidium iodide (PI) was used to determine necrosis within the cell population. Cells were analyzed via flow cytometry within 1 h to quantify cell death. FlowJo software (version 10.8.1) was used to analyze data collected on the cytometer. PI and AV controls were used to determine quadrant placement. All the experiments were performed triplicates (n= 3 biological replicates). Data are represented as mean ± SD.

The cells treated with Doxorubicin had 20% less survival than DOX-Py. The Naive untreated cells were 97%.

XXXV. Figure 26: ^{1}H, ^{19}F, and ^{13}C-NMR spectra of synthesized compounds

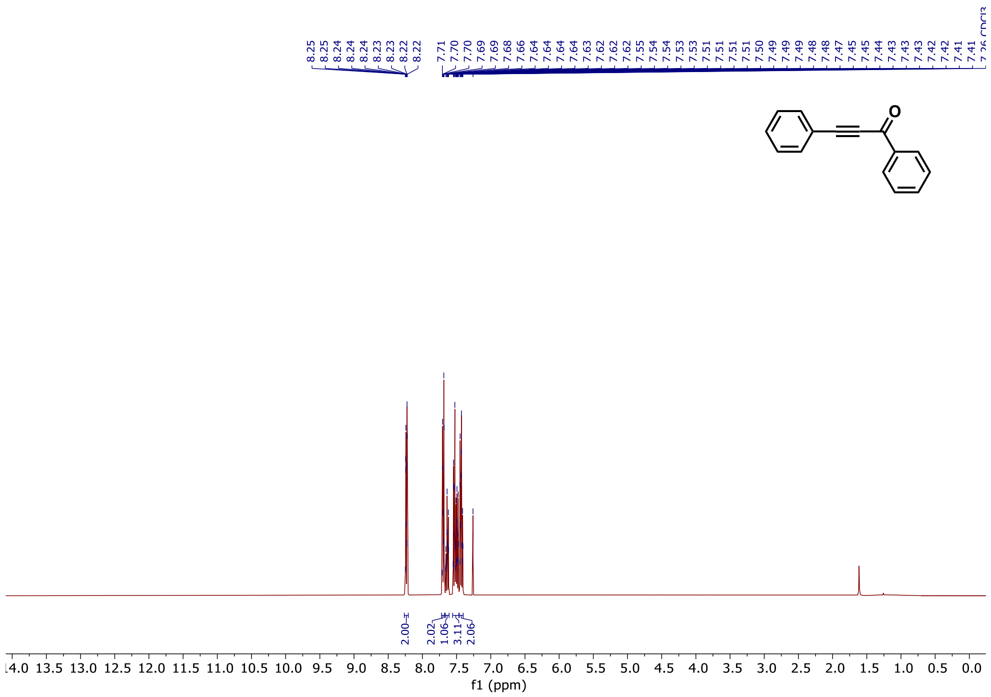

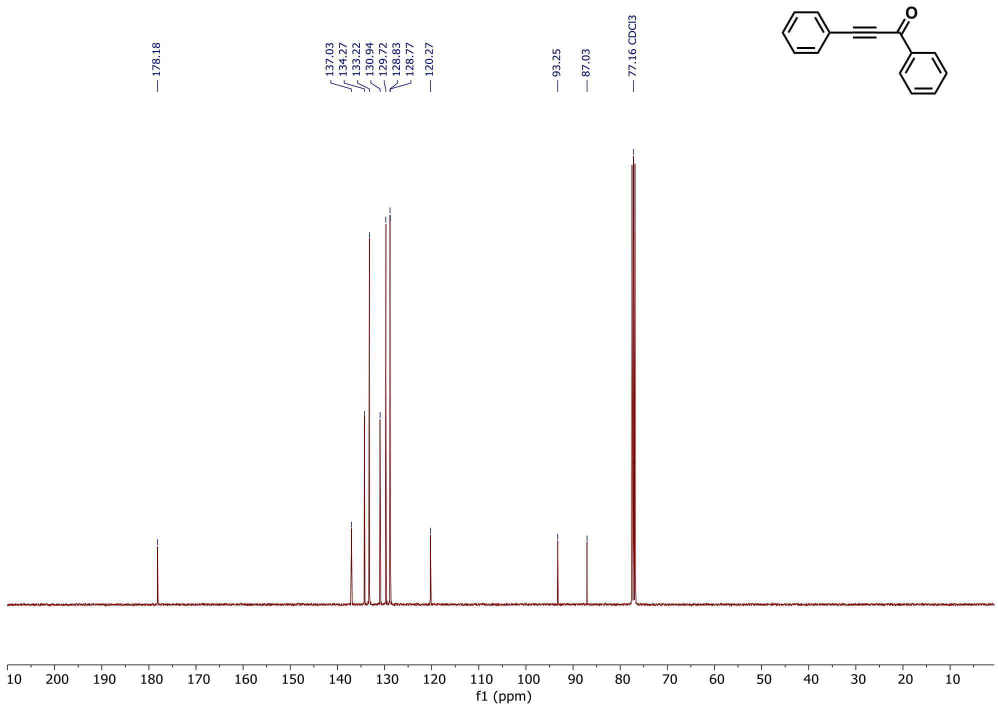
178.18
137.03
134.27
133.22
130.94
129.72
128.83
128.77
120.27
93.25
87.03
77.16 CDCl3
O
f1 (ppm)

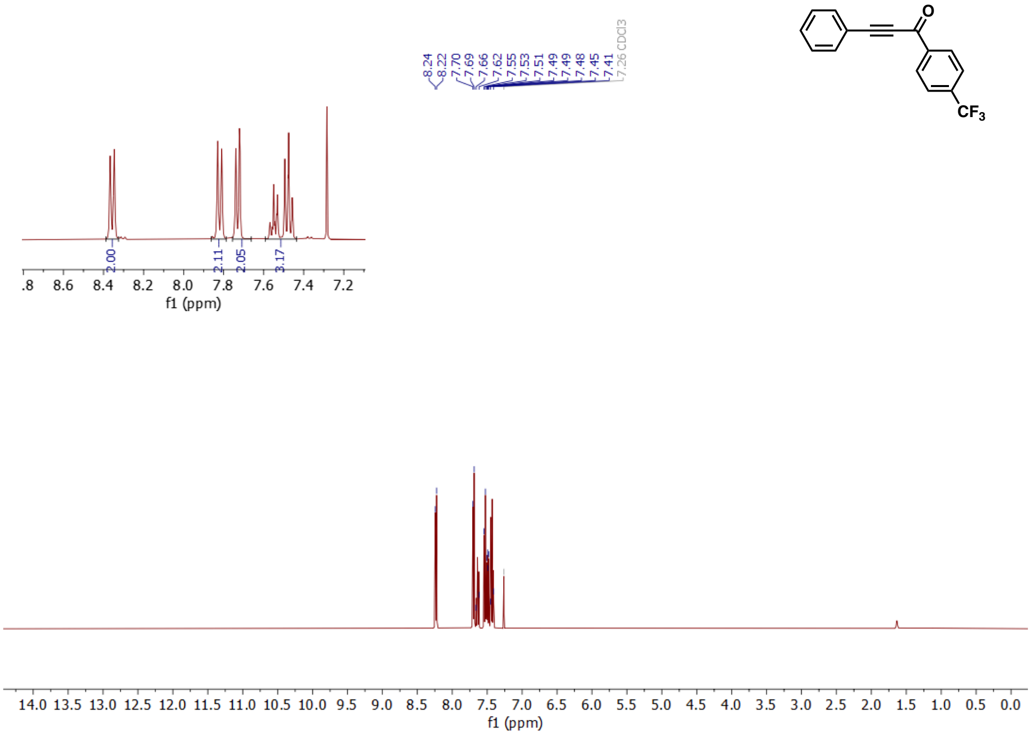
8.24
8.22
7.70
7.69
7.66
7.62
7.55
7.53
7.51
7.49
7.49
7.48
7.45
7.41
7.26 CDCl3
O
CF_3
2.00
2.11
2.05
3.17
f1 (ppm)
f1 (ppm)

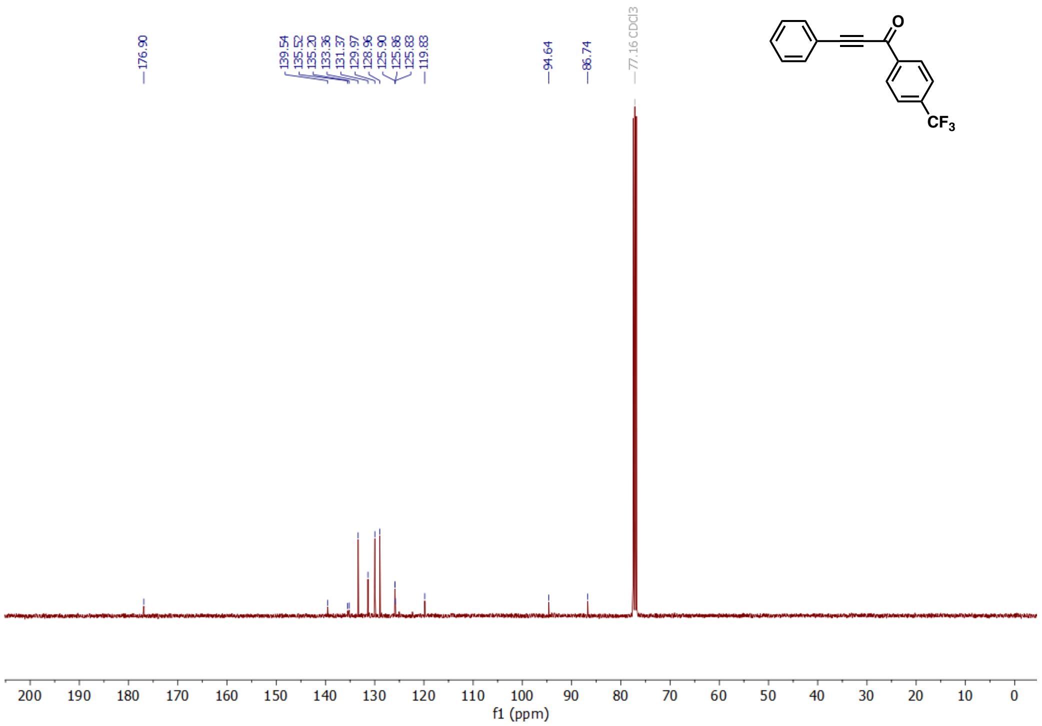
176.90
139.54
135.52
135.20
133.36
131.37
129.97
128.96
125.90
125.86
125.83
119.83
94.64
86.74
77.16 CDCl3
O
CF3
200 190 180 170 160 150 140 130 120 110 100 90 80 70 60 50 40 30 20 10 0
f1 (ppm)

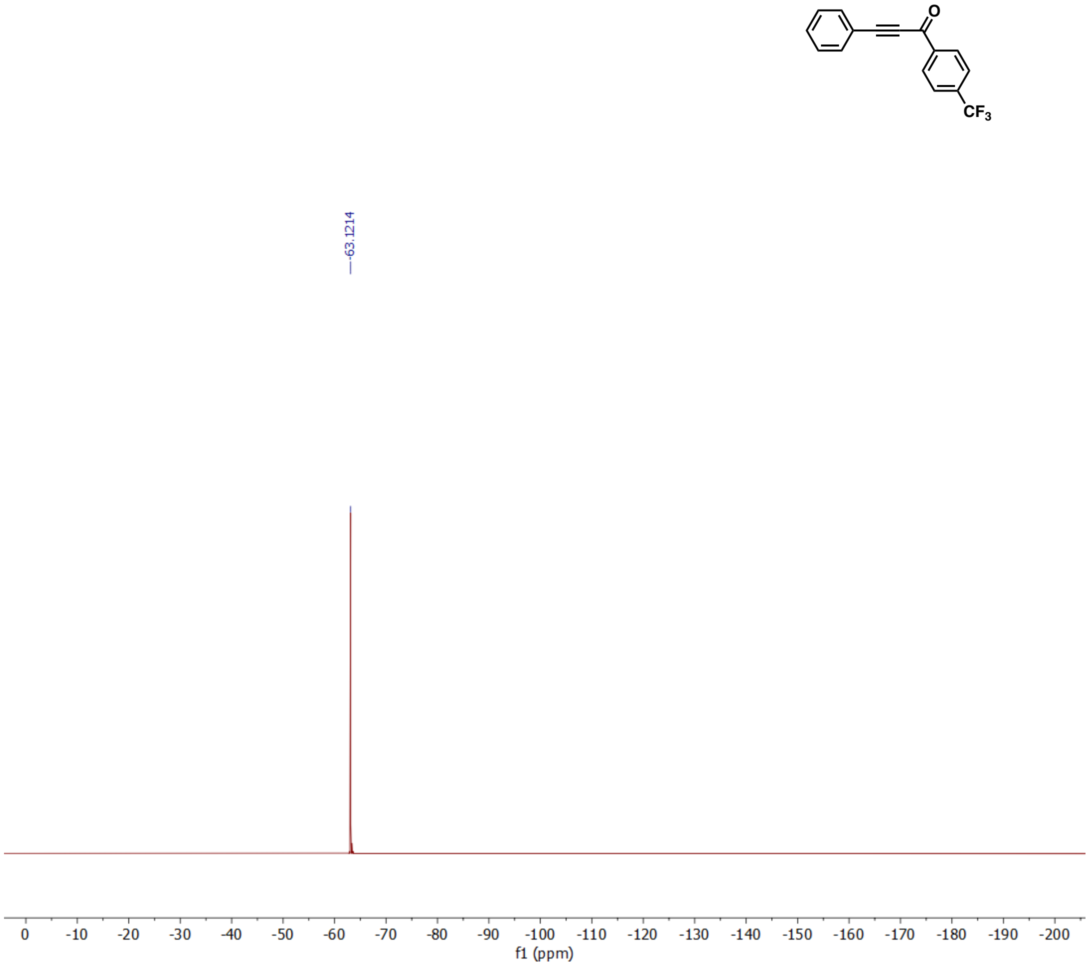
O
CF3
-63.1214
0 -10 -20 -30 -40 -50 -60 -70 -80 -90 -100 -110 -120 -130 -140 -150 -160 -170 -180 -190 -200
f1 (ppm)

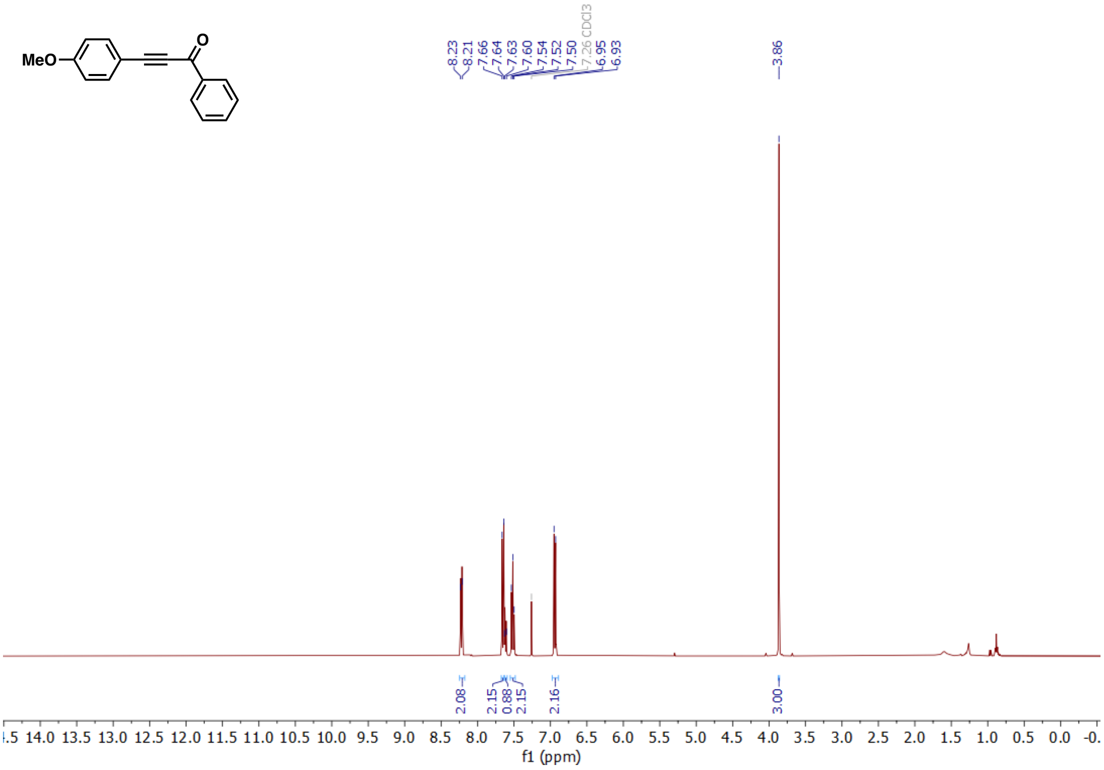
MeO
O
8.23
8.21
7.66
7.64
7.63
7.60
7.54
7.52
7.50
7.26 CDCl3
6.95
6.93
3.86
2.08
2.15
0.88
2.15
2.16
3.00
f1 (ppm)

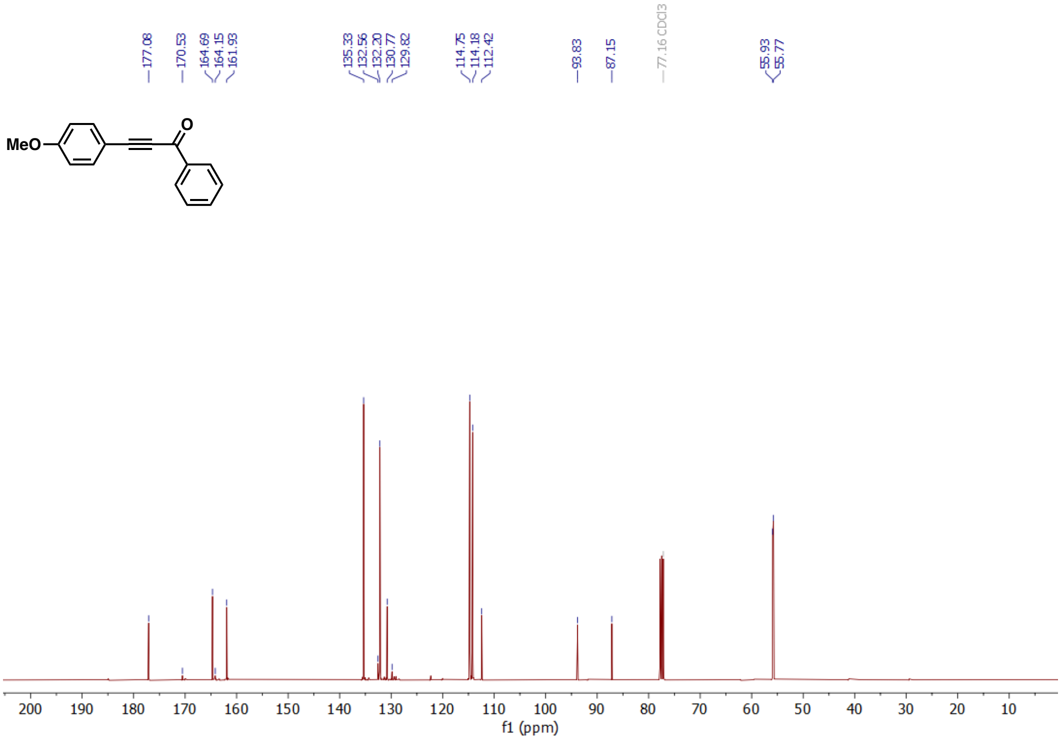
177.08
170.53
164.69
164.15
161.93
135.33
132.56
132.20
130.77
129.82
114.75
114.18
112.42
93.83
87.15
77.16 CDCl3
55.93
55.77
MeO
O
f1 (ppm)

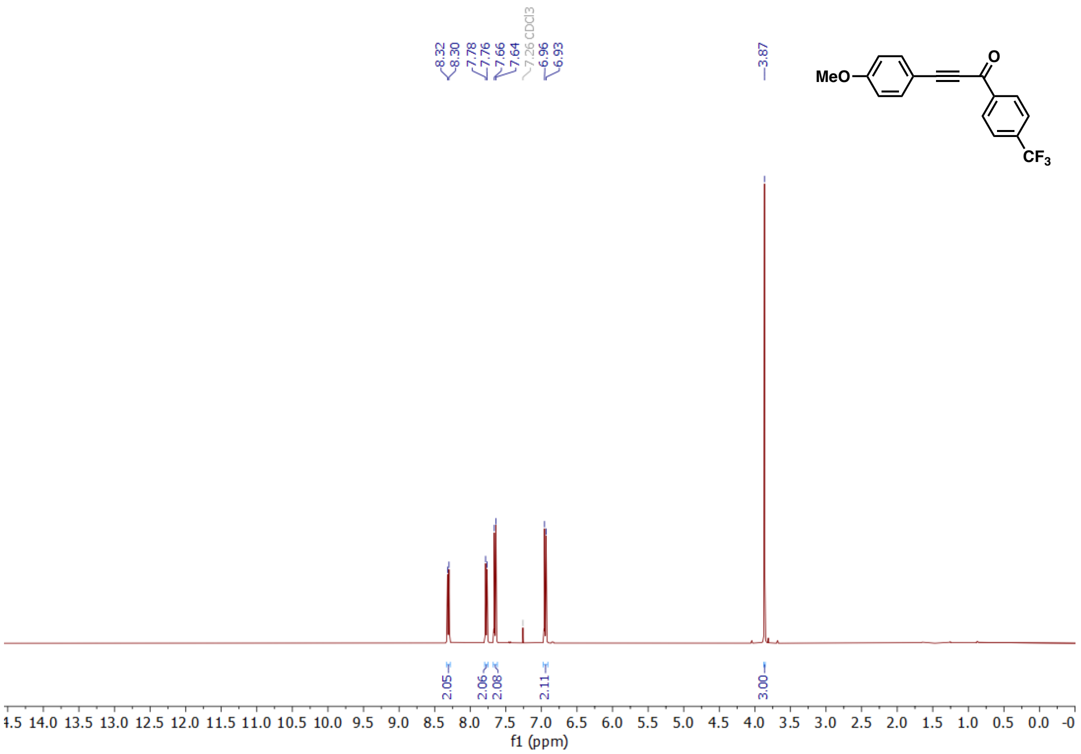
8.32
8.30
7.78
7.76
7.66
7.64
7.26 CDCl3
6.96
6.93
3.87
MeO
O
CF3
2.05
2.06
2.08
2.11
3.00
f1 (ppm)

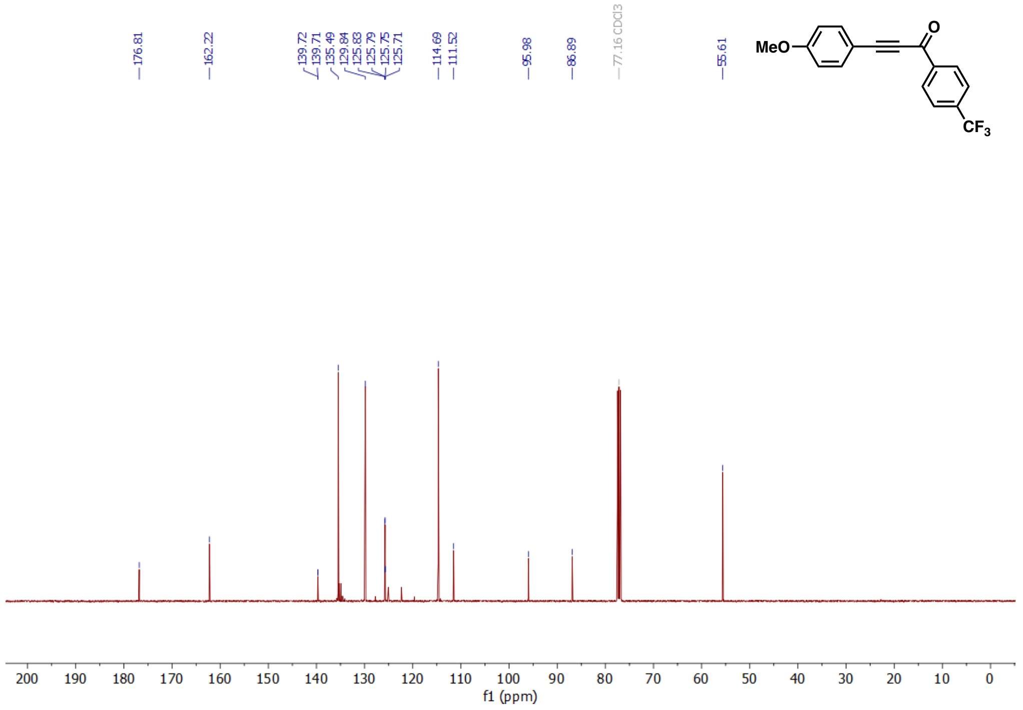
176.81
162.22
139.72
139.71
135.49
129.84
125.83
125.79
125.75
125.71
114.69
111.52
95.98
86.89
77.16 CDCl3
55.61
MeO
O
CF3
f1 (ppm)

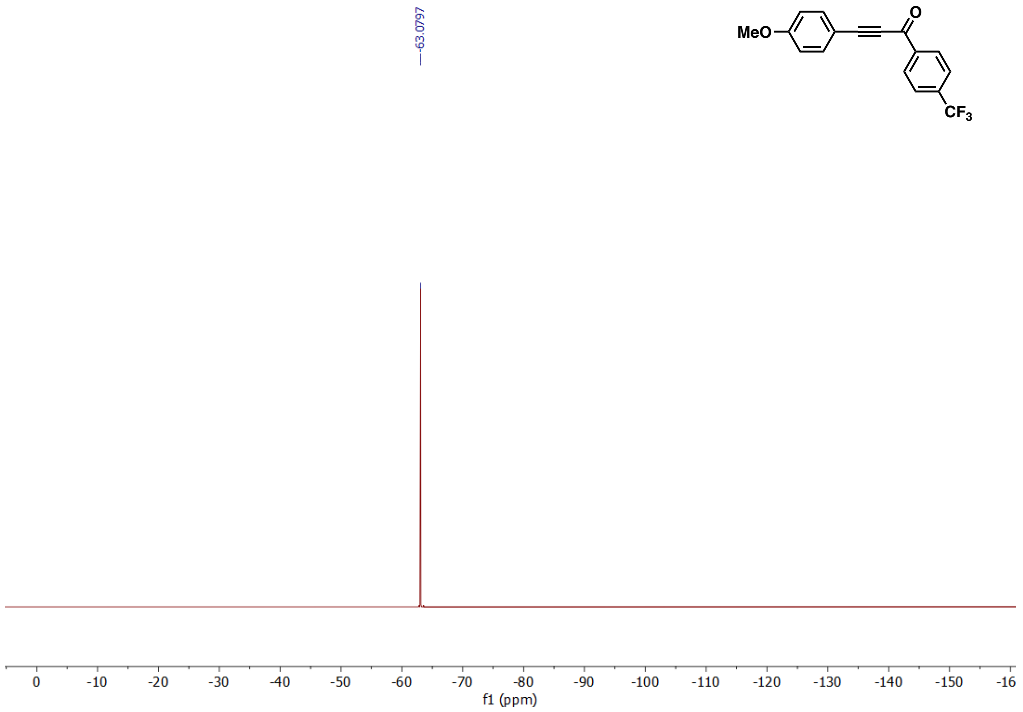
-63.0797
MeO
O
CF3
0 -10 -20 -30 -40 -50 -60 -70 -80 -90 -100 -110 -120 -130 -140 -150 -16
f1 (ppm)

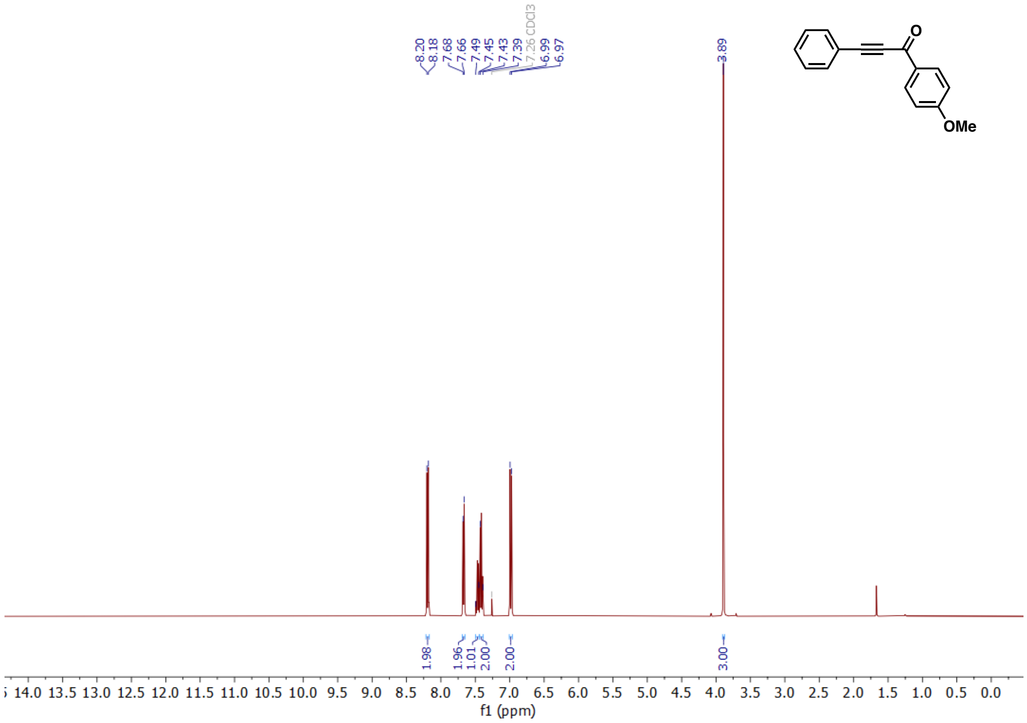
8.20
8.18
7.68
7.66
7.49
7.45
7.43
7.39
7.26 CDCl3
6.99
6.97
3.89
O
OMe
1.98
1.96
1.01
2.00
2.00
3.00
14.0 13.5 13.0 12.5 12.0 11.5 11.0 10.5 10.0 9.5 9.0 8.5 8.0 7.5 7.0 6.5 6.0 5.5 5.0 4.5 4.0 3.5 3.0 2.5 2.0 1.5 1.0 0.5 0.0
f1 (ppm)

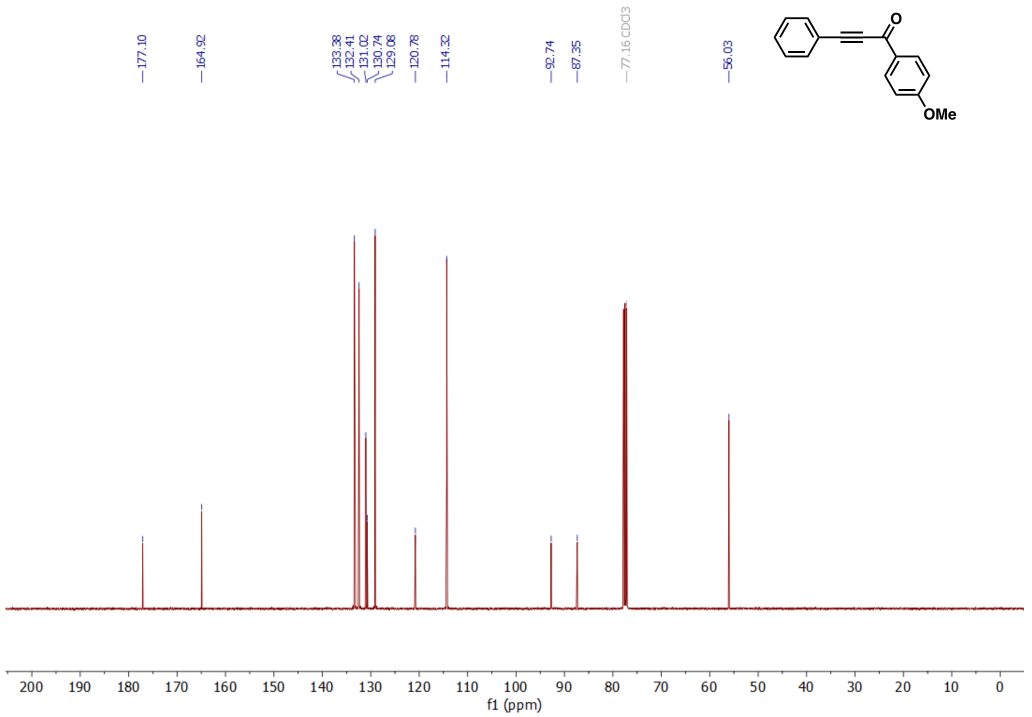
177.10
164.92
133.38
132.41
131.02
130.74
129.08
120.78
114.32
92.74
87.35
77.16 CDCl3
56.03
O
OMe
f1 (ppm)

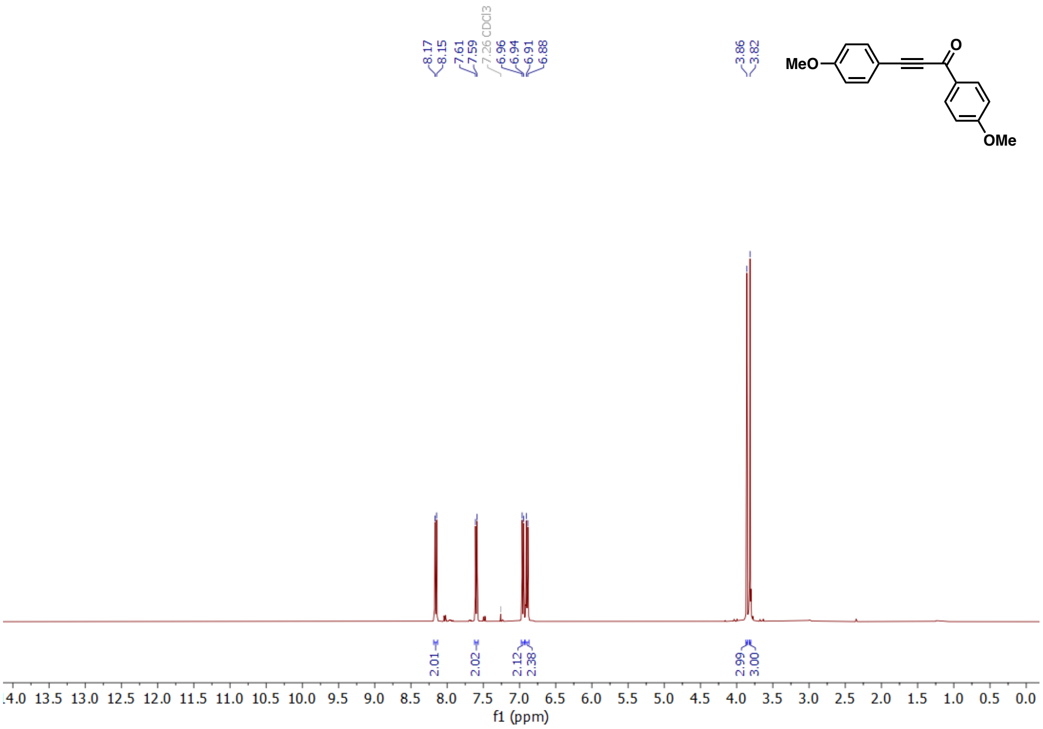
8.17
8.15
7.61
7.59
7.26 CDCl3
6.96
6.94
6.91
6.88
3.86
3.82
MeO
O
OMe
2.01
2.02
2.12
2.38
2.99
3.00
f1 (ppm)

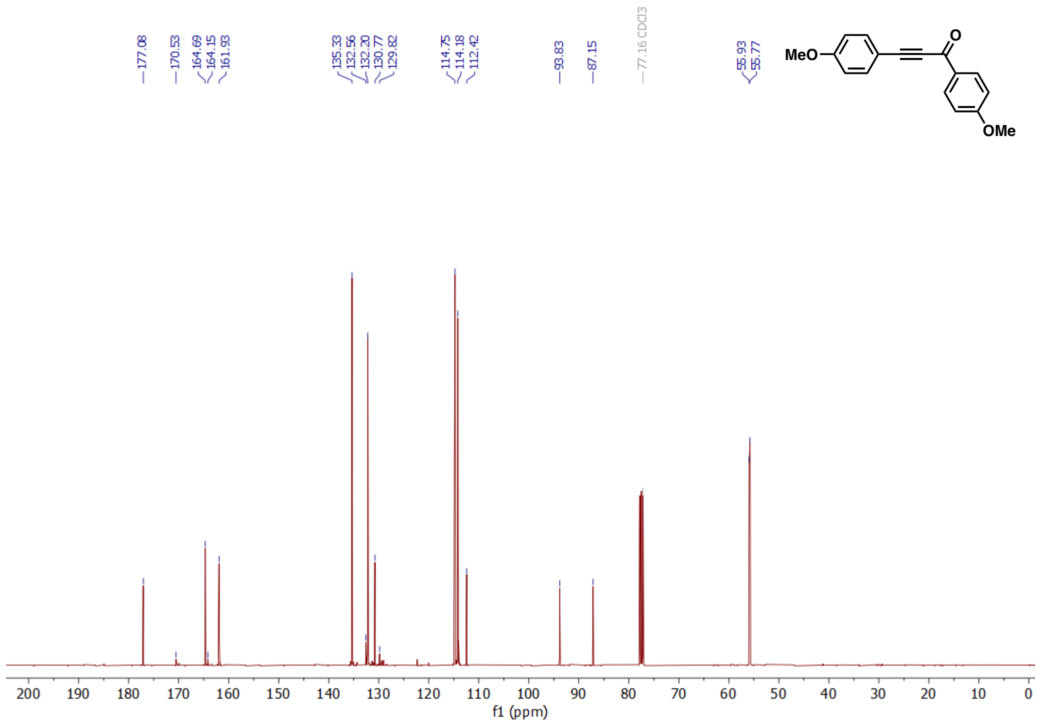

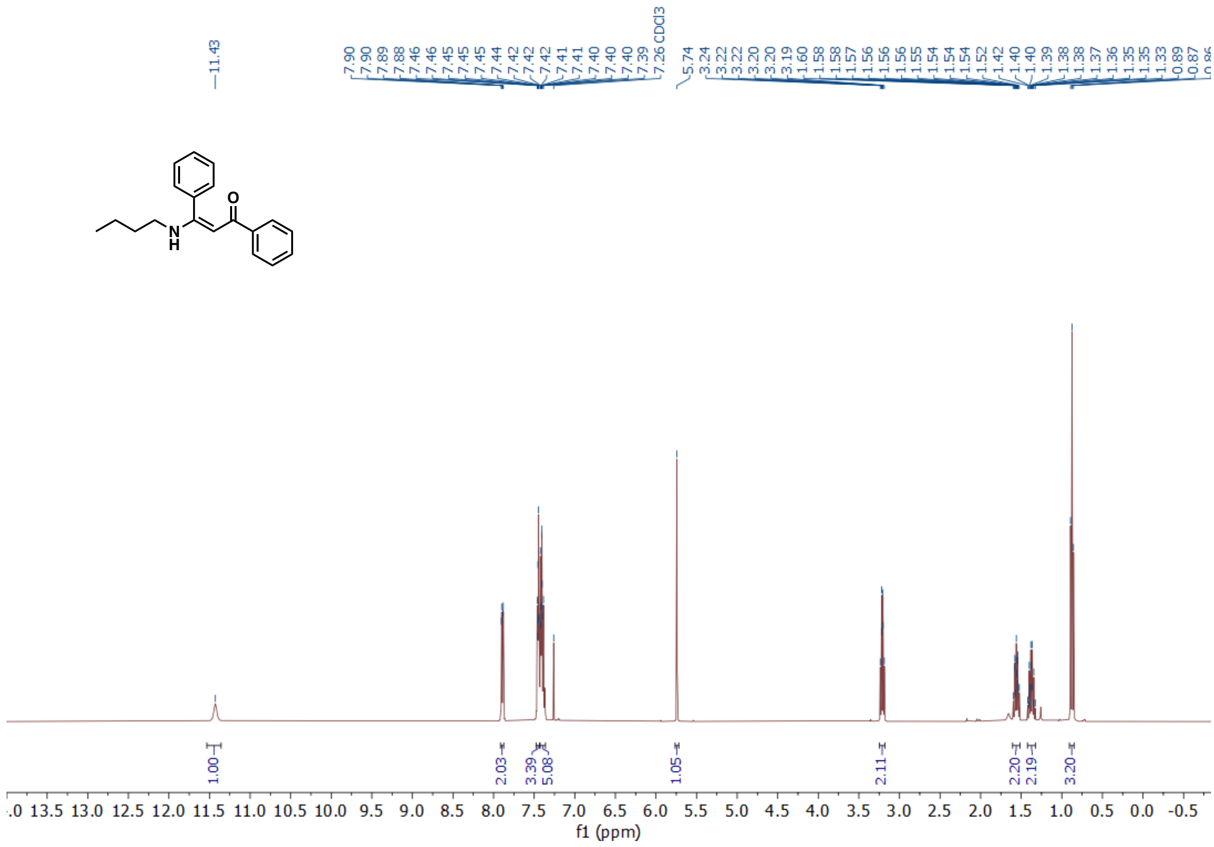

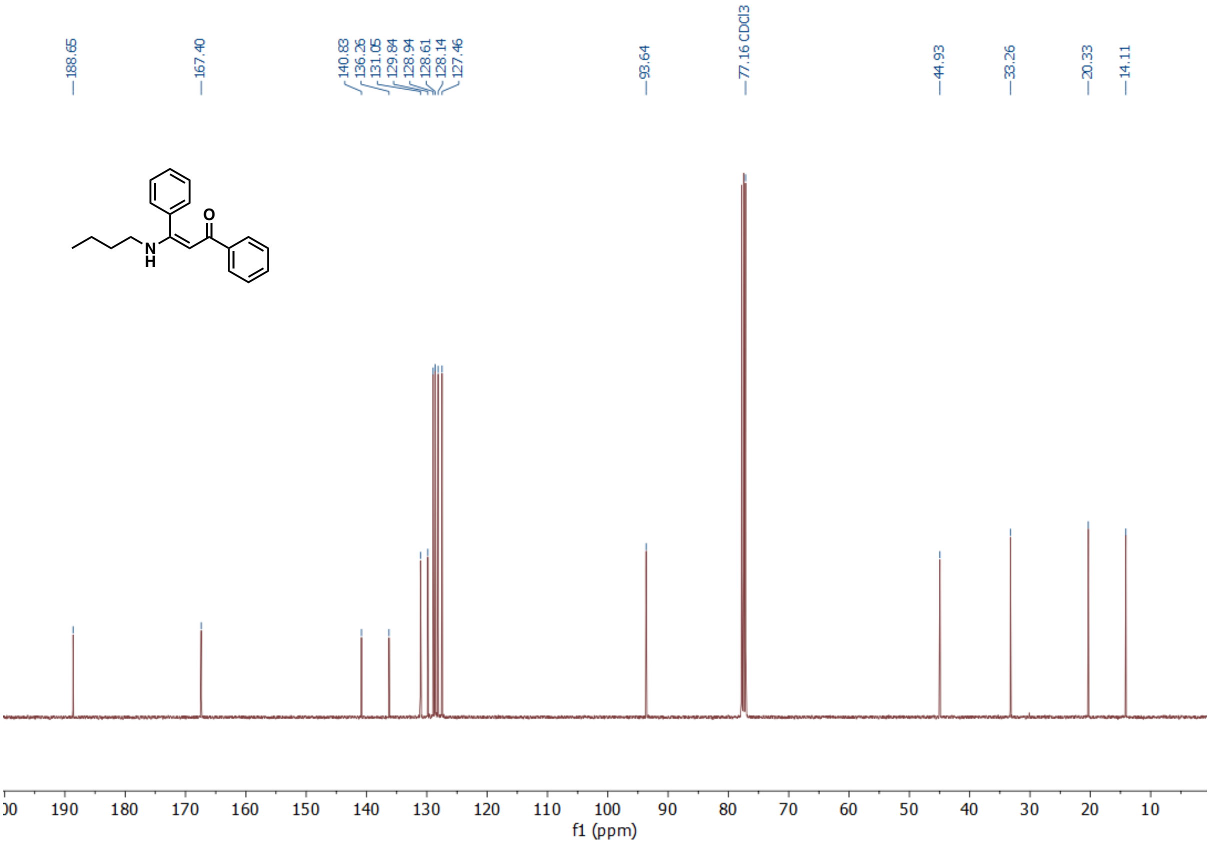
188.65
167.40
140.83
136.26
131.05
129.84
128.94
128.61
128.14
127.46
93.64
77.16 CDCl3
44.93
33.26
20.33
14.11
O
N
H
f1 (ppm)

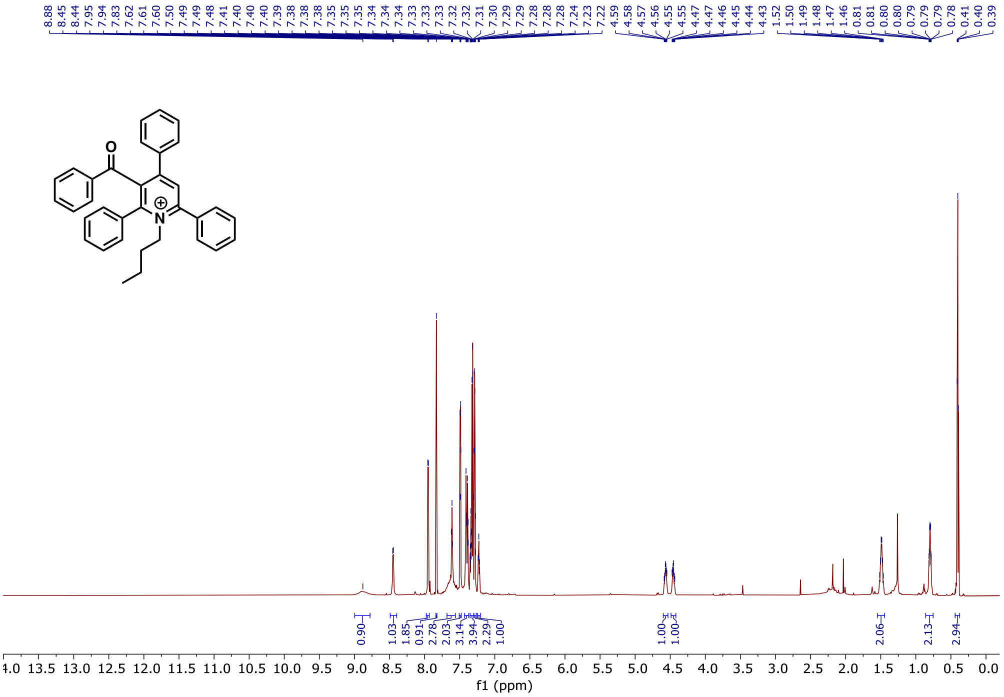
8.88
8.45
8.44
7.95
7.94
7.83
7.62
7.61
7.60
7.50
7.49
7.49
7.48
7.41
7.40
7.40
7.39
7.38
7.38
7.38
7.35
7.35
7.34
7.34
7.34
7.33
7.33
7.33
7.32
7.32
7.31
7.30
7.29
7.29
7.28
7.28
7.24
7.23
7.22
4.59
4.58
4.57
4.56
4.55
4.47
4.47
4.46
4.45
4.44
4.43
1.52
1.50
1.49
1.48
1.47
1.46
0.81
0.81
0.80
0.79
0.79
0.79
0.78
0.41
0.40
0.39
O
N
0.90
1.03
1.85
0.91
2.78
2.03
3.14
3.94
2.29
1.00
1.00
1.00
2.06
2.13
2.94
f1 (ppm)

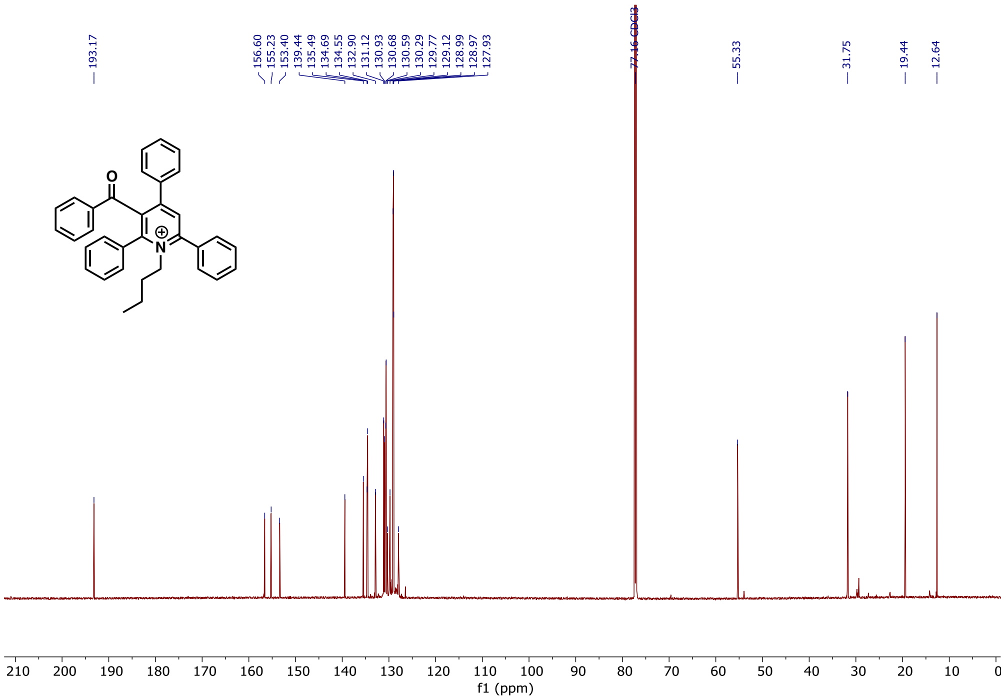
193.17
156.60
155.23
153.40
139.44
135.49
134.69
134.55
132.90
131.12
130.93
130.68
130.59
130.29
129.77
129.12
128.99
128.97
127.93
77.16 CDCl3
55.33
31.75
19.44
12.64
O
N
f1 (ppm)

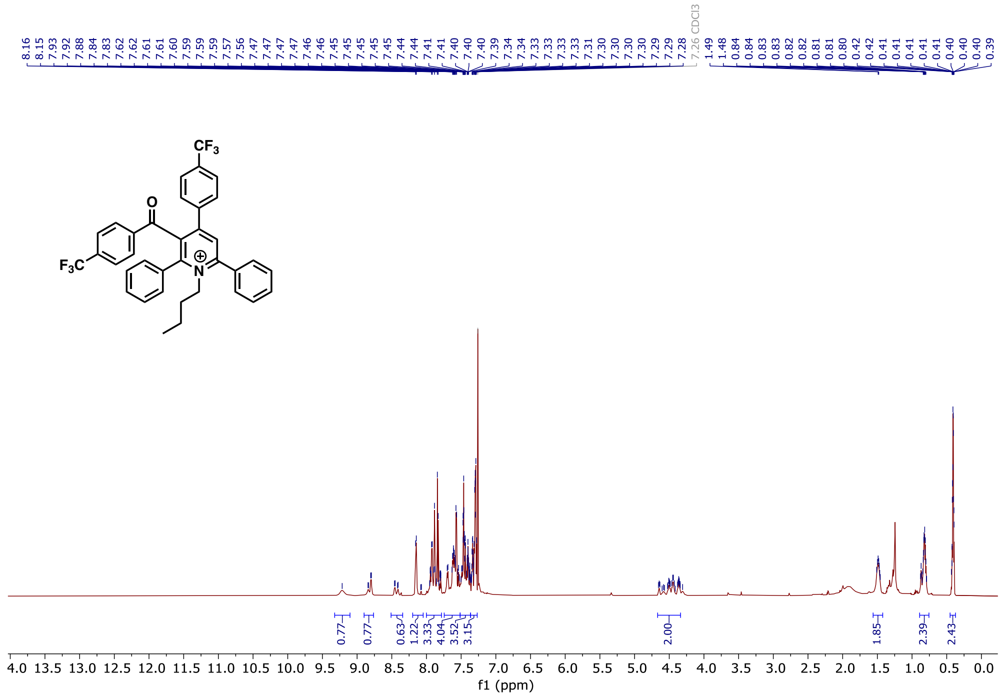
8.16
8.15
7.93
7.92
7.88
7.84
7.83
7.62
7.62
7.61
7.61
7.60
7.59
7.59
7.59
7.57
7.56
7.47
7.47
7.47
7.46
7.45
7.45
7.45
7.45
7.44
7.41
7.41
7.40
7.40
7.40
7.39
7.34
7.34
7.33
7.33
7.33
7.31
7.30
7.30
7.30
7.29
7.28
7.26 CDCl3
1.49
1.48
0.84
0.84
0.83
0.83
0.82
0.82
0.81
0.81
0.80
0.42
0.42
0.41
0.41
0.41
0.41
0.40
0.40
0.40
0.39
CF3
O
F3C
N
0.77
0.77
0.63
1.22
3.33
4.04
3.52
3.15
2.00
1.85
2.39
2.43
f1 (ppm)

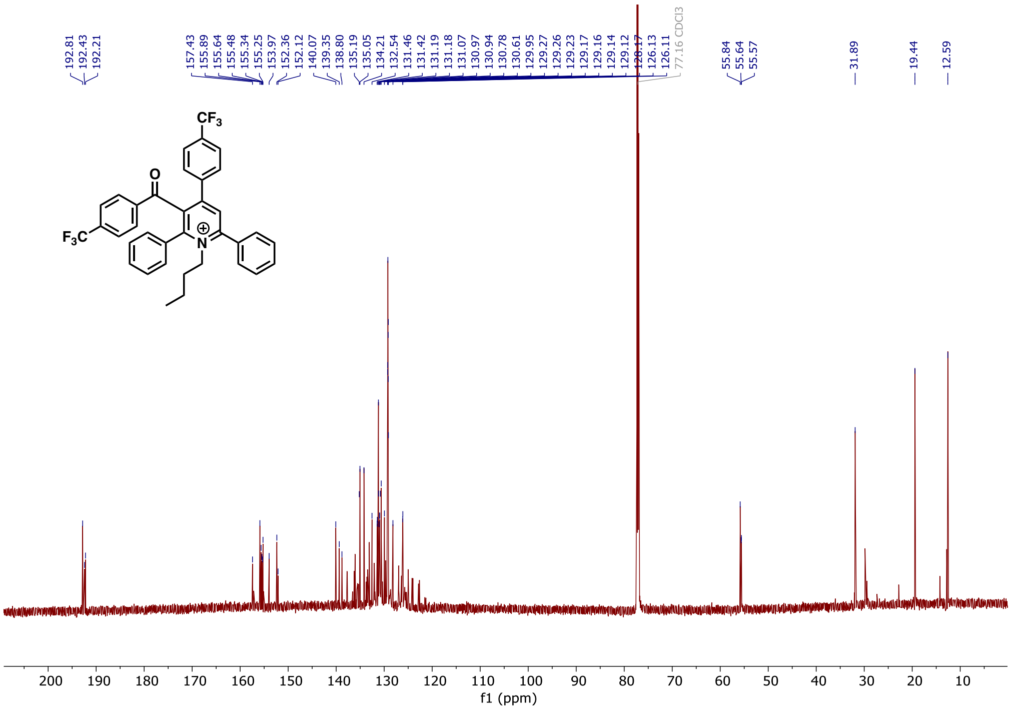
192.81
192.43
192.21
157.43
155.89
155.64
155.48
155.34
155.25
153.97
152.36
152.12
140.07
139.35
138.80
135.19
135.05
134.21
132.54
131.46
131.42
131.19
131.18
131.07
130.97
130.94
130.78
130.61
129.95
129.27
129.26
129.23
129.17
129.16
129.14
129.12
128.17
126.13
126.11
77.16 CDCl3
55.84
55.64
55.57
31.89
19.44
12.59
CF3
O
F3C
N
200 190 180 170 160 150 140 130 120 110 100 90 80 70 60 50 40 30 20 10
f1 (ppm)

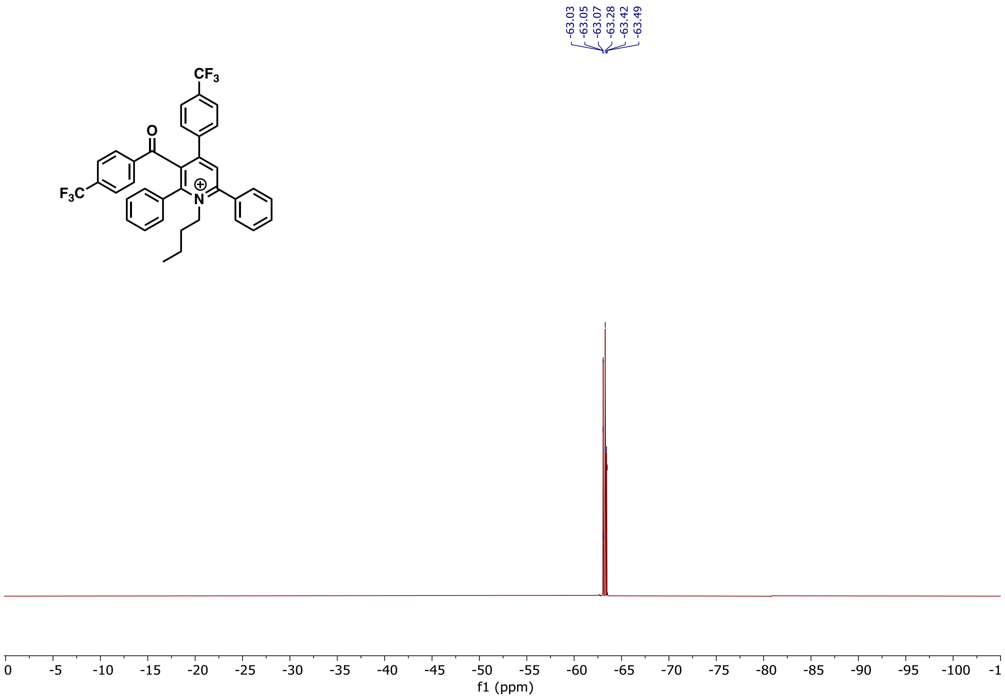
-63.03
-63.05
-63.07
-63.28
-63.42
-63.49
CF3
O
F3C
N
0 -5 -10 -15 -20 -25 -30 -35 -40 -45 -50 -55 -60 -65 -70 -75 -80 -85 -90 -95 -100 -1
f1 (ppm)

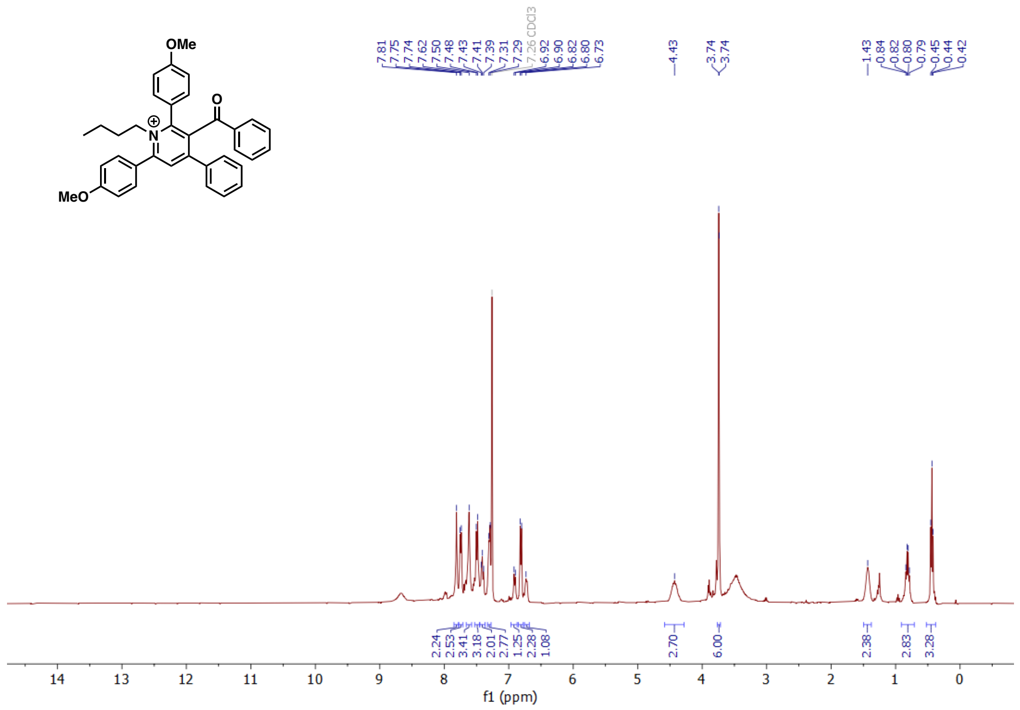
OMe
O
N
MeO
7.81
7.75
7.74
7.62
7.50
7.48
7.43
7.41
7.39
7.31
7.29
7.26 CDCl3
6.92
6.90
6.82
6.80
6.73
4.43
3.74
3.74
1.43
0.84
0.82
0.80
0.79
0.45
0.44
0.42
2.24
2.53
3.41
3.18
2.01
2.77
1.25
2.28
1.08
2.70
6.00
2.38
2.83
3.28
14 13 12 11 10 9 8 7 6 5 4 3 2 1 0
f1 (ppm)

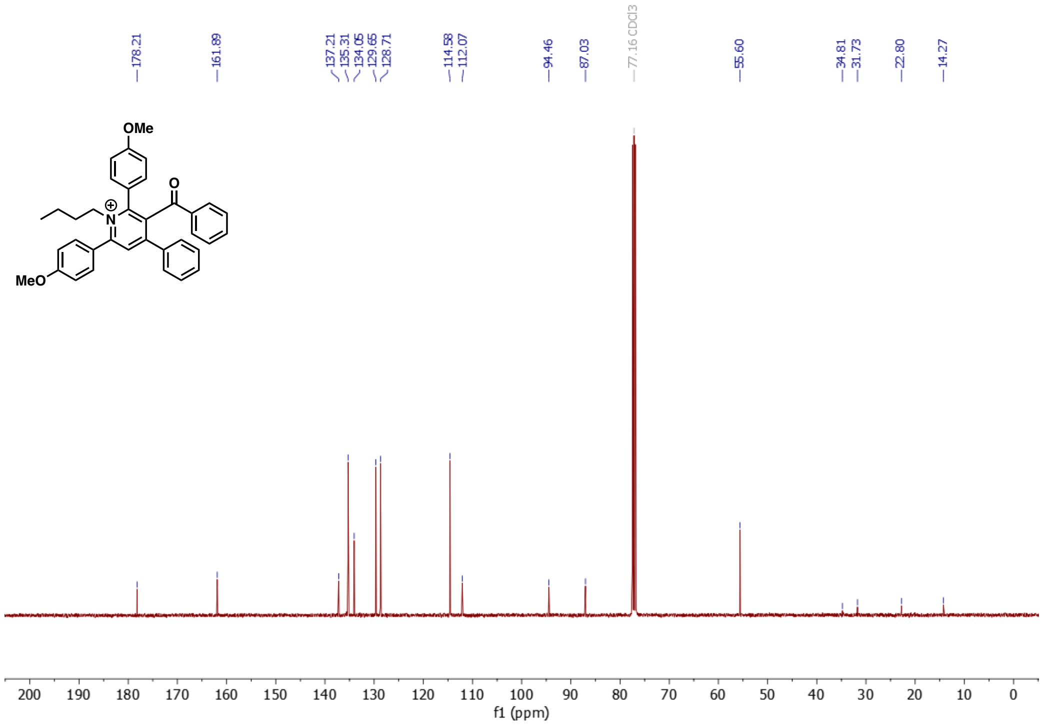
178.21
161.89
137.21
135.31
134.05
129.65
128.71
114.58
112.07
94.46
87.03
77.16 CDCl3
55.60
34.81
31.73
22.80
14.27
OMe
O
N
MeO
200 190 180 170 160 150 140 130 120 110 100 90 80 70 60 50 40 30 20 10 0
f1 (ppm)

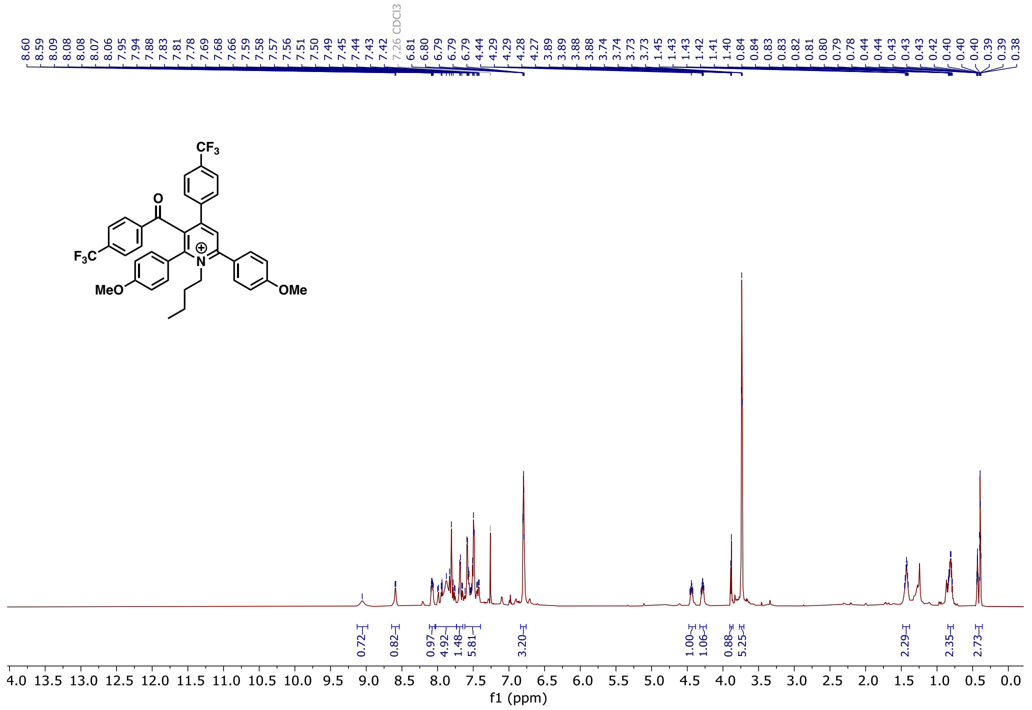
8.60 8.59 8.09 8.08 8.08 8.07 8.06 7.95 7.94 7.88 7.83 7.81 7.78 7.69 7.68 7.66 7.59 7.58 7.57 7.56 7.51 7.50 7.49 7.45 7.44 7.43 7.42 7.26 CDCl3 6.81 6.80 6.79 6.79 6.79 4.44 4.29 4.29 4.28 4.27 3.89 3.89 3.88 3.88 3.74 3.74 3.73 3.73 1.45 1.43 1.43 1.42 1.41 1.40 0.84 0.84 0.83 0.83 0.82 0.81 0.80 0.79 0.78 0.44 0.44 0.43 0.43 0.43 0.42 0.40 0.40 0.39 0.39 0.38
CF3
O
F3C
N
MeO
OMe
0.72 0.82 0.97 4.92 1.48 5.81 3.20 1.00 1.06 0.88 5.25 2.29 2.35 2.73
4.0 13.5 13.0 12.5 12.0 11.5 11.0 10.5 10.0 9.5 9.0 8.5 8.0 7.5 7.0 6.5 6.0 5.5 5.0 4.5 4.0 3.5 3.0 2.5 2.0 1.5 1.0 0.5 0.0
f1 (ppm)

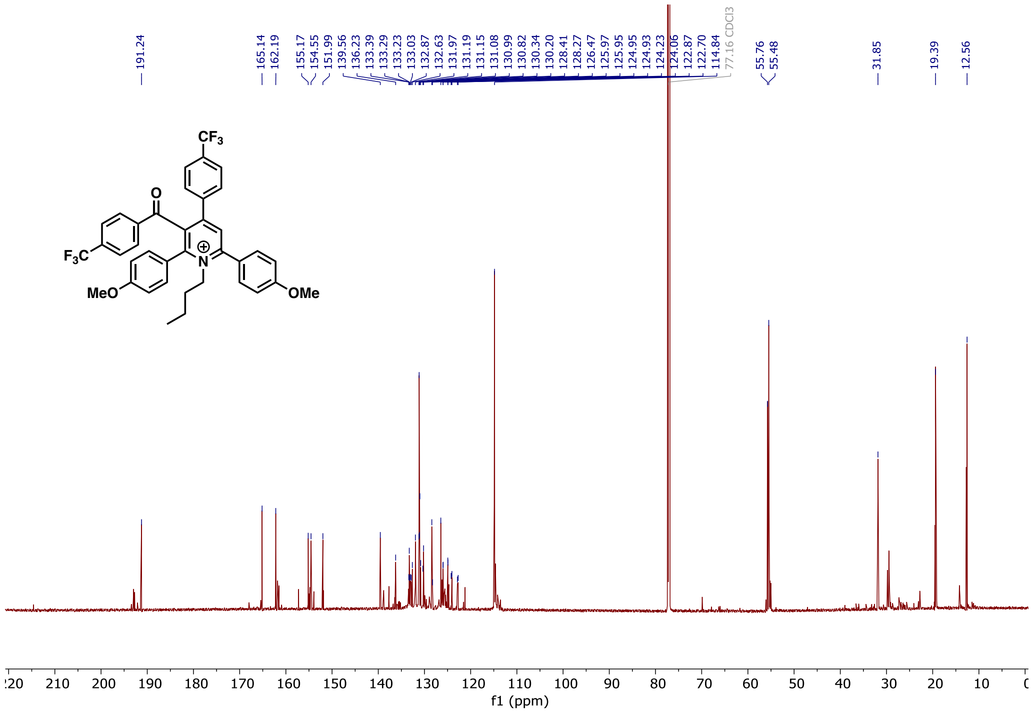
191.24
165.14 162.19 155.17 154.55 151.99 139.56 136.23 133.39 133.29 133.23 133.03 132.87 132.63 131.97 131.19 131.15 131.08 130.99 130.82 130.34 130.20 128.41 128.27 126.47 125.97 125.95 124.95 124.93 124.23 124.06 122.87 122.70 114.84 77.16 CDCl3
55.76 55.48
31.85
19.39
12.56
CF3
O
F3C
N
MeO
OMe
220 210 200 190 180 170 160 150 140 130 120 110 100 90 80 70 60 50 40 30 20 10 0
f1 (ppm)

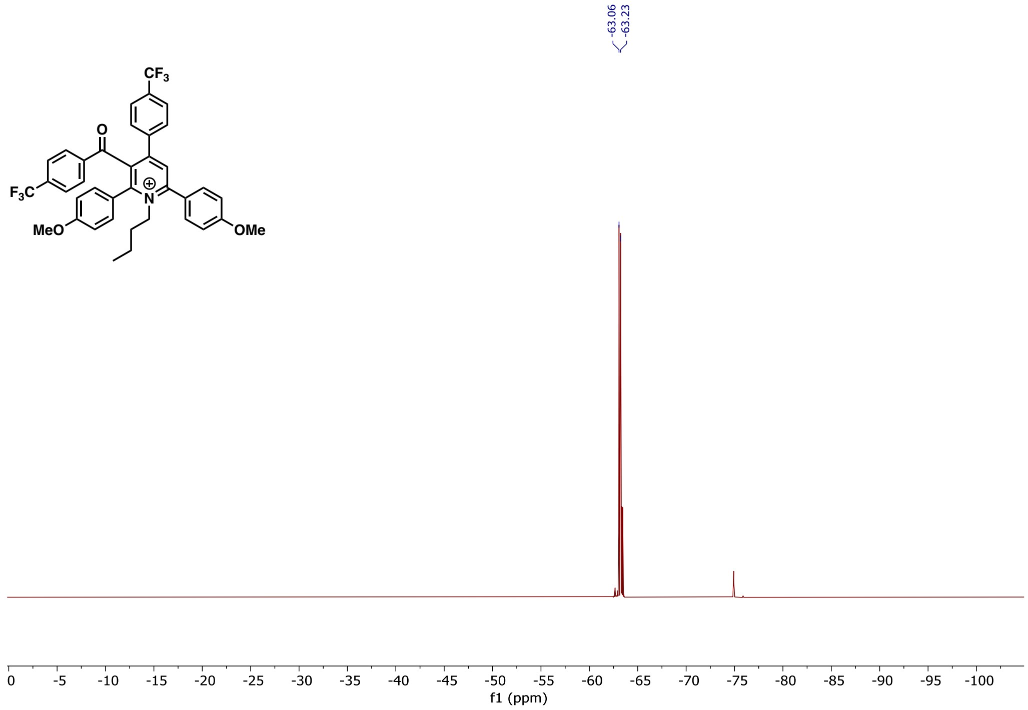
-63.06
-63.23
CF3
O
F3C
N
MeO
OMe
0 -5 -10 -15 -20 -25 -30 -35 -40 -45 -50 -55 -60 -65 -70 -75 -80 -85 -90 -95 -100
f1 (ppm)

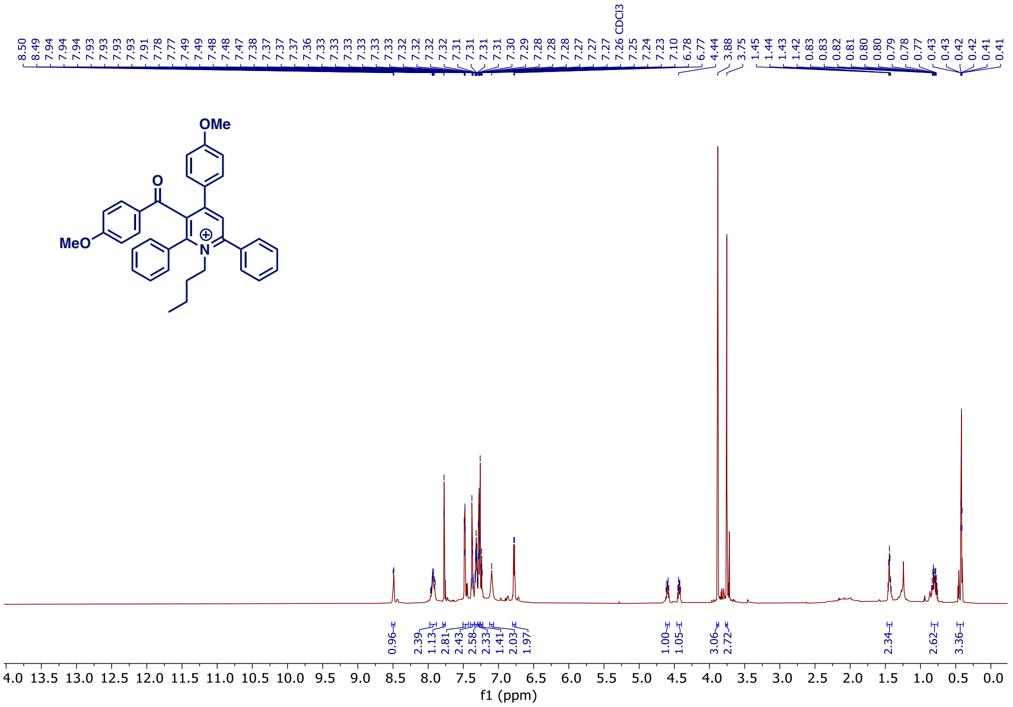
8.50 8.49 7.94 7.94 7.94 7.93 7.93 7.93 7.91 7.78 7.77 7.49 7.49 7.48 7.48 7.47 7.38 7.37 7.37 7.37 7.36 7.33 7.33 7.33 7.33 7.33 7.33 7.32 7.32 7.32 7.32 7.31 7.31 7.31 7.31 7.30 7.29 7.28 7.28 7.28 7.27 7.27 7.26 CDCl3 7.25 7.24 7.23 7.10 6.78 6.77 4.44 3.88 3.75 1.45 1.44 1.43 1.42 0.83 0.83 0.82 0.81 0.80 0.80 0.79 0.78 0.77 0.43 0.43 0.42 0.42 0.41 0.41
OMe
O
MeO
N
0.96 2.39 1.13 2.81 2.43 2.58 2.33 1.41 2.03 1.97 1.00 1.05 3.06 2.72 2.34 2.62 3.36
4.0 13.5 13.0 12.5 12.0 11.5 11.0 10.5 10.0 9.5 9.0 8.5 8.0 7.5 7.0 6.5 6.0 5.5 5.0 4.5 4.0 3.5 3.0 2.5 2.0 1.5 1.0 0.5 0.0
f1 (ppm)

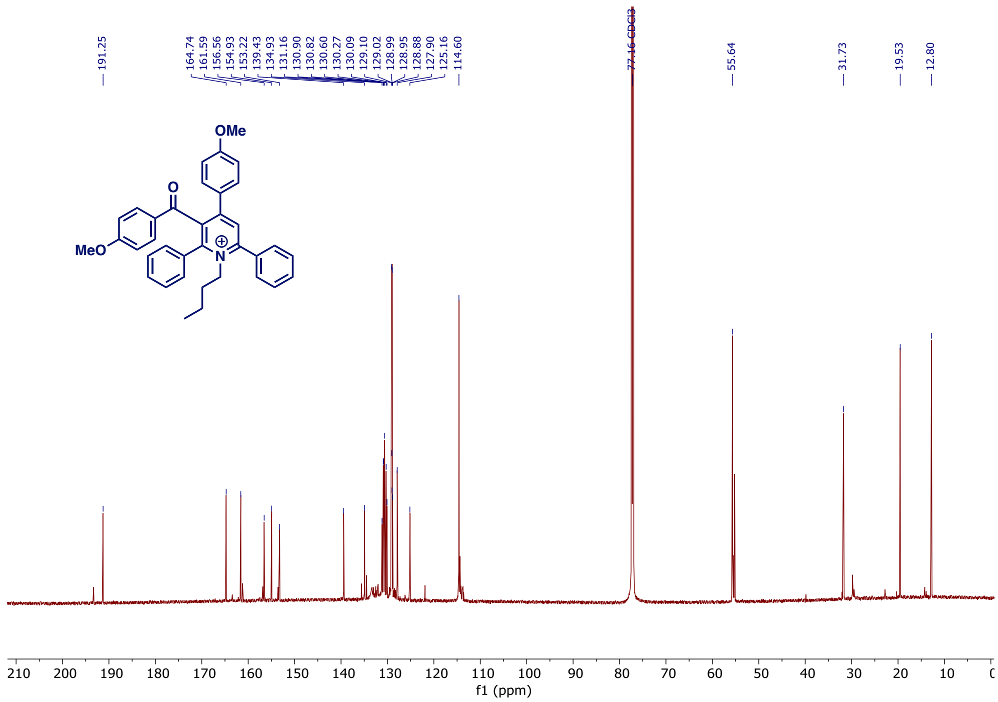
191.25
164.74
161.59
156.56
154.93
153.22
139.43
134.93
131.16
130.90
130.82
130.60
130.27
130.09
129.10
129.02
128.99
128.95
128.88
127.90
125.16
114.60
77.16 CDCl3
55.64
31.73
19.53
12.80
OMe
O
MeO
N
210 200 190 180 170 160 150 140 130 120 110 100 90 80 70 60 50 40 30 20 10
f1 (ppm)

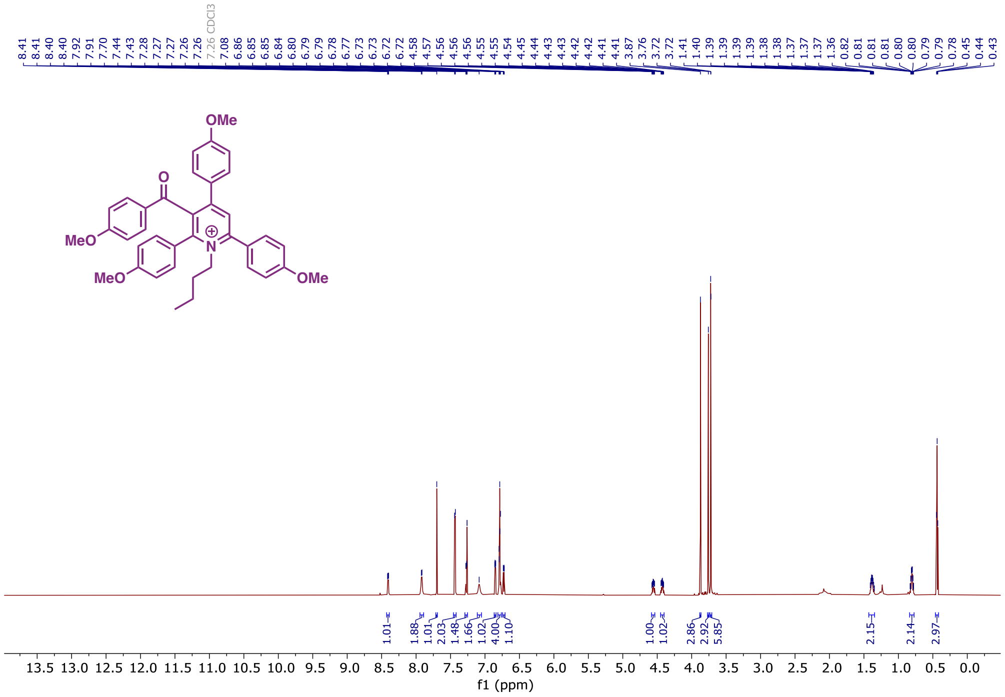
8.41
8.41
8.40
7.92
7.91
7.70
7.44
7.43
7.28
7.27
7.26
7.26 CDCl3
7.08
6.86
6.85
6.84
6.80
6.79
6.79
6.78
6.77
6.73
6.73
6.72
6.72
4.58
4.57
4.56
4.56
4.55
4.55
4.54
4.45
4.44
4.43
4.43
4.42
4.42
4.41
4.41
3.87
3.76
3.72
3.72
1.41
1.40
1.39
1.39
1.39
1.38
1.37
1.37
1.37
1.36
0.82
0.81
0.81
0.81
0.80
0.80
0.79
0.79
0.78
0.45
0.44
0.43
OMe
O
MeO
MeO
N
OMe
1.01
1.88
1.01
2.03
1.48
1.66
1.02
4.00
1.10
1.00
1.02
2.86
2.92
5.85
2.15
2.14
2.97
13.5 13.0 12.5 12.0 11.5 11.0 10.5 10.0 9.5 9.0 8.5 8.0 7.5 7.0 6.5 6.0 5.5 5.0 4.5 4.0 3.5 3.0 2.5 2.0 1.5 1.0 0.5 0.0
f1 (ppm)

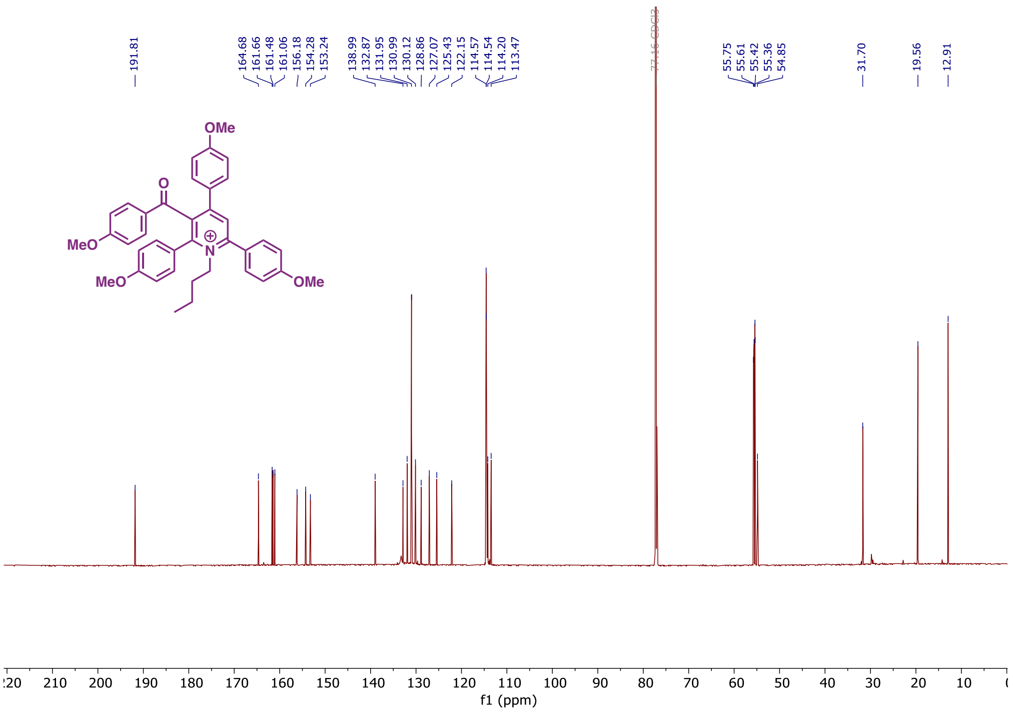
OMe
O
MeO
MeO
N
OMe
f1 (ppm)

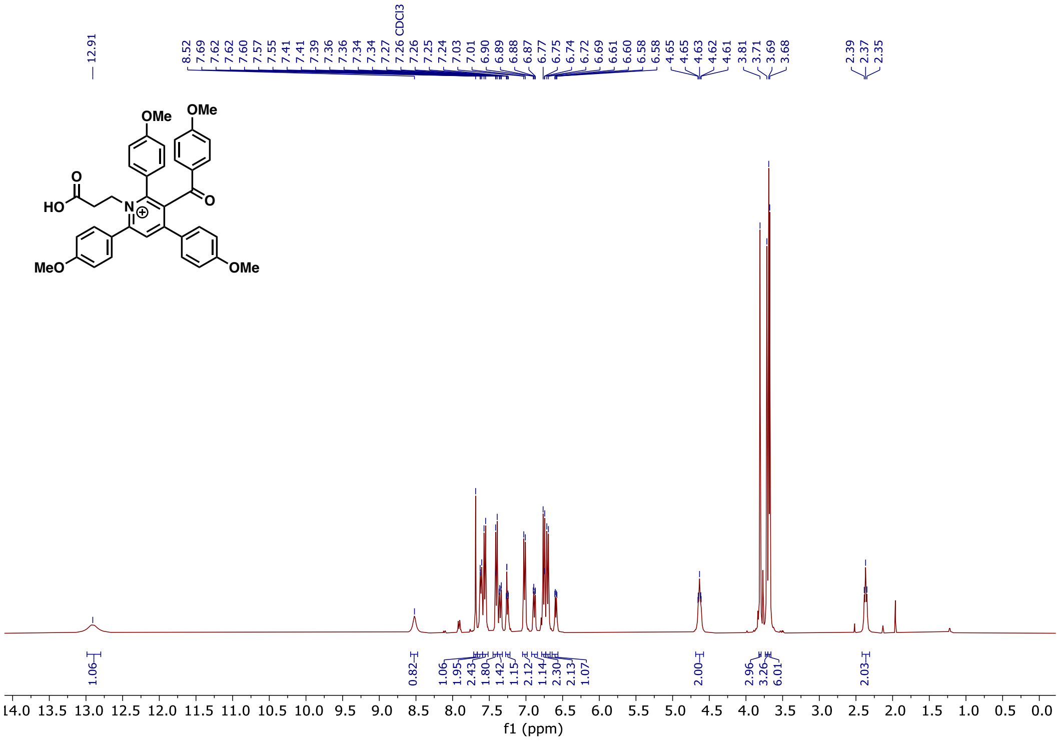
OMe
OMe
O
HO
N
O
MeO
OMe
f1 (ppm)

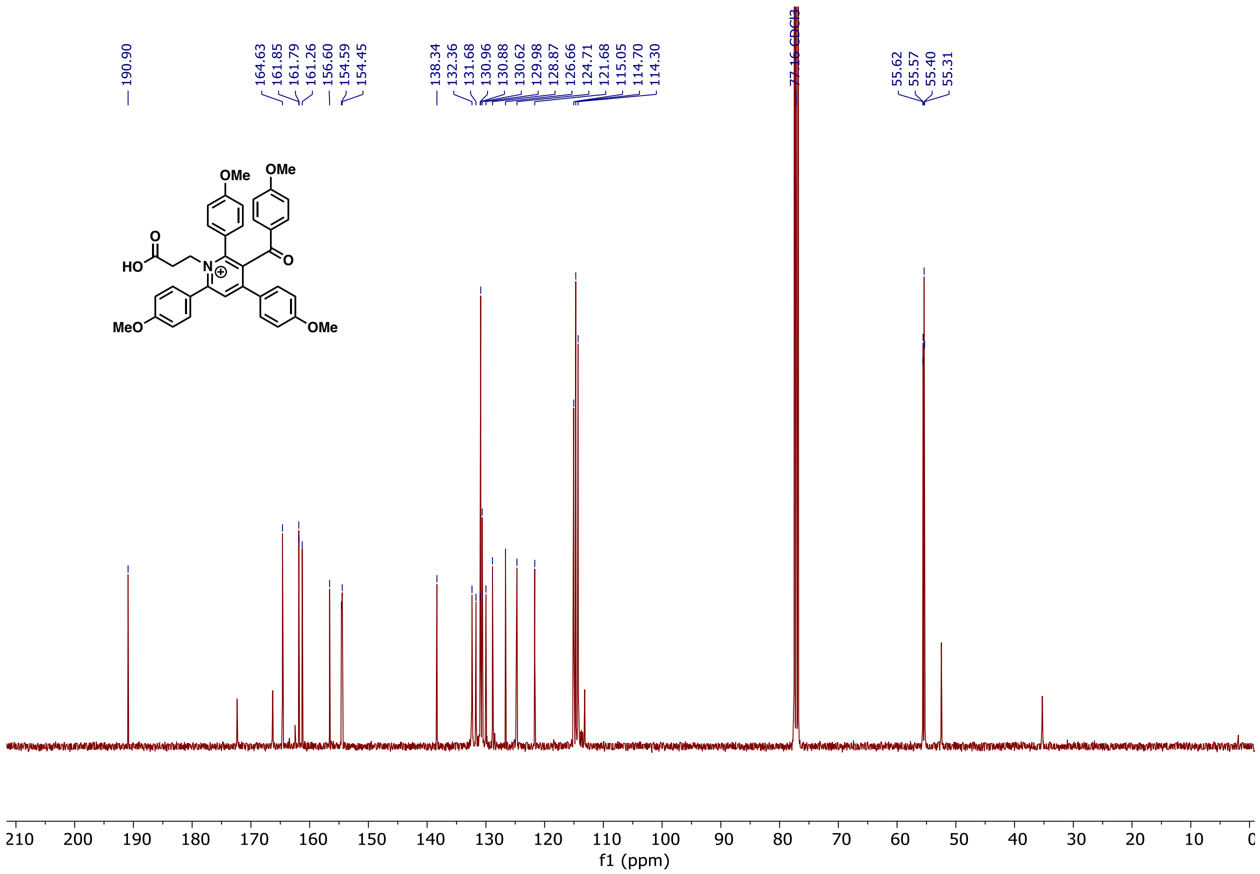

XXXVI. References.

1. Chan, W. C.; White, P. D. Fmoc solid phase peptide synthesis: A practical approach, *Oxford Univ. Press*, New York, **2000**.

2. Lovell, T. C.; Branchaud, B. P.; Jasti, R. An Organic Chemist's Guide to Fluorophores – Understanding Common and Newer Non-Planar Fluorescent Molecules for Biological Applications. *Eur. J. Org. Chem.* **2024**, *27*, e202301196.3.

3. Cox, R. J.; Ritson, D. J.; Dane, T. A.; Berge, J.; Charmant, J. P. H.; Kantacha, A. Room Temperature Palladium Catalysed Coupling of Acyl Chlorides with Terminal Alkynes. *Chem. Commun.* **2005**, *8*, 1037-1039.
